# Human LY9 governs CD4^+^ T-cell IFN-γ immunity to *Mycobacterium tuberculosis*

**DOI:** 10.1126/sciimmunol.ads7377

**Published:** 2025-05-30

**Authors:** Masato Ogishi, Julia Puchan, Rui Yang, Andrés Augusto Arias, Ji Eun Han, Tina Nguyen, Rebeca Gutiérrez-Cózar, Clément Conil, Yoann Seeleuthner, Darawan Rinchai, Peng Zhang, Khoren Ponsin, Matthieu Chaldebas, Yi Feng, Anna-Lena Neehus, Ottavia M. Delmonte, Taushif Khan, Nils Landegren, Daniel Eriksson, Jonathan Bohlen, Jessica N. Peel, Iris Fagniez, Simon J. Pelham, Wei-Te Lei, Maya Chrabieh, Candice Laine, Hind Ouair, Ibtihal Benhsaien, Ahmed Abid, Ismail Abderrhamani Ghorfi, Hicham Souhi, Hanane Ouazzani, Rafik Aniss, Sean D. Riminton, Olle Kämpe, Stuart E. Turvey, Nico Marr, Luigi D. Notarangelo, Nevin Hatipoglu, Aziz Bousfiha, Tayfun Ozcelik, Jamila El Baghdadi, Aurelie Cobat, Cindy S. Ma, Laurent Abel, Anne Puel, Jacinta Bustamante, Pablo Engel, Philippe Gros, Stuart G. Tangye, Federica Sallusto, Stéphanie Boisson-Dupuis, Jean-Laurent Casanova

**Affiliations:** 1 St. Giles Laboratory of Human Genetics of Infectious Diseases, Rockefeller Branch, Rockefeller University, New York, NY, USA.; 2 The David Rockefeller Graduate Program, Rockefeller University, New York, NY, USA.; 3 Institute of Microbiology, ETH Zürich, Zürich, Switzerland.; 4 Primary Immunodeficiencies Group, University of Antioquia UdeA, Medellin, Colombia.; 5 School of Microbiology, University of Antioquia UdeA, Medellin, Colombia.; 6 Garvan Institute of Medical Research, Darlinghurst, Australia.; 7 School of Clinical Medicine, Faculty of Medicine and Health, University of New South Wales Sydney, Kensington, Australia.; 8 Immunology Unit, Department of Biomedical Sciences, Faculty of Medicine and Medical Sciences, University of Barcelona, Barcelona, Spain.; 9 Laboratory of Human Genetics of Infectious Diseases, Necker Branch, INSERM U1163, Paris, France.; 10 Imagine Institute, Paris Cité University, Paris, France.; 11 Immune Deficiency Genetics Section, Laboratory of Host Defenses, Division of Intramural Research, National Institute of Allergy and Infectious Diseases, National Institutes of Health, Bethesda, MD, USA.; 12 Department of Human Immunology, Research Branch, Sidra Medicine, Doha, Qatar.; 13 Center for Molecular Medicine, Department of Medicine (Solna), Karolinska Institute, Stockholm, Sweden.; 14 Clinical Immunology, Autoimmunity and Inflammation Laboratory (LICIA), Faculty of Medicine and Pharmacy, Hassan II University, Casablanca, Morocco.; 15 Department of Pediatric Infectious Diseases, Clinical Immunology Unit, Children’s Hospital, Averroes University Hospital Center, Casablanca, Morocco.; 16 Laboratory of Clinical Immunology, Inflammation, and Allergy, Faculty of Medicine and Pharmacy of Casablanca, King Hassan II University, Casablanca, Morocco.; 17 Department of Pulmonology, Mohammed V Military Hospital, Rabat, Morocco.; 18 Medical and Pharmacy School of Rabat, Mohammed V University, Rabat, Morocco.; 19 Genetics Unit, Mohamed V Military Hospital, Hay Riad, Rabat, Morocco.; 20 Immunology Department, Concord Repatriation General Hospital, Hospital Road, Concord, New South Wales, Australia.; 21 University of Sydney, Camperdown, New South Wales, Australia.; 22 Department of Endocrinology, Karolinska University Hospital, Stockholm, Sweden.; 23 Department of Pediatrics, British Columbia Children’s Hospital and The University of British Columbia, Vancouver, Canada.; 24 College of Health and Life Sciences, Hamad Bin Khalifa University, Doha, Qatar.; 25 Pediatric Infectious Diseases Unit, Bakirkoy Dr Sadi Konuk Training and Research Hospital, University of Health Sciences, Istanbul, Turkey.; 26 Department of Molecular Biology and Genetics, Bilkent University, Ankara, Turkey.; 27 Clinical Immunogenomics Research Consortium Australasia, Sydney, Australia.; 28 McGill Research Center on Complex Traits, Montreal, Quebec, Canada.; 29 Department of Biochemistry, McGill University, Montreal, Quebec, Canada.; 30 Center of Medical Immunology, Institute for Research in Biomedicine, Faculty of Biomedical Sciences, University of Italian Switzerland, Bellinzona, Switzerland.; 31 Department of Pediatrics, Necker Hospital for Sick Children, Paris, France.; 32 Howard Hughes Medical Institute, New York, NY, USA.

## Abstract

CD4^+^ T cells are indispensable for optimal immunity to *Mycobacterium tuberculosis* (*M.tb*), a pathogen that triggers tuberculosis (TB) in humans. *M.tb*-specific human CD4^+^ T cells are known to polarize toward an IFN-γ-producing, CCR4^−^CCR6^+^CXCR3^+^T-bet^+^RORγT^+^ T_H_1* memory phenotype. We report that autosomal recessive deficiency of the human lymphocytic surface receptor LY9 (SLAMF3, CD229), which is found in less than 10^−5^ individuals in the general population, underlies TB in three unrelated patients due to selective impairment in IFN-γ production by T_H_1* cells. T_H_1* cells express higher levels of LY9 than other CD4^+^ T cells. Mechanistically, LY9 polarizes naïve CD4^+^ T cells toward memory T_H_1* cells by inducing T-bet via signaling lymphocytic activation molecule (SLAM)-associated protein (SAP) and RORγT without SAP. LY9 costimulation enhances TCR-driven IFN-γ production of memory T_H_1*, but not T_H_1, cells in a T-cell-intrinsic manner via NFAT1 and RORγT. LY9 is likely to govern an optimal T_H_1*-cell- and IFN-γ-dependent protective immunity to *M.tb* in humans.

## One sentence summary

Impaired CD4^+^ T cell-dependent IFN-γ immunity in LY9-deficient humans underlies tuberculosis.

## Introduction

Tuberculosis (TB), an infectious disease caused by *Mycobacterium tuberculosis* (*M.tb*), is a major threat to global health (1). According to WHO estimates, ~10.6 million new cases and ~1.6 million deaths were recorded in 2021, of which 0.9 million and 0.2 million, respectively, concerned individuals coinfected with human immunodeficiency virus 1 (HIV-1) (2). However, TB occurs in only 5~10% of *M.tb*-infected HIV-1-negative individuals (3). Classical genetic studies have suggested a crucial role for human genetic determinants of TB as opposed to *M.tb* latency or clearance (4–7). Mendelian susceptibility to Mycobacterial Disease (MSMD) is observed in ~1/50,000 live births and characterized by selective vulnerability to weakly virulent *M. bovis* Bacillus Calmette-Guérin (BCG) vaccine substrains and environmental mycobacteria (EM). Studies of MSMD in the mid-1990s used a forward genetic analysis of the pathophysiology of mycobacterial disease in humans (6, 8–13) and identified a core mechanism of MSMD in patients with most genetic etiologies appears to involve an impairment of IFN-γ production (*IFNG, IL12B*, *IL12RB1*, *IL12RB2*, *IL23R*, *TYK2*, *ISG15*, *RORC*, *TBX21*, and *MCTS1*), the cellular response to IFN-γ (*IFNGR1*, *IFNGR2*, *STAT1*, *JAK1*, *CYBB*, and *CCR2*), or both (*IKBKG*, *IRF1*, *IRF8*, and *SPPL2A*) (14–18). The mechanism of MSMD in patients with ZNFX1 or USP18 deficiencies remains unclear (19, 20).

Genetic lesions associated with a complete or near-complete lack of IFN-γ immunity (biallelic null mutations of *IFNG*, *IFNGR1*, *IFNGR2, STAT1,* and *IRF1*) display complete penetrance for MSMD. By contrast, patients with autosomal recessive (AR) complete IL-12Rβ1 (21–25) or TYK2 deficiency (26, 27) occasionally present TB following infection with *M.tb*, which is ~1,000 times more virulent than BCG and EM, during childhood or adolescence, rather than displaying MSMD during the first few years of life. IFN-γ production by the leukocytes of these patients is impaired but not abolished. Homozygosity for rare and common TYK2 variants selectively impairing IL-23-dependent IFN-γ production confers a predisposition to TB with high penetrance and a predisposition to MSMD with low penetrance (27–30). We have also described one patient with AR complete PD-1 deficiency (31) and three patients with AR complete ITK deficiency (32) who suffered from TB but not MSMD. At the cellular level, these patients had impaired IFN-γ production similar to or slightly milder than that observed in patients with IL-12Rβ1 or TYK deficiency. Finally, the crucial role of IFN-γ in human antimycobacterial immunity is highlighted by the efficacy of recombinant IFN-γ as a treatment in patients with nontuberculous mycobacterial diseases lacking a genetic diagnosis (33), MSMD (34, 35), and TB (32). These observations suggest that profound impairments of IFN-γ immunity result in a predisposition to MSMD and TB, whereas partial impairments of IFN-γ immunity confer a predisposition to TB only (18).

*M.tb* is a facultative intracellular pathogen that thrives in host macrophages (36). IFN-γ, a macrophage-activating lymphokine (37), has a potent bacteriostatic effect on *M.tb*-infected human macrophages *in vitro* (38). The impairment of cellular responses to IFN-γ, therefore, renders macrophages incapable of restricting mycobacterial growth (8, 9, 15, 39). IFN-γ can be produced by most innate (natural killer [NK], innate lymphoid cell group 1 [ILC1]), innate-like adaptive (mucosal-associated invariant T [MAIT], invariant natural killer T [iNKT], γδ T), and purely adaptive (CD4^+^, CD8^+^) T lymphocytes, but the nature of the IFN-γ-producing cells essential for defense against *M.tb* and the molecules responsible for inducing IFN-γ in these cells remain largely unknown. CD4^+^ αβ T lymphocytes are likely to play a key role, as diseases caused by weakly virulent mycobacteria and the more virulent *M.tb* are common manifestations of acquired immunodeficiency syndrome (AIDS) (40) and idiopathic CD4^+^ T lymphocytopenia (41). Moreover, RORγ/RORγT and SPPL2a deficiencies, both underlying MSMD, impair IFN-γ production by T_H_1* cells (also known as T_H_1/17 cells), a CCR4^−^CCR6^+^CXCR3^+^ subset of CD4^+^ αβ T helper cells that typically responds to mycobacterial antigens (42–44), suggesting that human T_H_1* cells play an essential role in antimycobacterial immunity. However, one T-bet-deficient patient suffered from MSMD despite having normal numbers of T_H_1* cells with adequate IFN-γ-producing capacity, suggesting that *Mycobacterium*-specific T_H_1* cells alone cannot protect the host against BCG. The susceptibility of this T-bet-deficient patient to mycobacterial disease was attributed to decreases in the number and IFN-γ-producing capacity of other lymphocyte subsets, particularly CCR4^−^CCR6^−^CXCR3^+^ CD4^+^ αβ T helper cells (T_H_1 cells), innate-like adaptive T [Vδ2^+^ γδ T, MAIT, iNKT] lymphocytes, and NK lymphocytes (45). These findings suggest that human adaptive (T_H_1 and T_H_1*) and innate or innate-like adaptive (Vδ2^+^ γδ T, MAIT, iNKT, and NK) lymphocytes mediate non-redundant and cooperative layers of IFN-γ-mediated immunity to weakly and more virulent mycobacteria. We investigated the cellular basis of IFN-γ immunity to *M.tb* by searching for previously unknown inborn errors of immunity (IEI) in patients with unexplained TB without MSMD.

## Results

### Homozygosity for rare frameshift variants of LY9 in three unrelated TB patients

**Figure 1.**
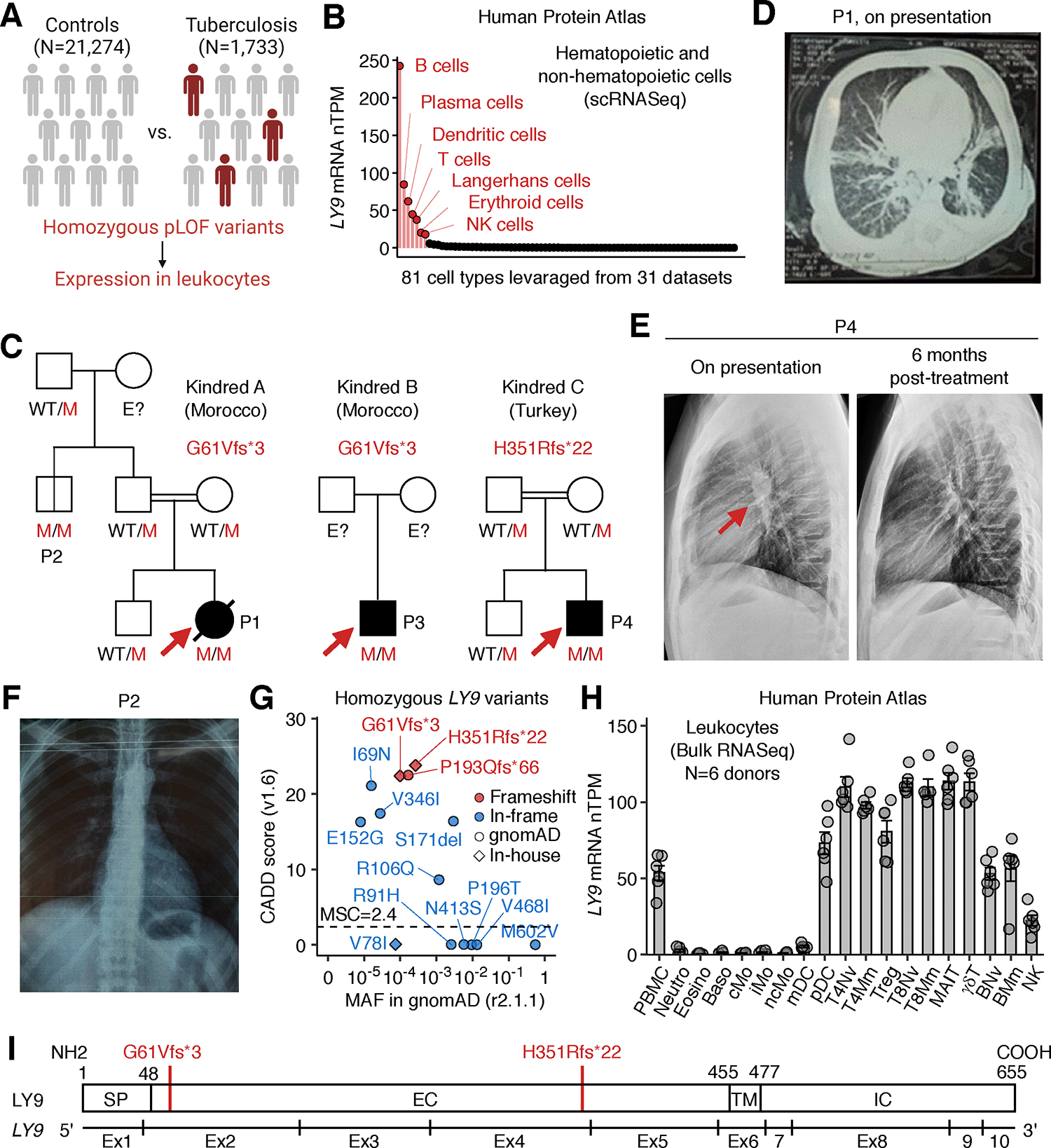
Autosomal recessive LY9 deficiency. (A) Search for homozygous pLOF variants displaying enrichment in TB patients. (B) Normalized *LY9* mRNA levels in various human cell subsets. Data were retrieved from the Human Protein Atlas (73). (C) Pedigree of the kindreds. Black symbols indicate affected individuals. Genotypes for *LY9* are also shown. WT: wild-type. M: mutant. E?: unknown. (D) Thoracic computed tomography scans of P1 on presentation showing bilateral lobar consolidations and atelectasis in the left lung. (E) Chest X ray of P4 showing mediastinal tuberculous lymphadenitis (arrows), which improved after 6 months of anti-TB therapy. (F) A chest X ray for P2 (P1’s paternal uncle) taken at the age of 28 years; this individual remains healthy at the age of 29 years. (G) Population genetics of *LY9*. The minor allele frequency (MAF) and combined annotation-dependent depletion (CADD) scores for all homozygous non-synonymous *LY9* variants found in the gnomAD database or our in-house cohort are depicted. The CADD scores of 22.4 for c.182del (G61Vfs*3) and 23.8 for c.1052_1053del (H351Rfs*22) are well above the mutation significance cutoff (MSC) of 2.4 (79, 80) (horizontal dotted line). (H) Normalized *LY9* mRNA levels in various human immune cell subsets. Data were retrieved from the Human Protein Atlas (73). *N*=6 donors. Bars represent the mean and SEM. (I) A protein-level representation of the patients’ mutations. SP: signal peptide. EC: extracellular domain. TM: transmembrane domain. IC: intracellular domain. The sequences corresponding to the various exons and their boundaries are also shown.

We systematically searched for rare (minor allele frequency (MAF)<0.01), homozygous, predicted loss-of-function (pLOF) variants in our in-house TB cohort (*N*=1,733) (Fig. 1A; Methods). We found three unrelated patients with TB who were homozygous for pLOF variants of *LY9*, a gene expressed exclusively in hematopoietic cells (Fig. 1B and C, fig. S1A). There was a significant sex- and ethnicity-adjusted enrichment in pLOF *LY9* variants among TB patients (*N*=1,733) relative to controls without mycobacterial infection (*N*=21,274) (adjusted OR [95% CI] = 81.7 [7.9 – 10986.5], *P* = 0.00012) (Table S1; Methods). Two Moroccan patients (P1 and P3) had a biallelic frameshift variant (c.182del, G61Vfs*3), whereas a Turkish patient (P4) was homozygous for a different frameshift variant (c.1052_1053del, H351Rfs*22) (Fig. 1D and E, fig. S1B, Table S2 and 3). Genotyping of P1’s family revealed that the paternal uncle of P1 (P2) — who was 28 years old and had been vaccinated with BCG without experiencing any unusually severe infections, including TB or BCG disease — was also homozygous for the c.182del allele. For P2, an interferon-gamma release assay (IGRA; Quantiferon-TB Gold Plus) was negative, and the chest X ray was clear (Fig. 1F), indicating an absence of prior *M.tb* infection. Sanger sequencing validated these variants (fig. S1C). By contrast, biallelic pLOF LY9 genotypes are rare in the general population (gnomAD), with an estimated frequency of biallelic pLOF genotypes at 3.6 × 10^−6^ (Fig. 1G and fig. S1D). *LY9* encodes LY9 (also known as SLAMF3 or CD229), a transmembrane glycoprotein expressed at high levels on T and B lymphocytes and a low level on NK lymphocytes but non-detectable on monocytes, granulocytes, platelets, and red blood cells in humans (46) (Fig. 1H). The patients’ *LY9* variants (c.182del and c.1052_1053del) were predicted to cause truncation of LY9 protein in its extracellular domain (G61Vfs*3 and H351Rfs*22, respectively) (Fig. 1I). Therefore, we hypothesized that the three patients suffer from TB disease, rather than spontaneous clearance or long-term latency, due to AR complete LY9 deficiency that impairs LY9-dependent immunity to *M.tb*.

### Loss-of-function of the patients’ LY9 alleles in an overexpression system

**Figure 2.**
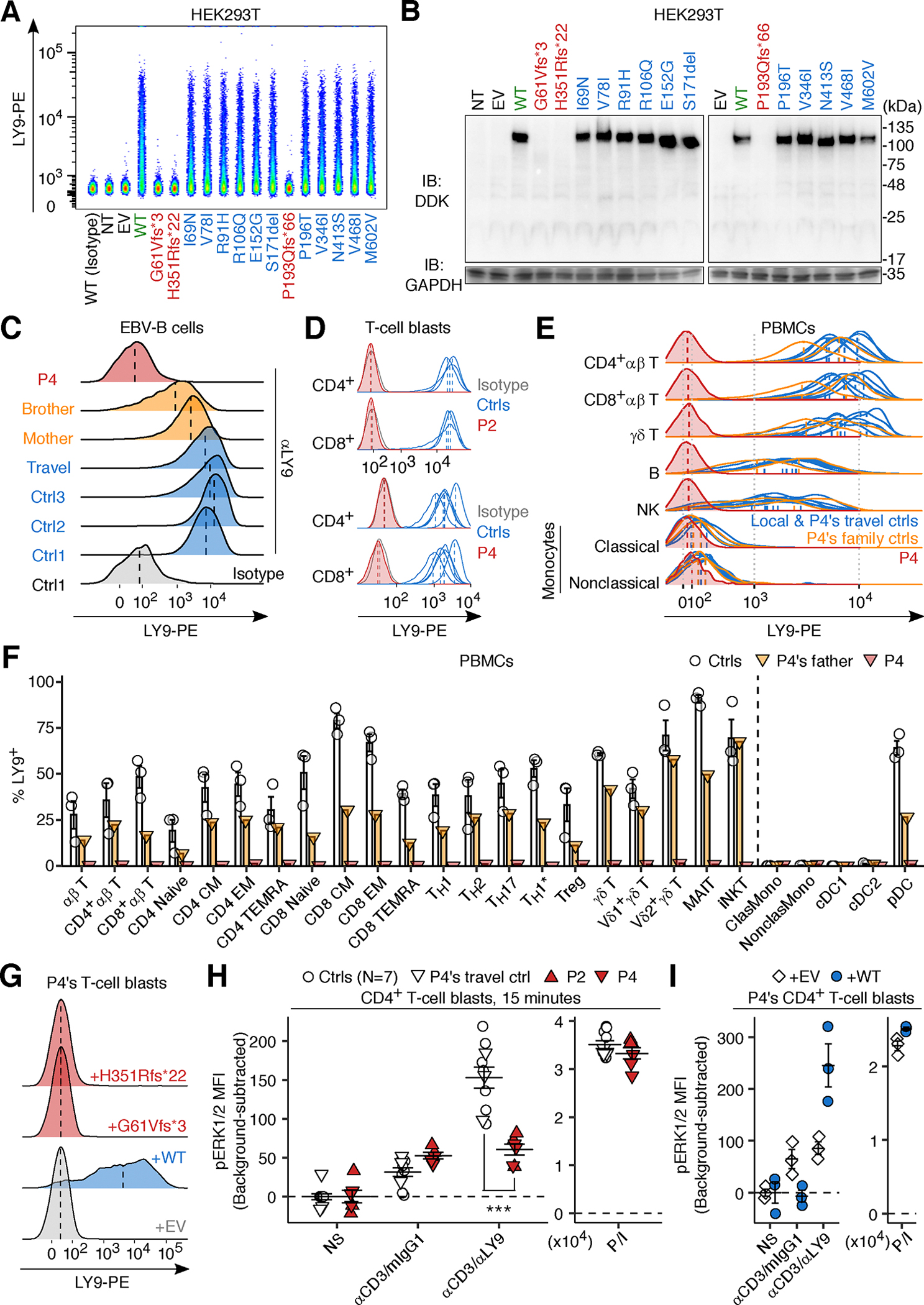
Analysis of LY9 expression and function. (A and B) Analysis of LY9 expression in an overexpression system. HEK293T cells were transfected with an empty vector (EV) or plasmids encoding the wild-type (WT) or mutant LY9 proteins with a C-terminal DDK tag. Representative results of two independent experiments. (A) Surface LY9 expression, as determined by flow cytometry with a PE-conjugated anti-LY9 mAb. (B) Immunoblotting of total protein extract probed with an anti-DDK monoclonal antibody (mAb). GAPDH was used as a loading control. (C-I) Analysis of LY9 expression and function in the patients’ cells. (C-F) Surface LY9 expression in (C) Epstein-Barr virus-immortalized B (EBV-B) cells, (D) T-cell blasts (T-blasts), and (E and F) peripheral blood mononuclear cell (PBMC) subsets, as determined by flow cytometry. (G) Rescue, by lentiviral transduction, of surface LY9 expression in T-blasts from P4. (H and I) ERK phosphorylation assay. (H) Non-transduced CD4^+^ T-blasts or (I) lentivirally transduced CD4^+^ T-blasts from P4 were stimulated for 15 minutes with bead-conjugated mAbs, and the levels of phospho-ERK1/2 were measured by flow cytometry. Technical triplicates were performed for cells from the patients and for P4’s travel control. In A-D and G-I, representative results from two independent experiments are shown. For αCD3/αLY9 stimulation in H, statistical significance was determined for the difference in background-subtracted median fluorescence intensity between all controls and LY9-deficient individuals. ***, *P* < 0.001 by unpaired Wilcoxon’s rank-sum test. In C-E and G, vertical dashed lines represent the median. In F, H, and I, bars represent the mean and SEM. P/I, phorbol 12-myristate 13-acetate (PMA) and ionomycin.

We investigated the expression of LY9 protein in HEK293T cells transfected with cDNAs corresponding to WT *LY9* or the patients’ variants. When overexpressing cDNAs corresponding to the patients’ variants, no LY9 protein was detected at the cell surface by flow cytometry (Fig. 2A). We also excluded the possibility of re-initiation of translation by immunoblotting using LY9 constructs containing a C-terminal DDK tag inserted upstream from the newly generated stop codon resulting from the frameshift variants (Fig. 2B). When using LY9 constructs containing a C-terminal DDK tag inserted upstream from the newly generated stop codon resulting from the frameshift variants, we detected a truncated protein for H351Rfs*22, but not for G61Vfs*3 (fig. S2A). We then made *LY9-*knockout (KO) HuT78 T-lymphoma cells transduced with cDNAs corresponding to WT *LY9* or the patients’ variants. We confirmed the abscence of cell-surface LY9 protein by flow cytometry (fig. S2B). We designed LY9 crosslinking assay using magnetic beads conjugated with anti-LY9 or isotype control (mouse IgG1) monoclonal antibody (mAb), together with bead-immobilized anti-CD3 mAb (fig. S2C). Parental cells or cells treated with negative control (NC) sgRNA showed anti-LY9 mAb-mediated enhancement in anti-CD3 mAb-induced TNF production, whereas cells with a knockout of *LY9* did not (fig. S2D and E). Transduction with a cDNA encoding the WT LY9 rescued the anti-LY9 mAb-mediated enhancement of TNF production, whereas the two patients’ variants were LOF (fig. S2D and E). Finally, only three out of 12 non-synonymous variants found in the homozygous state in the in-house or gnomAD database (Methods) were either hypomorphic (E152G and V346I) or LOF (P193Qfs*66) (Fig. 2A, 2B, and fig. S2B and S2E). The cumulative frequency of experimentally validated hypomorphic or LOF alleles (2.0 × 10^−3^) was almost identical to that of pLOF alleles (1.9 × 10^−3^). Thus, the patients’ alleles were loss-of-expression and LOF in an overexpression system.

### Inherited complete LY9 deficiency in the three unrelated TB patients

We then investigated the expression and function of the LY9 protein in the patients’ cells. Flow cytometry with an anti-LY9 mAb detected no LY9 protein on the surface of Epstein-Barr virus-immortalized B (EBV-B) cells from P4, despite the presence of normal levels of *LY9* mRNA, and on expanded T-cell blasts (T-blasts) from P2 and P4, whereas LY9 protein was detected on the surface of the corresponding cells from healthy controls, including P4’s travel control (a healthy control of the same ethnicity as P4 whose blood sample was transported to our laboratory together with those collected from P4) (Fig. 2C–D and S3A–C). Flow cytometry also revealed the presence of high levels of LY9 on T (αβ T, Vδ1^+^ and Vδ2^+^ γδ T, MAIT, and iNKT) lymphocytes and plasmacytoid dendritic cells (pDCs), and low levels on B and NK lymphocytes and innate lymphoid cells (group 2 and progenitors; ILC2 and ILCPs) (Fig. 2E–F and S3D). The selective expression of LY9 on the pDC subset of myeloid leukocytes is consistent with the expression of a range of lymphocytic surface molecules on pDCs (47). By contrast, LY9 was barely detectable on classical and non-classical monocytes and the two types of conventional dendritic cells (cDCs) among peripheral blood mononuclear cells (PBMCs) and macrophages derived from the blood monocytes of healthy controls (Fig. 2E–F and S3D–E). Lentiviral transduction with WT LY9, but not with empty vector (EV) or two mutants (G61Vfs*3 or H351Rfs*22) rescued the surface expression of LY9 on T-blasts from P4, as shown by flow cytometry (Fig. 2G and S3F). Finally, CD4^+^ T-blasts from P2 and P4 displayed lower levels of ERK1/2 phosphorylation following mAb-mediated LY9 crosslinking than cells from local healthy donors or P4’s travel control (Fig. 2H). This phenotype was rescued by lentiviral transduction with WT LY9 (Fig. 2I). Thus, the four homozygous individuals from three kindreds, including the three patients with TB, have AR complete LY9 deficiency.

### Impaired M.tb-induced IFN-γ-driven responses in LY9-deficient leukocytes

**Figure 3.**
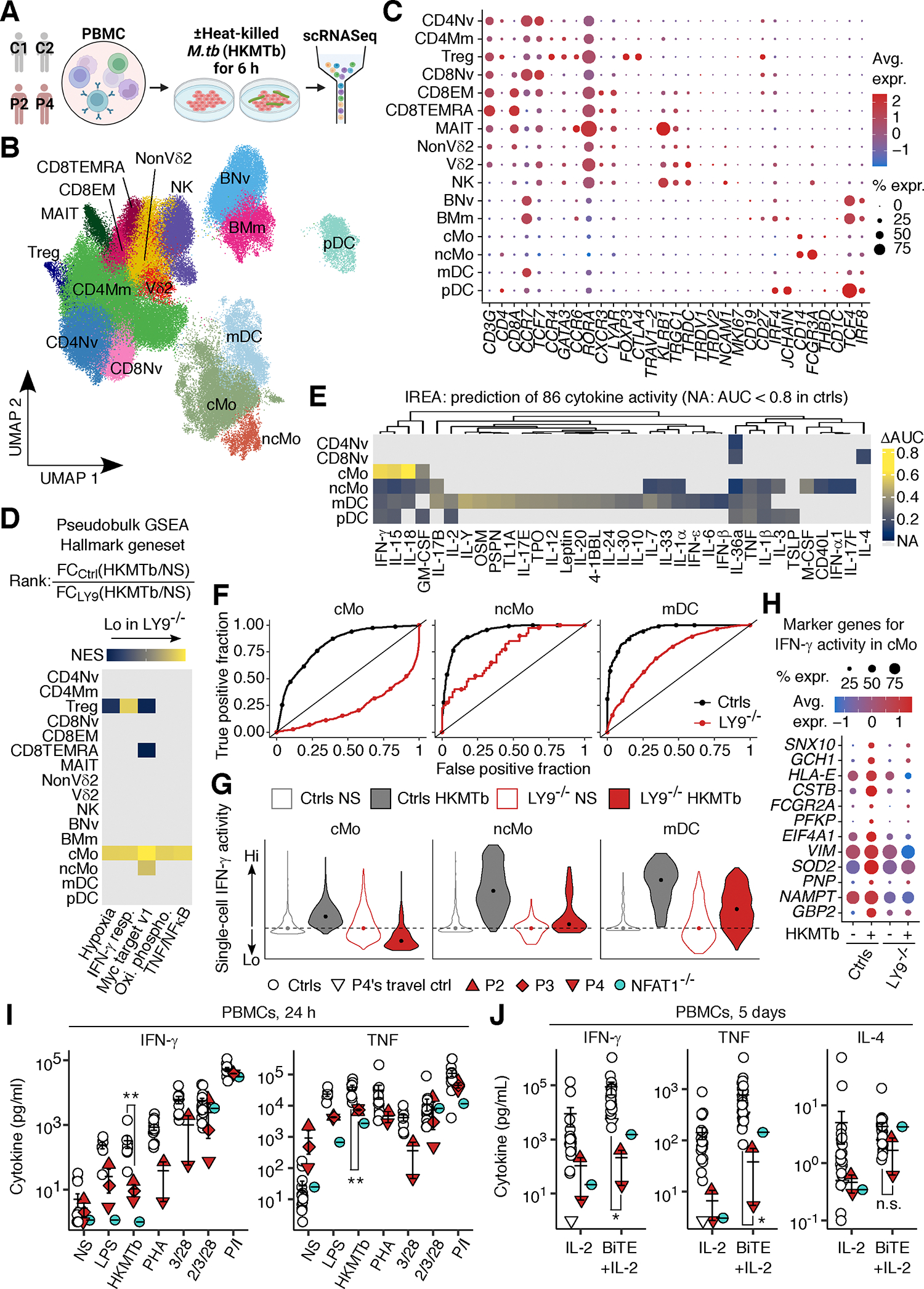
Impaired IFN-γ-driven leukocytic responses to *M.tb*. (A-H) Single-cell RNA sequencing (scRNASeq) analysis. PBMCs from P2 and P4 (aged 29 and 17 years, respectively; P2 had had no unusually severe infections; P4 was in complete remission from TB and off all treatment) were either left unstimulated or were stimulated with heat-killed *M.tb* (HKMTb) for 6 hours and then subjected to scRNASeq. (A) Experimental design. (B) Clustering and cell-type annotation. (C) Marker gene expression. (D) Geneset enrichment analysis (GSEA). Genesets with FDR-adjusted *P* values < 0.05 in any of the leukocyte subsets tested in the LY9-deficient individuals relative to controls are shown. (E) Immune response enrichment analysis (IREA) (48). The activity of 86 cytokines was predicted in each leukocyte subset (Method). (F) ROC curves for IFN-γ activity. (G) Distributions of IFN-γ activity. The dots in the violin plot represent the mean values. (H) Expression levels of genes contributing to the predicted IFN-γ activity (mean log_2_FC > 0 and FDR-adjusted *P* value < 0.05) in classical monocytes. Only genes with a percentage of expression > 20% and a scaled mean expression > 1 in HKMTb-stimulated control cells are shown. (I) PBMC stimulation assay. PBMCs from P2, P3, and P4 (aged 29, 40, and 17 years, respectively), one NFAT1-deficient patient (50), and healthy controls were stimulated with the indicated reagents for 24 hours. Results from three experiments were compiled, with all technical replicates averaged. Some conditions were omitted for P3’s and NFAT-deficient cells due to limited sample availability. (J) Autologous T and B lymphocyte engagement assay. PBMCs from P2, P4 (aged 29 and 17, respectively), one NFAT1-deficient patient, and healthy controls were stimulated with blinatumomab (a bispecific antibody targeting CD3 and CD19 to induce immune synapses between autologous T and B lymphocytes) for 5 days. Results from eight experiments are compiled, with all technical replicates averaged. In I and J, bars represent the mean and SEM. Statistical significance was determined for the difference between all controls and LY9-deficient individuals. n.s., not significant; *, *P* < 0.05; **, *P* < 0.01 by unpaired Wilcoxon’s rank-sum tests. LPS, lipopolysaccharides; HKMTb, heat-killed *M.tb* lysate; PHA, phytohemagglutinin; P/I, PMA and ionomycin; BiTE, blinatumomab.

We did not uncover any shared abnormalities in any of the lymphoid and myeloid leukocyte subsets during our initial attempt to agnostically delineate the impact of inherited LY9 deficiency on leukocyte subsets through immunophenotyping and single-cell RNA sequencing (scRNASeq) analysis of PBMCs from P2, P3, and P4 (sampled at the ages of 29, 40, and 15 or 16 years, respectively) (fig. S4A–D). Likewise, we did not find abnormalities in serum antibody (Ab) profiles in P4 (age 15 years), or B cell immunophenotypes and *in vitro* antibody production in P3 (aged 40 years) (fig. S5A–G and S6A–D). As our genetic findings suggest a strong selective association between LY9 deficiency and TB, we hypothesized that responses of LY9-deficient leukocytes to *M.tb* are impaired. We profiled the transcriptomic responses of PBMCs from P2 (sampled at the age of 29 years), P4 (17 years), and healthy controls to heat-killed *M.tb* (HKMTb) via scRNASeq (Fig. 3A). Clustering analysis identified 17 leukocyte subsets (Fig. 3B and C). Geneset enrichment analysis (GSEA) and immune response enrichment analysis (IREA) (48) identified a strikingly impaired and cell-type-selective impairment in the HKMTb-triggered transcriptional responses to IFN-γ, such as *GBP2*, *NAMPT*, and *SOD2,* in LY9-deficient classical monocytes, relative to cells from healthy donors (Fig. 3D–H). LY9-deficient non-classical monocytes and mDCs showed a similar but less pronounced phenotype (Fig. 3E–G). These findings suggest that LY9 deficiency does not abolish the development of any lymphoid and myeloid leukocyte subsets but functionally impairs IFN-γ-driven leukocytic responses to *M.tb*.

### Impaired secretion of IFN-γ and TNF by LY9-deficient leukocytes

IFN-γ is indispensable for antimycobacterial immunity in humans (14, 18), and even partial impairments of IFN-γ production predispose humans to TB (27, 28, 31, 32, 49). We assessed the secretion of IFN-γ and other cytokines by PBMCs from P2, P3, and P4 (sampled at the ages of 29, 40, and 17 years, respectively). LY9-deficient PBMCs secreted significantly (*P*=0.009) smaller amounts of IFN-γ in response to HKMTb (Fig. 3I). The secretion of IFN-γ induced by phytohemagglutinin (PHA) and of TNF by HKMTb, lipopolysaccharides (LPS), and bead-conjugated anti-CD3/28 mAbs was also impaired, whereas responses to a cocktail of anti-CD2/3/28 mAbs or PMA and ionomycin (P/I) were unaffected (Fig. 3I). PBMCs from an NFAT1-deficient patient (50) had similar phenotypes (Fig. 3I). By contrast, the secretion of IL-17A, IL-17F, and IL-22 was largely unaffected (fig. S7A). We also found that PBMCs from P2 and P4 secreted significantly less IFN-γ and TNF (*P*=0.018 for both) after stimulation with blinatumomab (a bispecific antibody targeting CD3 and CD19 to form an artificial immunological synapse between autologous T and B lymphocytes), whereas IL-4, IL-17A, IL-17F, and IL-22 were secreted normally (Fig. 3J and S7B). By contrast, the secretion of IFN-γ, TNF, IL-1β, IL-6, and IL-10 by P4’s PBMCs (aged 15 years) in response to IL-12 or IL-23 was normal (fig. S7C). Likewise, the secretion of IL-17A and ISG15 by PBMCs from P3 and P4 (aged 40 and 17 years, respectively) in response to P/I was also normal (fig. S7C and D). Thus, LY9 deficiency impairs the production of IFN-γ and TNF by leukocytes in response to *M.tb* and other stimuli.

### Impaired production of IFN-γ by LY9-deficient T_H_1* cells in response to mycobacteria

**Figure 4.**
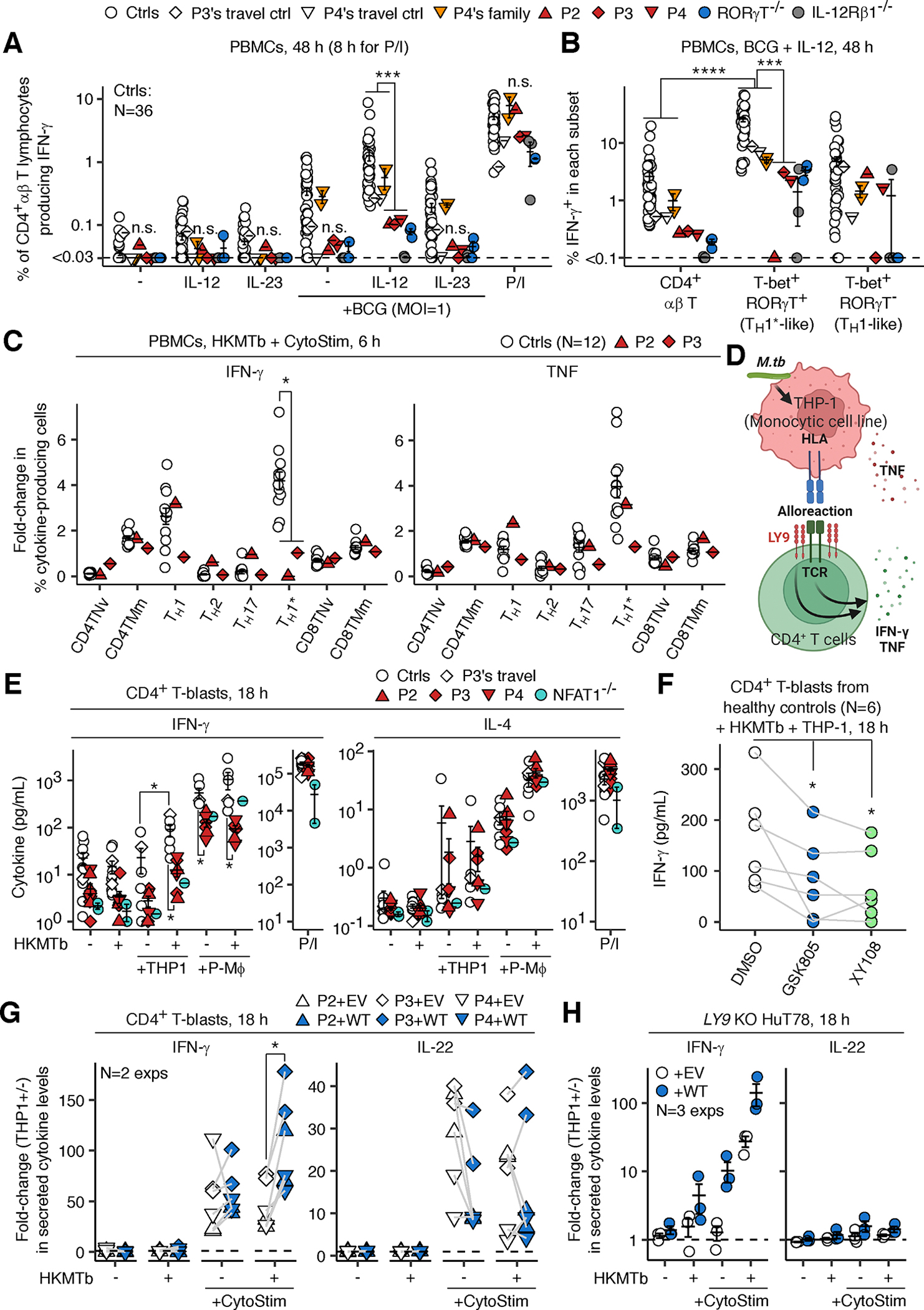
Impaired IFN-γ production by LY9-deficient T_H_1* cells. (A and B) BCG assay. PBMCs from P2, P3, and P4 (aged 29, 40, and 15 years, respectively; P2 had no unusually severe infections; P3 and P4 were in complete remission from TB and off all treatment), three IL-12Rβ1-deficient patients, three RORγT-deficient patients, and healthy donors were stimulated with live BCG mycobacteria with or without IL-12 or IL-23 for 48 hours. Secretion inhibitors were added for the last 8 hours, and IFN-γ production was quantified by flow cytometry. Results from four experiments were compiled, with P2, P3, and P4 tested on different occasions. No technical replicates were prepared. (A) IFN-γ-producing CD4^+^ αβ T lymphocytes. (B) Enrichment of IFN-γ-producing CD4^+^ αβ T lymphocytes within the T-bet^+^RORγT^+^ compartment. (C) IFN-γ and TNF production by T_H_1, T_H_2, T_H_17, and T_H_1* cells among the PBMCs of P2 and P3 (aged 29 and 40 years, respectively) and controls, as measured by intracellular flow cytometry after 6 hours of stimulation with secretion inhibitors. CytoStim (a bispecific antibody for TCRβ and HLA) was used to enhance the T-cell response regardless of the antigenic specificity. Results from four experiments were compiled, with all technical replicates averaged. (D-G) THP-1:CD4^+^ T-cell coculture assay. (D) Schematic diagram. (E) MACS-enriched CD4^+^ T-blasts were cocultured with THP-1 monocytic leukemia cells (undifferentiated) or THP-1-derived macrophage-like cells differentiated with PMA for 48 hours (P-Mφ). Results from three experiments were compiled, with cells from P2, P3, and P4 tested twice, with all technical replicates averaged. (F) Pharmacological RORγT inhibition. CD4^+^ T-blasts from healthy donors were cocultured with THP-1 cells and HKMTb for 18 hours with two RORγT inhibitors (GSK805 and XY108). Results from two experiments with different healthy controls were compiled. (G) MACS-enriched CD4^+^ T-blasts from P2, P3, and P4 lentivirally transduced with empty vector (EV) or WT LY9 were cocultured with THP-1 cells and the reagents indicated. The fold-change in secreted cytokine levels was determined by dividing cytokine levels in the presence of THP-1 cells by those in the absence of THP-1 cells. (H) *LY9*-knockout (KO) HuT78 T-lymphoma cells lentivirally transduced with EV or WT LY9 were cocultured with THP-1 cells and the reagents indicated. The fold change in secreted cytokine levels was calculated as in F. In A-C, E, and H, bars represent the mean and SEM. In A-C and E-G, n.s., not significant; *, *P* < 0.05; **, *P* < 0.01; ***, *P* < 0.001; ****, *P* < 1 × 10^−4^ by unpaired Wilcoxon’s rank-sum tests (A, the comparison between all controls and LY9-deficient individuals in B, C, and the comparisons between all controls and LY9-deficient individuals in E) or paired Wilcoxon signed-rank tests (the comparison of different subsets in controls in B, the comparison of different conditions in controls in E, F, and G). HKMTb, heat-killed *M.tb* lysate. P/I, PMA and ionomycin.

We investigated IFN-γ production by lymphocyte subsets (CD4^+^ and CD8^+^ αβ T, MAIT, iNKT, Vδ1^+^ γδ T, Vδ2^+^ γδ T, NK, and B lymphocytes) in PBMCs from P2, P3, and P4 (sampled at the ages of 29, 40, and 15 years, respectively; all BCG-vaccinated) by flow cytometry. Upon stimulation with live BCG plus IL-12, CD4^+^ αβ T lymphocytes from LY9-, RORγT-, and IL-12Rβ1-deficient individuals (*N*=3 each) showed impaired IFN-γ production, especially within the T-bet^+^RORγT^+^ (a proxy of T_H_1* cells) compartment (Fig. 4A, B, and S8A–C). By contrast, LY9 deficiency did not impair IFN-γ production by non-CD4^+^ αβ T lymphocytes nor TNF production by all lymphocyte subsets tested (fig. S8C and S8D). As the intracellular domain of LY9 interacts with signaling lymphocytic activation molecule (SLAM)-associated protein (SAP) (51), we tested whether SAP deficiency shares a similar phenotype. However, SAP deficiency (N=3) did not impair CD4^+^ αβ T-cell IFN-γ production (fig. S8E). We studied cytokine production by T helper subsets by flow cytometry (fig. S9A and B). Upon stimulation with HKMTb plus CytoStim (a bispecific antibody to TCRβ and HLA-I/II; to study phenotypes of αβ T cells regardless of antigen specificity), T_H_1* cells from healthy controls showed the highest levels of IFN-γ production, followed by T_H_1 cells, whereas T_H_2 and T_H_17 cells barely produced IFN-γ (Fig. 4C, S9A, and S9B). In PBMCs of P2 and P3 (sampled at the ages of 29 and 40 years), LY9-deficient T_H_1* cells showed impaired IFN-γ production but normal production of TNF (Fig. 4C). By contrast, LY9 deficiency did not impair IFN-γ or TNF production triggered by HKMTb alone or P/I by any leukocyte subsets studied (fig. S9C and D). Thus, LY9 deficiency impairs the production of IFN-γ by T_H_1* cells in TCR- and *Mycobacterium*-dependent contexts.

### LY9-mediated cell-intrinsic enhancement of CD4^+^ T-cell IFN-γ production

The defective production of IFN-γ by LY9-deficient CD4^+^ αβ T lymphocytes upon stimulation with HKMTb and CytoStim, the latter acting as a superantigen, cannot be attributed to cognate antigen-specific mechanisms. We hypothesized that LY9 on T lymphocytes acts as a costimulatory receptor once translocated to an immunological synapse upon engagement with antigen-presenting cells (APCs) (52). We tested this scenario in an allogeneic coculture of CD4^+^ T-blasts from P2, P3, P4, or healthy donors with THP-1 cells (a monocytic leukemia cell line) or PMA-differentiated THP-1-derived macrophages (P-Mφ), with or without HKMTb (Fig. 4D). CD4^+^ T-blasts from healthy donors secreted significantly (*P*=0.03) more IFN-γ when cocultured with THP-1 plus HKMTb than with THP-1 alone, which was suppressed by two RORγT inhibitors (GSK805 and XY108) and a cell-permeable NFAT inhibitor (11R-VIVIT) (Fig. 4E, F, and S10A-C). LY9-deficient CD4^+^ T-blasts showed significantly less IFN-γ secretion than control cells upon coculture with THP-1 plus HKMTb, P-Mφ, or HKMTb plus P-Mφ (*P*=0.02, 0.01, and 0.01, respectively), while normally secreting IL-4 (Fig. 4E). Raji B lymphoma cells with or without *LY9* KO as cocultured APCs yielded similar results (fig. S10D), suggesting a T-cell-intrinsic role of LY9 in this phenotype. Indeed, complementation with WT LY9 via lentiviral transduction, unlike empty vector (EV), was sufficient to promote IFN-γ secretion induced by THP-1 plus HKMTb plus CytoStim in LY9-deficient CD4^+^ T-blasts or *LY9* KO HuT78 T-lymphoma cells (Fig. 4G). The secretion of TNF was also rescued, albeit to a lesser extent than IFN-γ, whereas IL-4 and IL-22 were largely unaffected (Fig. 4G, H, and S10E). Thus, consistent with the phenotypes of primary T_H_1* cells, LY9 operates in a CD4^+^ T lymphocyte-intrinsic fashion to promote IFN-γ production in the presence of *M.tb* and APCs.

### *Impaired IFN-γ production by LY9-deficient* Mycobacterium*-specific CD4*^*+*^ *αβ T cells*

We hypothesized that LY9-deficient *Mycobacterium*-specific CD4^+^ αβ T lymphocytes would have impaired IFN-γ-producing capacity, as *Mycobacterium*-specific CD4^+^ αβ memory T cells are known to be enriched within the CCR4^−^CCR6^+^CXCR3^+^ T_H_1* compartment, the function of which depends on RORγT in humans (43, 44). We therefore investigated IFN-γ production by *M.tb*-specific CD4^+^ αβ T-cell clones isolated from P4’s PBMCs (sampled at the age of 17 years) and expanded for 43 days *in vitro* (fig. S11A). P4’s *M.tb*-specific CD4^+^ αβ T-cell clones displayed impaired production of IFN-γ and, less strikingly, of TNF, upon stimulation with plate-bound anti-CD3 mAb or PHA. By contrast, IL-4 production was within a normal range (fig. S11B and C), consistent with the defective IFN-γ production of T_H_1* cells from P2 and P3 (Fig. 4C). Interestingly, P4’s *C. albicans*-specific CD4^+^ αβ T-cell clones presented no impairment of IFN-γ, TNF, or IL-4 production (fig. S11C). We then re-expanded *M.tb*-specific CD4^+^ αβ T-cell clones from healthy donors and P4 for two weeks for further investigations. Re-expanded *M.tb*-specific clones from P4 displayed an impaired IFN-γ secretion in a ProQuantum qPCR-ELISA assay (fig. S11D). Moreover, RNASeq revealed that *IFNG* was among the genes most strongly downregulated in P4’s *M.tb*-specific clones relative to those from healthy controls (bottom 1.5% and 0.4% in the non-stimulated and TCR-stimulated states, respectively) (fig. S11E and F). By contrast, P4’s *M.tb*-specific clones expressed *IL2, IL4, IL13,* and *IL17A* at levels similar to or higher than those in control cells (fig. S11E and F). Thus, inherited LY9 deficiency impairs IFN-γ production by *Mycobacterium*-specific CD4^+^ αβ T lymphocytes.

### Impaired capacity of LY9-deficient CD4^+^ T cells to help phagocytes restrict intra-macrophagic bacterial growth

Our findings demonstrate an impairment of IFN-γ production by LY9-deficient primary and cultured CD4^+^ T cells, specifically mycobacterium-specific T_H_1* cells. IFN-γ has been shown to restrict the growth of *M.tb* and other intramacrophagic pathogens in human phagocytes (38, 53–55). We investigated whether the capacity of LY9-deficient CD4^+^ T lymphocytes to help phagocytes suppress the growth of intramacrophagic pathogens in response to stimulation with HKMTb or P/I was impaired. THP-1 monocytic leukemia cells infected with GFP-expressing *Listeria monocytogenes* (as a probe for intramacrophagic microbial growth) were cocultured with CD4^+^ T-blasts from P2, P3, P4, or healthy controls (fig. S12A–C). The viability of the THP-1 cells and the proportions of GFP^+^ THP-1 cells were similar between cocultures of THP-1 cells with control and LY9-deficient CD4^+^ T-blasts (fig. S12D and E). We calculated the total GFP fluorescence intensity in GFP^+^ THP-1 cells as a proxy for the total intramacrophagic microbial burden. THP-1 cells cocultured with LY9-deficient CD4^+^ T-blasts from P2, P3, and P4 had higher total GFP fluorescence levels than THP-1 cells cocultured with control CD4^+^ T-blasts in the presence of HKMTb (fig. S12F and G). By contrast, P/I decreased GFP fluorescence similarly in THP-1 cells cocultured with control cells and those cocultured with LY9-deficient CD4^+^ T-blasts, resulting in total GFP fluorescence levels much lower than those observed after stimulation with HKMTb (fig. S12F and G). Thus, as predicted from their impaired IFN-γ production, LY9-deficient CD4^+^ T lymphocytes also have an impaired capacity to help phagocytes restrict intramacrophagic pathogens.

### LY9-dependent acquisition of effector functions in T_H_1* cells

We investigated the roles of LY9 in the development and functional maturation of T_H_1* cells. We found that 1) in healthy donors, T_H_1* cells had the highest levels of LY9 protein among CD4^+^ αβ T-cell subsets, followed by T_H_17, T_H_2, T_H_1, Treg, and naïve CD4^+^ αβ T cells; and 2) CD4^+^ T-blasts from two RORγT-deficient patients (43) had lower levels of LY9 expression than cells from healthy donors or a T-bet-deficient patient, suggesting that RORγT increases LY9 expression in CD4^+^ T lymphocytes (fig. S13A–D). By analyzing the transcriptional signatures of T_H_1* cells at baseline of P2, P3, and P4 (described in fig. S4B–D), we observed that 1) LY9-deficient T_H_1* cells had more differentially expressed genes (DEGs vs. control cells) than other CD4^+^ T-lymphocyte subsets (i.e., naïve, T_H_1/2/17, and Tregs) (fig. S14A and B); 2) LY9-deficient T_H_1* cells had a significantly (FDR-adjusted *P* < 10^−7^) reduced expression of genesets related to cell-cell adhesion and immunological synapse formation, such as *ITGA4*, *ITGB1*, and *ICAM3* (encoding the integrin α4 and β1 subunits and ICAM-3) (fig. S14C and D); 3) these immune synapse-related genes were more abundantly expressed in CD4^+^ memory than in naïve T lymphocytes, which were selectively downregulated in T_H_1* cells in the absence of LY9 (fig. S14E); and 4) LY9-deficient T_H_1* cells showed genes driven by a T_H_2/T_H_17-promoting transcription factor ATF6 (56) (fig. S14F). These findings suggest that LY9 is upregulated during differentiation from naïve CD4^+^ αβ T cells to T_H_1* cells and is critical for differentiating precursors to acquire T_H_1*-specific effector functions.

### T_H_1*-oriented memory differentiation of naive CD4^+^ αβ T lymphocytes via LY9

**Figure 5.**
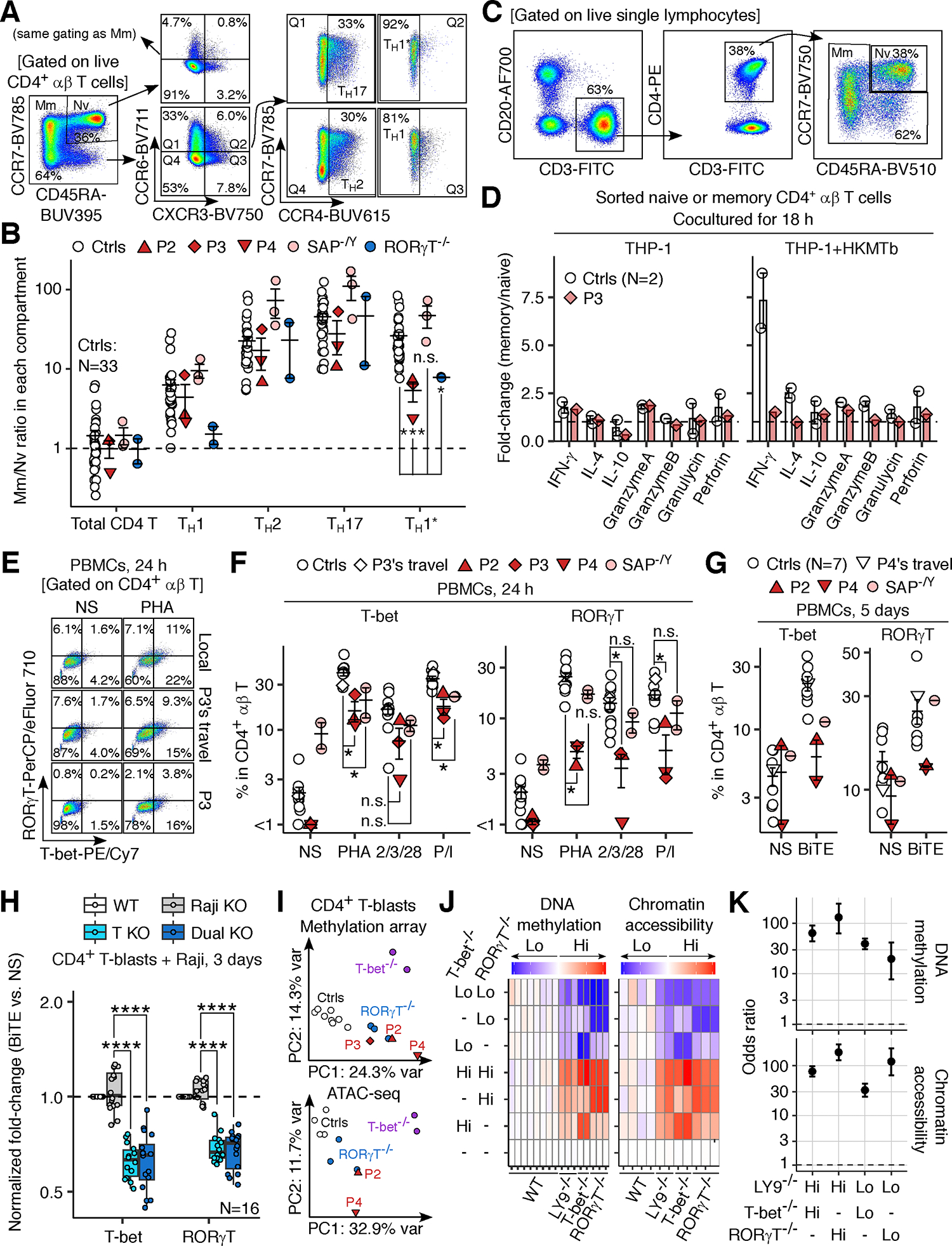
LY9 polarizes TCR-primed CD4^+^ αβ T lymphocytes to differentiate into T_H_1* cells by promoting the expression of T-bet and RORγT. (A) Gating strategy for T_H_ cells and T_H_-like naive CD4^+^ αβ T lymphocytes. (B) The number of canonical T_H_ cells divided by the number of T_H_-like naïve CD4^+^ αβ T lymphocytes. The total memory/naive CD4^+^ T-cell ratio is also shown. (C and D) Naïve and memory CD4^+^ T lymphocytes sorted from the PBMCs of P3 (aged 40 years) and two healthy donors were cocultured with THP-1 or THP-1 plus HKMTb for 18 hours. (C) Sorting strategy. (D) The memory-to-naïve fold-change to assess the acquisition of cytokine-producing capacity in memory CD4^+^ T lymphocytes relative to their naïve counterparts from the same individual. The dashed horizontal line represents 1. (E and F) PBMCs from P2, P3, and P4 (aged 29, 40, and 17 years), P3’s travel control, and healthy controls were stimulated for 24 hours. (E) Representative plots for T-bet and RORγT in P3’s CD4^+^ αβ T lymphocytes. (F) The expression of T-bet and RORγT in CD4^+^ αβ T lymphocytes was quantified by flow cytometry. Results from three experiments were compiled, with all technical replicates averaged. (G) PBMCs from P2 and P4 (aged 29 and 17 years) and healthy controls were stimulated for 5 days with blinatumomab (anti-CD3-CD19 bispecific T-cell engager; BiTE). The expression of T-bet and RORγT in CD4^+^ αβ T lymphocytes was quantified by flow cytometry. Results from three experiments were compiled, with all technical replicates averaged. (H) Coculture assay with CD4^+^ T-blasts from two healthy donors and Raji cells with or without *LY9* KO. The fold-change difference in MFI between non-stimulated and BiTE-stimulated conditions was calculated, with further normalization based on the data obtained without KO in either of the cell types cultured together. Four technical replicates were prepared for each of the CD4^+^ T-cell donors. Results from two experiments were compiled. (I-K) DNA methylation microarray analysis [healthy donors (*N*=8), P2, P3, and P4] and Omni-ATAC-seq analysis [healthy donors (*N*=4), P2, and P4] of MACS-enriched CD4^+^ T-blasts. Cells from one T-bet-deficient patient (T-blasts prepared on two different occasions as technical duplicates) and three RORγT-deficient patients were compared. See Methods for additional details. (I) Principal component analysis (PCA). (J) Heatmap analysis of CpG sites or chromatin regions governed by T-bet, RORγT, or both. (K) Overlap between LY9-dependent and T-bet- or RORγT-dependent sites or regions. In B, F, and G, bars represent the mean and SEM. In B, F, and H, n.s., not significant; *, *P* < 0.05; **, *P* < 0.01; ***, *P* < 0.001; ****, *P* < 1 × 10^−4^ by unpaired Wilcoxon’s rank-sum tests. HKMTb, heat-killed *M.tb* lysate; PHA, phytohemagglutinin; P/I, PMA and ionomycin; BiTE, blinatumomab.

We hypothesized that LY9 deficiency reduces the ability of naïve CD4^+^ αβ T lymphocytes primed by antigen-presenting DCs to differentiate into T_H_1* cells (57). Human CD45RA^+^CCR7^+^ naïve CD4^+^ T lymphocytes contain heterogenous populations (including stem-cell memory and lineage-committed T_H_ precursors) expressing CXCR3 or CCR6 and capable of producing IFN-γ or IL-17 (58–64). Extending these observations, we enumerated the authentic T_H_1* cells and “T_H_1*-like naïve” (CCR4^−^CXCR3^+^CCR6^+^CD45RA^+^CCR7^+^) CD4^+^ αβ T cells for each donor, using the immunophenotyping data (described in fig. S4A). Healthy donors had about 30 times more T_H_1* cells than T_H_1*-like naïve CD4^+^ αβ T cells (Fig. 5A, 5B, and S15A). P2, P3, P4, and two RORγT-deficient patients had a significantly lower memory-to-naïve ratio in the T_H_1* compartment (CCR4^−^CXCR3^+^CCR6^+^) (*P*=8 × 10^−4^ and 0.02 for LY9 and RORγT deficiency, respectively) (Fig. 5B). By contrast, neither LY9 or RORγT deficiency affected the memory-to-naïve ratio in other T_H_ compartments (T_H_1/2/17), and three SAP-deficient patients displayed no abnormalities in any T_H_ compartments (Fig. 5B). Consistently, sort-purified memory CD4^+^ T cells from two healthy donors produced about seven times as much IFN-γ as their naïve counterparts when cocultured with THP-1 cells and HKMTb, whereas P3’s cells displayed no such enhancement (Fig. 5D). Coculture with THP-1 cells alone and stimulation with P/I did not increase the memory-to-naïve ratio for IFN-γ production for either control or P3’s cells (Fig. 5D and S15B). Similarly, memory CD4^+^ T cells from two healthy donors cultured with anti-CD2/3/28 mAb-conjugated beads plus IL-2 for 12 days produced about 10 times larger amounts of IFN-γ than their naïve counterparts, whereas P4’s cells displayed enhanced production of IL-5 and IL-13 instead of IFN-γ (fig. S15C and D). These findings suggest that LY9 promotes the differentiation of primed naive CD4^+^ αβ T lymphocytes into mature (IFN-γ-producing) T_H_1* cells.

### Enhanced T-bet and RORγT expression via LY9 on TCR-primed CD4^+^ αβ T lymphocytes

We hypothesized that LY9 directs the fate of TCR-primed naïve CD4^+^ αβ T lymphocytes toward fully functional T_H_1* cells by driving the expression of the necessary transcription factors. CD4^+^ αβ T lymphocytes in PBMCs from P2, P3, and P4 (sampled at the ages of 29, 40, and 17 years, respectively) showed lower levels of RORγT expression when stimulated with PHA, anti-CD2/3/28 mAbs, or P/I and lower levels of T-bet expression when stimulated with PHA or P/I (Fig. 5E and F). Interestingly, SAP-deficient CD4^+^ αβ T lymphocytes (N=2) showed normal levels of RORγT expression while showing lower levels of T-bet expression (Fig. 5F). Blinatumomab (anti-CD3-CD19 bispecific T-cell engager)-mediated engagement of autologous CD4^+^ αβ T and B lymphocytes resulted in similar defects in PBMCs from P2 and P4 (sampled at the ages of 29 and 17 years, respectively) and one SAP-deficient patient (Fig. 5G). Isogenic experiments showed that *LY9* KO in CD4^+^ αβ T lymphocytes and HuT78 T-lymphoma cells impaired the upregulation of T-bet and RORγT induced by polyclonal stimuli or coculture with Raji B lymphoma cells (with or without *LY9* KO) plus blinatumomab (Fig. 5H, S16A–D). Finally, DNA methylation and ATAC-seq analysis showed striking similarities among LY9-, T-bet-, and RORγT-deficient CD4^+^ T-blasts at the epigenetic level (Fig. 5I–K). Additional characterization of the expression of lineage-defining transcription factors (T-bet, GATA3, RORγT), their correlation with IFN-γ production, and the detailed documentation of epigenetic analyses can be found in the supplementary methods (fig. S16E–G and S17–S20). In summary, LY9 operates in a CD4^+^ αβ T-cell-intrinsic manner to promote the expression of T-bet and RORγT, leading to epigenetic polarization toward T_H_1* cells.

### Enhancement of IFN-γ production by T_H_1* cells by LY9 costimulation

**Figure 6.**
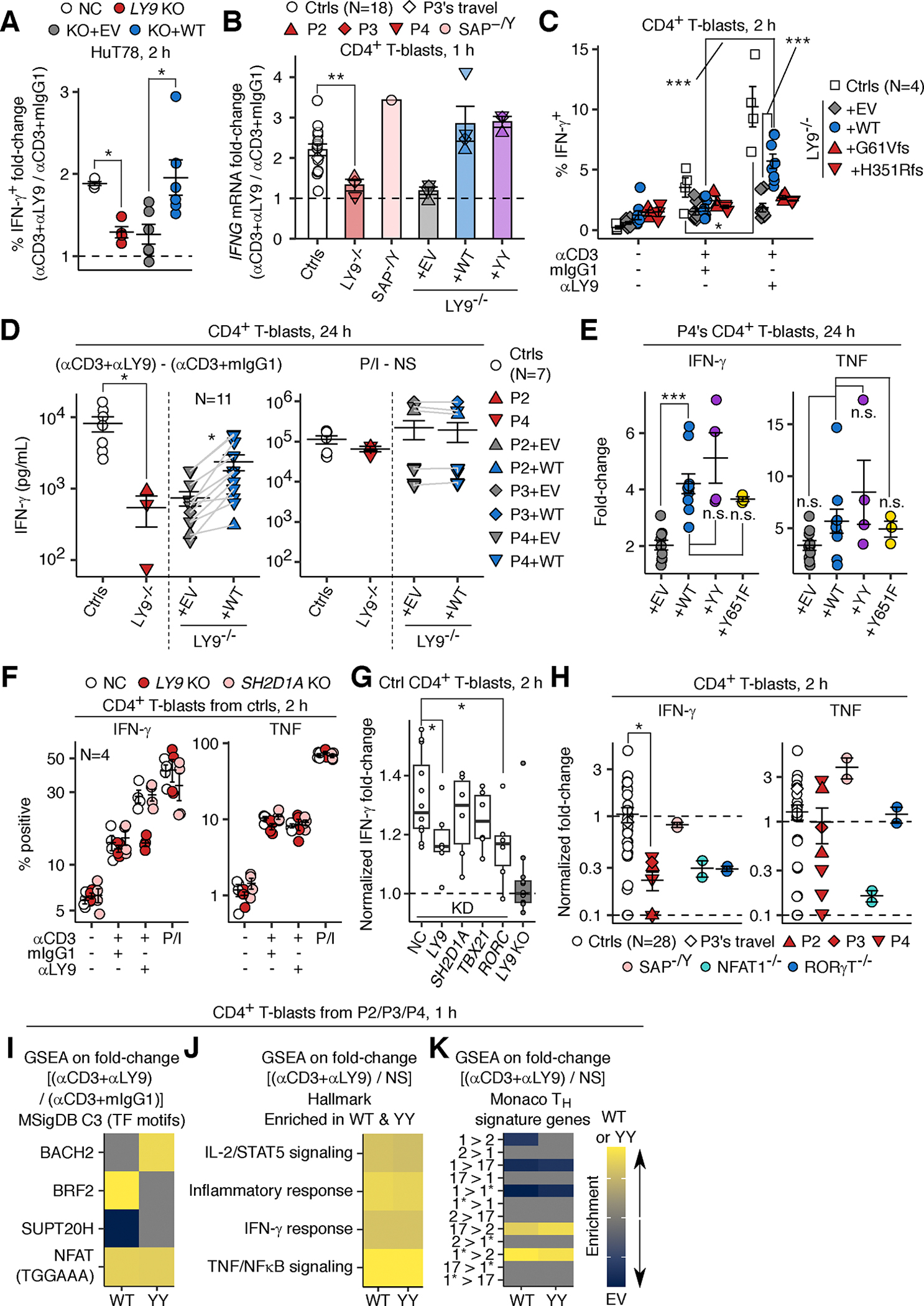
LY9 costimulation enhances CD4^+^ T-cell IFN-γ production via NFAT1 and RORγT. CD4^+^ T-blasts or HuT78 T-lymphoma cells were incubated with mock beads (beads without antibody) or beads conjugated with anti-CD3 and anti-LY9 mAbs or mIgG1 isotype control. P/I was used as a positive control. (A) IFN-γ production by HuT78 cells with or without *LY9* KO and transduced with EV or a lentivirus carrying the WT LY9 sequence, as determined by flow cytometry. Four and six technical replicates were prepared for non-transduced and transduced cells, respectively. Representative results from two experiments. (B) *IFNG* mRNA levels in CD4^+^ T-blasts from controls, P2, P3, P4, and one SAP-deficient patient, as determined by RT-qPCR. Similarly, CD4^+^ T-blasts from P2, P3, and P4 lentivirally transduced with EV, WT LY9, or LY9 with Y603A/Y626A substitutions (abbreviated YY) were analyzed. Results from five experiments were compiled, with all technical replicates averaged. (C) IFN-γ production by CD4^+^ T-blasts from controls and from P4 lentivirally transduced with EV, WT LY9, or mutant LY9, as determined by flow cytometry. Results from three experiments were compiled. Technical replicates were prepared for P4’s transduced cells (*N*=8 for EV and WT; *N*=6 for G61Vfs*3 and H351Rfs*22 in total). (D) IFN-γ secretion by CD4^+^ T-blasts from controls, P2 and P4, and by CD4^+^ T-blasts from P2, P3, and P4 transduced with EV or WT LY9. Results from three experiments were compiled, with all technical replicates averaged. Non-transduced cells from P4 were tested twice. For P4’s cells, lentiviral transduction was conducted nine times as technical replicates. (E) Cytokine secretion by P4’s CD4^+^ T-blasts lentivirally transduced with EV, WT LY9, or biochemical mutants (*N*=10 for EV and WT; *N*=4 for YY; and *N*=3 for Y651F). Results from 10 experiments were compiled, with all technical replicates averaged. (F) *LY9* or *SH2D1A* knockout (KO) in CD4^+^ T-blasts from two healthy donors. Cells were nucleofected with Cas9 and either scrambled sgRNA or sgRNA pools for *LY9* and *SH2D1A*, expanded for 14 days, and restimulated for 2 hours in the presence of secretion inhibitors. The production of IFN-γ and TNF was assessed by flow cytometry. Technical duplicates were prepared for each sgRNA nucleofection. (G) Arrayed knockdown (KD) analysis. CD4^+^ T-blasts from two healthy donors with and without *LY9* KO were transduced with a lentivirus carrying shRNA and selected with puromycin. For *LY9* KO cells, LY9-negative cells was enriched by FACS. Two different scramble negative control (NC) shRNAs were tested and combined as “NC.”. Six technical replicates were prepared for each KD/KO. Representative results from two experiments. (H) Cytokine production by CD4^+^ T-blasts from controls, P2 (tested twice), P3, P4 (four times), one SAP-deficient patient (twice), one NFAT1-deficient patient (twice), and two RORγT-deficient patients, as measured by intracellular flow cytometry. Results from six experiments were compiled, with all technical replicates averaged. (I-K) RNASeq on CD4^+^ T-blasts from P2, P3, and P4 transduced with EV, WT LY9, or YY LY9. Geneset enrichment analysis (GSEA) was conducted on (I) transcription factor (TF) motifs, (J) Hallmark genesets, or (K) the T-helper signature genesets (68) (Method). Gray indicates non-significant results. In A-F and H, bars represent the mean and SEM. In A-E, G, and H, n.s., not significant; *, *P* < 0.05; **, *P* < 0.01; ***, *P* < 0.001; by unpaired Wilcoxon’s rank-sum tests. P/I, PMA and ionomycin.

As LY9 operates in a CD4^+^ T-cell-intrinsic fashion to promote IFN-γ production, we investigated the functional consequences of LY9 costimulation in *cis* by RNASeq on *LY9* KO HuT78 T-lymphoma cells transduced with EV or WT LY9 and stimulated with microbeads conjugated with anti-LY9 mAb or its isotype control (mouse IgG1), together with an anti-CD3 mAb. We found that *IFNG* was among the top 1% of genes for which expression was most strongly enhanced by LY9 crosslinking (fig. S21A and B). Consistently, RT-qPCR and flow cytometry showed that *LY9* KO reduced the LY9 crosslinking-dependent enhancement of IFN-γ production and that this deficit was rescued by lentiviral transduction with WT LY9 in HuT78 cells (Fig. 6A, S21C, and S21D). In CD4^+^ T-blasts, LY9 crosslinking enhanced the anti-CD3 mAb-driven expression of *IFNG* mRNA in CD4^+^ T-blasts from healthy controls, whereas this phenotype was impaired in LY9-deficient CD4^+^ T-blasts from P2, P3, and P4 (Fig. 6B). Conversely, lentiviral transduction with WT LY9 rescued the phenotype in CD4^+^ T-blasts from P2, P3, and P4 (Fig. 6B). IFN-γ protein production by CD4^+^ T-blasts was impaired in P2, P3, and P4, and this phenotype could be rescued by lentiviral transduction with WT LY9, as shown by ELISA and flow cytometry (Fig. 6C–E and S21E). In five healthy donors, T_H_1* (CCR4^−^CXCR3^+^CCR6^+^ memory) cells were the subset among PBMCs displaying the highest level of IFN-γ production upon TCR/LY9 crosslinking, followed by T_H_17 cells (fig. S22A). The LY9-dependent enhancement observed in T_H_17 cells probably reflects the plasticity between T_H_1* and T_H_17 cells. By contrast, T_H_1 cells and CD8^+^ αβ naïve and memory T cells displayed no such LY9-dependent enhancement of IFN-γ production (fig. S22A). No LY9-dependent enhancement of TNF production was observed in any of the subsets tested (fig. S22A). Thus, LY9 costimulation in *cis* selectively enhances IFN-γ production by T_H_1* cells.

### Enhancement of IFN-γ production in an NFAT1/RORγT-dependent manner by LY9 costimulation

SAP-deficient or *SH2D1A* KO CD4^+^ T-blasts displayed a normal LY9-dependent enhancement of IFN-γ production, and lentiviral transduction with WT or Y603A/Y626A mutant LY9 similarly rescued IFN-γ production in LY9-deficient CD4^+^ T-blasts (Fig. 6A, E, F, and S22B), suggesting that LY9 costimulation enhances IFN-γ production independently of SAP. Similarly, Y651F LY9, a mutant known to abolish the interaction between LY9 and GRB2 (65), rescued IFN-γ production similarly to WT LY9 (Fig. 6E). NFAT activates the transcription of *RORC* upon TCR crosslinking in human CD4^+^ αβ T lymphocytes, and LY9 drives NFAT to recruit RORγT to the *IL17A* promoter in human T lymphocytes (66, 67). We found that LY9 crosslinking increased *RORC* mRNA levels in CD4^+^ T-blasts from P2, P3, and P4 transduced with LY9 (WT or Y603A/Y626A) (fig. S22C), consistent with the low levels of RORγT in LY9-deficient CD4^+^ αβ T lymphocytes (Fig. 5E–G). Moreover, the shRNA-mediated knockdown of *LY9*, *NFAT1*, and *RORC* impaired the enhancement, by LY9 crosslinking, of IFN-γ production in CD4^+^ T-blasts from healthy controls (Fig. 6G and S22D). Likewise, NFAT inhibition with a cell-permeable 11R-VIVIT peptide in CD4^+^ T-blasts from P4 transduced with WT LY9 abolished the LY9 crosslinking-mediated enhancement of IFN-γ production (fig. S22E). Consistently, LY9 crosslinking did not enhance IFN-γ production in CD4^+^ T-blasts from two RORγT-deficient patients and one NFAT1-deficient patient, as in the cells of P2, P3, and P4, whereas cells from one SAP-deficient patient behaved normally (Fig. 6H). Thus, LY9 costimulation drives NFAT1 and RORγT to enhance IFN-γ production in T_H_1* cells.

### Enhancement of TCR-primed T_H_1* cell activation by LY9 costimulation

We performed a systematic analysis of the consequences of LY9 costimulation by performing bulk RNASeq analysis on CD4^+^ T-blasts from P2, P3, and P4 transduced with EV or LY9 (WT or Y603A/Y626A) and stimulated with bead-conjugated anti-CD3 and anti-LY9 mAbs for 1 hour. To avoid paracrine effects on the transcriptome, we added monensin and brefeldin A to prevent the secretion of cytokines and other soluble mediators. Analysis of the additive effect of LY9 crosslinking and TCR crosslinking revealed that an NFAT-binding motif (TGGAAA) as the only transcription factor motif significantly (FDR-adjusted *P* < 0.05) enriched following transduction with both WT and Y603A/Y626A LY9 and LY9 crosslinking (Fig. 6I). GSEA identified only four of the 50 Hallmark genesets as significantly (FDR-adjusted *P* < 0.05) upregulated in both WT- and Y603A/Y626A-transduced cells when analyzing the impact of TCR/LY9 crosslinking related to the non-stimulated state: IL-2/STAT5 signaling, inflammatory response, IFN-γ response, and TNF/NF-κB signaling (Fig. 6J). Furthermore, GSEA against the T_H_ signature genesets, defined as genes more abundantly expressed in one of the T helper subsets (T_H_1, T_H_2, T_H_17, or T_H_1*) relative to the others, based on the public RNASeq dataset for sorted T_H_ cell subsets (68), revealed an upregulation of T_H_1* signature genes and a downregulation of T_H_1 signature genes in both WT- and Y603A/Y626A-transduced cells with TCR/LY9 crosslinking (Fig. 6K). Thus, the LY9-NFAT1-RORγT axis forms a positive feedback loop to enhance the activation of TCR-primed T_H_1* cells.

## Discussion

We describe three unrelated patients (P1, P3, P4) with inherited complete LY9 deficiency and TB. The substantial enrichment observed for LY9 deficiency in TB patients (adjusted odds ratio = 81.7, *P* = 0.00012) identifies this condition as a genetic etiology of TB. Importantly, however, a fourth LY9-deficient individual, P2, the uncle of P1 from the first Moroccan kindred, has reached the age of 29 years without any notable infections, including TB, in his medical history. Thus, LY9 deficiency is probably highly, but incompletely, penetrant for TB. Throughout our attempt to systematically characterize the immunological abnormalities in LY9-deficient individuals, IFN-γ production was the most strikingly impaired immunological phenotype. Furthermore, LY9 deficiency seems to selectively impair IFN-γ production only in CD4^+^ αβ T lymphocytes. This partial IFN-γ deficiency is milder than the profound deficits seen in genetic etiologies of MSMD, such as complete T-bet and RORγT deficiencies (43, 45), probably accounting for the absence of vulnerability to weakly virulent mycobacteria in LY9-deficient individuals. CD4^+^ αβ T lymphocytes play a crucial role in protective immunity to *M.tb*, as demonstrated by the substantial vulnerability to TB of patients with AIDS (relative risk = 18.0 [95% CI: 15–21]) (2). Within CD4^+^ αβ T lymphocytes, T_H_1* cells (also known as T_H_1/17 cells) are an IFN-γ-producing CD4^+^ T-helper cell subset expressing T-bet and RORγT and known to be enriched in T cells specific for mycobacterial antigens (44). We found that 1) LY9 was expressed more strongly in T_H_1* cells than in other CD4^+^ T-cell subsets, 2) LY9 crosslinking induced IFN-γ production more strongly in T_H_1* cells than in other T-cell subsets, 3) LY9 crosslinking induced T_H_1* signature genes, including *RORC* and, conversely, suppressed T_H_2 signature genes, and 4) LY9 promoted IFN-γ production in response to mycobacteria in T_H_1* cells, T-bet^+^RORγT^+^ CD4^+^ αβ T lymphocytes, memory CD4^+^ T lymphocytes, cultured CD4^+^ T-blasts, and isolated *M.tb*-specific CD4^+^ T cell clones.

We propose that the LY9-dependent enhancement of IFN-γ production by T_H_1* cells occurs via a two-step mechanism (fig. S23). During the priming phase, LY9 induces T-bet and represses GATA3 via SAP and induces RORγT in a SAP-independent manner in naïve CD4^+^ αβ T lymphocytes engaged with DCs, presumably in the draining lymph nodes. T-bet and RORγT cooperatively program the primed naive CD4^+^ αβ T lymphocytes epigenetically, directing them to differentiate into T_H_1* cells with optimal IFN-γ-producing capacity. During the effector phase, LY9 enhances IFN-γ production via NFAT1 and RORγT but independently of SAP in T_H_1* cells recruited to the sites of *M.tb* infection. Consistently, pharmacological inhibition of NFAT1 and RORγT also impairs the *M.tb*- and APC-dependent enhancement of IFN-γ production in CD4^+^ T cells from healthy donors. It may appear counterintuitive that LY9 seems to control RORγT-related phenotypes in a SAP-independent manner in both the priming and effector phases. Given that patients with SAP deficiency, which underlies EBV-driven hematological disorders known as X-linked lymphoproliferative disease type 1 (XLP1), are not known to be vulnerable to TB, LY9 and RORγT-dependent but SAP-independent T_H_1* IFN-γ immunity is probably indispensable, more so than LY9 and SAP-dependent T-bet expression, for the control of *M.tb* infection. However, there may be an ascertainment bias, as only a few SAP-deficient patients living in areas of endemic TB have been diagnosed, with diagnosis typically occurring at a young age due to EBV-driven XLP1 (69). The limitation of the study includes a small number of LY9-deficient individuals identified. Studies of additional LY9-deficient individuals are crucial to obtaining an accurate estimate of the clinical penetrance of LY9 deficiency for TB, other intramacrophagic infections, and possibly other infections. Nevertheless, this study provides compelling evidence that IFN-γ production by T_H_1* cells is a non-redundant mechanism of human CD4^+^ T cell-dependent immunity to *M.tb*, even with quantitatively and functionally intact T_H_1 cells, consistent with several lines of clinical studies suggesting the association between T_H_1* cells and the disease status and severity of TB (70–72). Further investigations of the diagnostic and prognostic value of IFN-γ production by T_H_1* cells are necessary in patients with active TB and in immunocompromised individuals at high risk of TB.

## Materials and Methods

### Study design

We searched our in-house whole-exome sequencing (WES) database of TB patients (*N*=1,733) to identify unrelated TB patients homozygous for rare (minor allele frequency (MAF)<0.01) predicted loss-of-function (pLOF) variants in the same gene (Fig. 1A). We identified 1,631 biallelic pLOF variants of 1,199 unique genes. We excluded genes for which pLOF variants were also present in the homozygous state in an in-house control cohort (healthy individuals and patients with infectious diseases other than intramacrophagic diseases (i.e., those without MSMD, leprosy, Buruli ulcer, Whipple’s disease, salmonellosis, or related infections)) (*N*=21,274). We retained 381 variants of 361 genes, 15 of which had at least three variants. *LY9* was the only one of these genes reported to be expressed exclusively in hematopoietic cells in the Human Protein Atlas database (73). The human *LY9* gene is not under strong negative selection based on the CoNes scoring system (74), suggesting an autosomal recessive (AR) mode of inheritance. We found three unrelated patients with TB who were homozygous for pLOF variants of *LY9*. By contrast, we did not find any individuals homozygous for pLOF *LY9* variants in either the in-house control cohort (*N*=21,274) or our in-house cohorts of patients with other mycobacterial or intramacrophagic infectious diseases [MSMD, *N*=959; leprosy, *N*=3; Buruli ulcer, *N*=11; Whipple’s disease, *N*=62; and salmonellosis, *N*=60]. Individuals homozygous for pLOF *LY9* variants were, therefore, highly enriched in the cohort of TB patients, with a crude odds ratio (OR) tending to infinity. With adjustment for sex and ethnicity (principal components 1–5) and via Firth’s penalized logistic regression (75), we found a significant enrichment in pLOF *LY9* variants among TB patients (*N*=1,733) relative to controls without mycobacterial infection (*N*=21,274), assuming a recessive mode of inheritance (adjusted OR [95% CI] = 81.7 [7.9 – 10986.5], *P* = 0.00012). Assuming that 10% of individuals not homozygous for *LY9* pLOF variants develop TB following *M.tb* infection (76), this OR corresponds to a penetrance for TB of 90.1% in individuals homozygous for *LY9* pLOF variants (29). As a control, we did not observe any enrichment in pLOF variants of *LY9* in the heterozygous state or rare or common synonymous or in-frame non-synonymous variants of *LY9* in either the homozygous or heterozygous state (Table S1).

Based on the genetic finding, we set out to characterize the biochemical and immunological consequences of these variants both in isogenic overexpression experiments and in the patients’ cells. We found that the patients’ *LY9* variants abolish the expression and function of LY9 protein. We then performed in-depth *ex vivo* and *in vitro* experiments using PBMCs from the LY9-deficient patients and healthy controls to investigate the impact of LY9 deficiency on IFN-γ–dependent leukocytic immunity to *Mycobacterium tubreculosis*.

### Human subjects

The individuals with *LY9* mutations and their relatives, together with patients with biallelic loss-of-function (LOF) mutations of *IL12RB1*, *SH2D1A*, *TBX21*, and *RORC*, were recruited at Necker Hospital for Sick Children or The Garvan Institute of Medical Research. The patient with a biallelic LOF mutation of *NFATC2* was recruited at the University of British Columbia. Healthy volunteers were recruited at Rockefeller University. Written informed consent was obtained from all patients, family members, and healthy volunteers enrolled in this study. The study was approved by the institutional ethics committees of The Rockefeller University, Necker Hospital for Sick Children, ETH Zürich, The Garvan Institute of Medical Research, University of Barcelona, and McGill University and was performed in accordance with the requirements of these bodies. Experiments on samples from human subjects were conducted in the United States, France, Switzerland, and Australia, in accordance with local regulations and with the approval of the institutional review board of the corresponding institution.

### Whole-exome sequencing (WES) and variant filtering

WES was performed with genomic DNA (gDNA) extracted from whole-blood samples from the patients and their relatives, as previously described (77). Homozygosity rates were estimated as previously described (77). Variant blacklisting was performed as previously described (78). Minor allele frequencies (MAFs) in the general population, as reported in gnomAD database v2.1.1, and precomputed combined annotation–dependent depletion (CADD) scores (79) were used for variant filtering. The mutation significance cutoff (MSC) was calculated as previously described (80).

Aside from the *LY9* variants, we also identified rare biallelic pLOF variants of other genes (*SNX18*, *IDO2*, and *PLEC* in P1; none in P2 and P3; *LCE4A* and *INSRR* in P4) (fig. S1A). Among these genes, *SNX18* and *PLEC* were the only two known to be expressed in hematopoietic cells; we did not find any other TB or MSMD patients homozygous for rare pLOF variants in either *SNX18* or *PLEC* in our in-house database. Hence, none of these other genes were prioritized for further work-up.

### Sanger sequencing

PCR amplicons from gDNA or plasmids were sequenced with the BigDye Terminator Cycle Sequencing Kit (Applied Biosystems). Sequencing products were purified with Sephadex G-50 Superfine Resin (GE Healthcare). Sequences were determined with an ABI 3730 DNA Analyzer (Applied Biosystems). Sequencing spectrum data were analyzed with Geneious software (https://www.geneious.com).

### Estimation of the frequency of biallelic pLOF LY9 genotypes in the general population

The gnomAD database (https://gnomad.broadinstitute.org) contains 60 pLOF variants from 141,456 individuals, with a cumulative MAF of 1.9 × 10^−3^ (Fig. 1G and fig. S1D). We, therefore, estimated the frequency of biallelic pLOF genotypes at 3.6 × 10^−6^. Consistent with this estimate, only one individual in gnomAD was found to be homozygous for a pLOF variant (c.578del, P193Qfs*66). The c.182del and c.1052_1053del variants were not found in the homozygous state in gnomAD and had global MAFs of 9.9 × 10^−5^ and 2.6 × 10^−4^, respectively. The c.182del and c.1052_1053del variants were also rare or absent (MAF = 0.002 and 0, respectively; no homozygotes) from individuals of Greater Middle Eastern ancestry, which encompasses individuals of Moroccan and Turkish origin (81).

Despite its rarity in the general population, both the Moroccan patients (P1 and P3) were homozygous for the c.182del variant. These two patients were unrelated, with a WES-based kinship coefficient of zero (82). The frequency of the c.182del allele, as estimated by WES, for our Moroccan cohort was 0.020 [21/(531×2)]. The frequency of homozygotes in Morocco was therefore estimated at 4.0 × 10^−4^. Similarly, the estimated frequency of the c.1052_1053del allele in our Turkish cohort was 3.8 × 10^−3^ [7/(929×2)]. The frequency of homozygotes in Turkey was therefore estimated at 1.4 × 10^−5^. The rarity of biallelic pLOF *LY9* genotypes in multiple populations of different ancestries strongly suggests that *LY9* is non-redundant in humans.

### Plasmids

A full-length wild-type (WT) human LY9 coding sequence (CDS) from a pEZ-M68 vector (GeneCopoeia, Cat: EX-Z2640-M68) was inserted into a pcDNA3.1 backbone for transient transfection. A WT LY9 CDS with a DYKDDDDK tag was generated by PCR, with the tag sequence added either directly upstream from the original stop codon or directly upstream from the newly formed stop codon resulting from a frameshift mutation. The patients’ frameshift variants and all homozygous variants found in either our in-house database [V78I (in one patient with non-mycobacterial infectious diseases), S171del (in two MSMD/TB patients and four patients with non-mycobacterial infectious diseases), P196T (in one patient with MSMD/TB and three patients with non-mycobacterial infectious diseases), and N413S (in three patients with non-mycobacterial infectious diseases)] or the gnomAD database (I69N, R91H, R106Q, E152G, S171del, P193Qfs*66, P196T, V346I, N413S, V468I, and M602V) were constructed through site-directed mutagenesis. Only two (V468I and M602V) of these 11 variants have a MAF > 0.01. The entire CDS was validated by Sanger sequencing. The WT or mutant LY9 CDS without a DYKDDDDK tag was then inserted into a pTRIP-CMV-Puro-2A backbone (Addgene plasmid #102611, a gift from Nicolas Manel (83)) for lentiviral transduction.

### Cells

The HEK293T cell line was purchased from the ATCC and cultured in Dulbecco’s modified Eagle medium (DMEM; Gibco) supplemented with 10% fetal bovine serum (FBS; Gibco). The HuT78 T-lymphoma, Raji B-lymphoma, and THP-1 monocytic leukemia cell lines were purchased from the ATCC and cultured in Roswell Park Memorial Institute (RPMI)-1640 medium with GlutaMAX (Gibco), supplemented with 10% FBS (referred to hereafter as lymphocyte medium). Peripheral blood mononuclear cells (PBMCs) were isolated by the Ficoll-Hypaque density gradient centrifugation (GE Healthcare) of venous blood samples and cryopreserved at −150°C until use. PBMCs were thawed and left to rest temporarily in lymphocyte medium during experiments. Epstein-Barr virus-transformed B (EBV-B) cell lines were generated in-house by infecting PBMCs with EBV and cultured in lymphocyte medium. T-cell blasts (T-blasts) were generated by culturing PBMCs in ImmunoCult XF T-Cell Expansion Medium (STEMCELL Technologies, Cat: 10981) containing recombinant human interleukin 2 (rhIL-2; Roche, Cat: 11147528001) at a final concentration of 10 ng/mL and ImmunoCult Human CD3/CD28/CD2 T-Cell Activator (STEMCELL Technologies, Cat: 10970; 1:100). T-blasts were propagated by adding fresh medium containing rhIL-2 every 48–72 hours and were restimulated with ImmunoCult Human CD3/CD28/CD2 T-Cell Activator after 10 to 14 days of expansion. T-blasts cultured for at least seven days after induction or restimulation were used for experiments. CD4^+^ T-blasts were isolated either by fluorescence-activated cell sorting (FACS) with a FACS Aria cell sorter (BD Biosciences) or by magnetic-activated cell sorting (MACS) with CD4 MicroBeads (Miltenyi Biotec, Cat: 130–045-101).

### Lentiviral transduction

Lentiviruses were prepared by transfecting HEK293T cells with the pTRIP-CMV-Puro-2A plasmid (EV) or the vector encoding WT or mutant LY9 CDS, as previously described (32). For gene knockdown experiments, we used small hairpin RNA (shRNA) lentiviral plasmids purchased from Santa Cruz Biotechnology. T-blasts were reactivated 2 to 3 days, followed by spinoculation as previously described (32). HuT78 cells were transduced in lymphocyte medium.

### Gene knockout (KO)

Pools of three single guide RNAs (sgRNAs) for *LY9* (CAAAGAGUCACACAGAUGAC, AUUUGCAGCUGACUCCUCUG, AAGUGGACUUACCCAUGAGC), *SH2D1A* (GACGCAGUGGCUGUGUAUCA, GGAUGGCAGCUAUUUGCUGA, AUCAUACUCACAGCACACAU), and a scrambled negative control RNA (GCACUACCAGAGCUAACUCA) were purchased from Synthego. TrueCut Cas9 v2 (Invitrogen, Cat: A36499) and sgRNA were mixed at an equal molar ratio (150 to 300 pmol) and incubated at room temperature for 30 minutes. FACS-sorted CD4^+^ T-blasts from two healthy donors or Raji cells (2 × 10^6^ cells) were nucleofected with the ribonucleoprotein complex with the Amaxa Human T-Cell Nucleofector Kit (Lonza, Cat: VPA-1002) or Amaxa Cell Line Nucleofector Kit V (Lonza, Cat: VCA-1003). For *LY9* KO, cells were stained with anti-LY9 mAb, and LY9-negative populations were sorted by FACS and further expanded *in vitro*. Simultaneously prepared negative control-nucleofected cells were also sorted to obtain LY9-positive populations as a control.

### Analysis of LY9 expression by immunoblotting

HEK293T cells were transiently transfected with LY9-encoding plasmids, and analyzed by sodium dodecyl sulfate-polyacrylamide gel electrophoresis (SDS-PAGE) and immunoblotting with anti-DYKDDDDK and anti-GAPDH antibodies, as previously described (31).

### Analysis of LY9 expression by reverse transcription-quantitative PCR (RT-qPCR)

Total RNA was extracted from EBV-B cells with the Quick-RNA Miniprep kit (Zymo Research, Cat: R1054) and reverse-transcribed with SuperScript IV Reverse Transcriptase (Invitrogen, Cat: 18090050). The cDNA was analyzed with the FAM-MGB TaqMan probes for *LY9* (Hs03004330_m1 and Hs01015562_m1), as described previously (31).

### Analysis of LY9 expression by flow cytometry

Transiently transfected HEK293T cells were detached from the wells by incubation with TryPLE Express (Gibco, Cat: 12604013) for 5 minutes at 37°C. HEK293T cells and EBV-B cells were stained by incubation with anti-LY9-PE antibody (BioLegend, Clone: HLy-9.1.25, Cat: 326108, 1:100) or mouse IgG1 isotype control-PE (eBioscience, Clone: P3.6.2.8.1, Cat: 12–4714-41) in FACS buffer (2% FBS and 2 mM EDTA in PBS, filter-sterilized) supplemented with FcR blocking reagent (Miltenyi Biotec, Cat: 130–059-901, 1:50) and 0.1% sodium azide for one hour at 4°C in the dark. We then added 7-aminoactinomycin D (7-AAD; Tonbo Biosciences, Cat: 13–6993; 1:200) and incubated the mixture for a further 10 minutes at 4°C in the dark. T-blasts were analyzed in a similar manner, but with anti-CD4 and anti-CD8 antibodies. The cells were acquired with a BD LSR II Flow Cytometer (BD Biosciences). Data were analyzed with FlowJo software v10 (FlowJo, LLC) and R.

PBMCs were stained with LIVE/DEAD Fixable Aqua (Invitrogen, Cat: L34957, 1:1000 in PBS) for 15 minutes 4°C in the dark, and then with the staining reagents (Table S4, Panel 1) in FACS buffer containing 0.1% sodium azide for 30 minutes at 4°C in the dark. The cells were acquired with a BD LSR II flow cytometer (BD Biosciences). Data were analyzed with FlowJo and R.

We then assessed LY9 expression comprehensively on all blood leukocyte subsets by flow cytometry. PBMCs (1.0 to 1.5 × 10^6^ cells) were stained as previously described (32), with the APC channel replaced to anti-LY9-APC (eBioscience, Cat: 17–2299-42, Clone: HLy9.25, 1:100) antibody.

We also characterized the surface expression of LY9 protein in leukocyte subsets upon activation. Briefly, PBMCs (5 × 10^6^ cells) were first surface-stained in PBS plus 2% FBS with the staining reagents (Table S4, Panel 2, Surface 1) for 30 minutes at 4°C in the dark. The cells were then directly mixed and incubated with the second surface-staining mix (Table S4, Panel 2, Surface 2) in PBS plus 2% FBS for an additional 30 minutes at 4°C in the dark. After surface staining, the cells were centrifuged, washed once with lymphocyte medium, and resuspended in lymphocyte medium with gentamicin (1:500). Cells were dispensed into a U-bottom 96-well plate at a density of ~0.5 × 10^6^ cells/100 μL/well and were either left non-stimulated or were stimulated with Cell Stimulation Cocktail (eBioscience, Cat: 00–4970-93; 1:1000) for 1 hour. Cells were transferred to a V-bottom 96-well plate and centrifuged to obtain a cell pellet, which was washed once with PBS, and stained with Ghost Dye UV450 (Cytek, Cat: SKU 13–0868-T100, 1:1000 in PBS) for 15 minutes at 4°C in the dark. FcR blocking reagent and anti-LY9-PE mAb (BioLegend, Clone: HLy-9.1.25, 1:100) or isotype control mAb (BioLegend, Clone: MOPC-21) in FACS buffer were added directly to the cells, which were incubated for an additional 30 minutes at 4°C in the dark. The cells were then washed, fixed by incubation in 1% PFA/PBS for 15 minutes at 4°C in the dark, washed again, and acquired with an Aurora Cytometer (Cytek). Subsets were manually gated with FlowJo, and LY9 expression levels were analyzed with R.

We assessed the surface expression of LY9 protein on macrophages by isolating CD14^+^ monocytes from cryopreserved PBMCs from healthy donors with anti-CD14 microbeads (#130–050-201, Miltenyi Biotec). M1- and M2-polarized macrophages were obtained by differentiating monocytes in M1-Macrophage Generation Medium XF (#C-28055, PromoCell) supplemented with GM-CSF, or Macrophage Base Medium XF (#C-28057, PromoCell) supplemented with M-CSF, respectively, for 10 days, in accordance with the manufacturer’s instructions. M2a-polarized macrophages were induced by incubation in RPMI supplemented with 10% FCS and M-CSF (50 ng/mL; #216-MC, R&D Systems) for seven days, and then with M-CSF (50 ng/mL) and IL-4 (50 ng/mL; #204-IL, R&D Systems) for an additional seven days. LY9 expression was evaluated by flow cytometry after surface staining for 30 minutes on ice with an anti-LY9-PE (#326107, BioLegend, 1:25) antibody or the corresponding isotype (mIgG1k-PE; #400112, BioLegend, 1:100). The cells were acquired with a Gallios flow cytometer and analyzed with FlowJo.

### Preparation of antibody-conjugated beads

Antibody-conjugated beads were prepared as previously described (31, 84) with the following monoclonal antibodies: Ultra-LEAF purified anti-CD3 antibody (BioLegend, Cat: 317236, Clone: OKT3, 8 μg), Ultra-LEAF purified mouse IgG1 isotype control (BioLegend, Cat: 400166, Clone: MOPC-21, 90 μg), and Ultra-LEAF purified anti-human LY9 antibody (BioLegend, Cat: 326109, Clone: HLy-9.1.25, 90 μg).

### ERK phosphorylation assays in T-blasts

T-blasts (2 × 10^5^ cells) were serum-starved and surface-stained in RPMI-1640 medium plus 1% FBS without IL-2 supplemented with anti-CD4-BUV395 (BD Biosciences, Cat: 563552, Clone: SK3, 1:100) and anti-CD8-BUV737 (BD Biosciences, Cat: 612755, Clone: SK1, 1:100) antibodies in an incubator at 37°C for 2 hours. They were then washed with PBS and stained by incubation with LIVE/DEAD Fixable Aqua (Invitrogen, 1:1000 in PBS) in 50 μL PBS for 15 minutes 4°C in the dark. Stimuli in 50 μL RPMI-1640 medium plus 1% FBS were added directly to the cells on ice. The cells were then incubated for 15 minutes at 37°C, fixed by the direct addition of 100 μL BD Cytofix Fixation Buffer (BD Biosciences, Cat: 554655) and incubated at room temperature for 15 minutes in the dark. The cells were then permeabilized by incubation with 100% methanol at −20°C overnight and stained by incubation with anti-phospho-ERK1/2-Alexa Fluor 647 antibody (BioLegend, Cat: 369504, Clone: 6B8B69, 1:20) at room temperature for 3 hours in the dark. They were then washed three times in FACS buffer and acquired with a BD LSR II Flow Cytometer (BD Biosciences).

### Immunophenotyping of primary leukocytes by flow cytometry

PBMCs (1.0 to 1.5 × 10^6^ cells) were immunophenotyped as previously described (32). CD4^+^ αβ T helper cell subsets were identified based on the expression of CXCR3, CCR4, and CCR6 (Fig. 5A and fig. S15A). B-cell-specific phenotypes were analyzed as previously described (85).

### T-cell receptor (TCR) repertoire analysis

The TCR α-chain (TRA) repertoire was captured by sequencing whole-blood genomic DNA samples from P2, P4, and three healthy donors (Adaptive Biotechnologies), as previously described (31). Clonotype analysis was performed as described previously (86).

### VirScan analysis

Serum IgG was isolated from P4, P4’s relatives, three healthy controls, and three TB patients. The reactivity of isolated IgG was profiled against a library of phages displaying linear 45-amino acid epitopes derived from diverse microbes, as previously described (31). For *Mycobacterium*-derived peptides, the upper 95^th^ percentile of peptide reactivity scores for the negative controls (i.e., IgG-depleted serum samples and mock IP controls), which was equal to 0.55, was used as a cutoff. For pan-microbe analysis, the upper 99.9^th^ percentile was used as a cutoff. Peptides with scores above the cutoff were considered to be positive hits. The aggregate score was defined as the sum of reactivity scores for all positive peptides per sample.

### Serum autoreactivity profiling

Serum autoantibody reactivity was studied with human full-length protein arrays (ProtoArray v5.1, PAH05251020, Thermo Fisher Scientific). Serum samples from P4, P4’s relatives, a patient with APS1, and three healthy donors were investigated. Protein arrays were probed with serum at a dilution of 1:2000, and the manufacturer’s protocol (*Immune response biomarker profiling*) was otherwise followed. Protein arrays were first incubated with blocking buffer (PA055, Life Technologies) for 1 hour, blocked by incubation with serum at a dilution of 1:2000 for 90 minutes, and finally incubated with detection antibodies for 90 minutes: Alexa Fluor 647 goat anti-human IgG antibody (A21445, Thermo Fisher) Scientific at a 1:2000 dilution and Dylight 550 goat anti-GST (#DY550011–13-001, Cayman Chemicals) at a 1:10000 dilution. The Innopsys InnoScan 1100 AL 3-channel ultrahigh-resolution microarray scanner was used. Data were analyzed with R. Differential reactivity analysis was conducted with limma (87).

### Fluorescence-activated cell sorting (FACS)

PBMCs were stained with an anti-CCR7-BV750 mAb (BioLegend, Clone: G043H7; 1:20) in FACS buffer for 30 minutes at 4 °C in the dark. The cells were then directly mixed with the staining reagents (Table S4, Panel 3) in FACS buffer and incubated for an additional 30 minutes at 4 °C in the dark. Cells were then mixed with 7-amino-actinomycin D (7-AAD; Tonbo Biosciences, 13–6993; 1:200 dilution) in FACS buffer to make the final volume up to ~500 μL. The cells were then filtered with a Falcon cell strainer tube (Corning, 352235) and sorted with a FACS Aria instrument (BD Biosciences). The following cell subsets were sorted into 5 mL polypropylene tubes containing 1 mL lymphocyte medium supplemented with gentamicin (1:500): naïve B cells (CD3^−^CD20^+^CD38^−^CD10^−^CD27^−^), memory B cells (CD3^−^CD20^+^CD38^−^CD27^+^), CD4^+^ naïve T cells (CD20^−^CD3^+^CD4^+^CD45RA^+^CCR7^+^), and CD4^+^ memory T cells (CD20^−^CD3^+^CD4^+^CD45RA^−^CCR7^+/−^ and CD45RA^+^CCR7^−^) (Fig. 5C and fig. S6B). Cells were kept on ice during sorting.

### Naïve and memory B-cell stimulation assay

Sorted naïve and memory B cells were stimulated *in vitro* for analyzing the secretion of IgM, IgA, and IgG, as described previously (85). A subset of data for healthy controls were published previously (85).

### Single-cell RNA sequencing (scRNASeq) analysis of primary leukocytes at rest

Cryopreserved PBMCs from nine healthy adult controls, of whom one was tested twice, P2, P3, P4, and three IL-12Rβ1-deficient patients were analyzed by single-cell RNA sequencing (scRNASeq), as previously described (27). Approximately 10,000 cells were sequenced per sample. Quality-control filtering, unsupervised clustering, and manual cell-type annotation were performed as previously described (27). Expression levels for representative marker genes in each cluster were quantified with Seurat (88). Pseudobulk differential expression analysis (89) was performed with DESeq2 (90). Geneset enrichment analysis (GSEA) was conducted with fgsea by projecting genes ranked by their fold-changes in expression with effect-size shrinkage (91) onto the genesets retrieved from the MSigDB database (https://www.gsea-msigdb.org/gsea/msigdb/).

### *Analysis of leukocyte responses to* M.tb *by scRNASeq*

Cryopreserved PBMCs from two healthy adult controls, P2, and P4 were either left non-stimulated or were stimulated with heat-killed *M.tb* (HKMTb; InVivoGen, Cat: tlrl-hkmt-5, 400 μg/mL) for 6 hours. HKMTb was resuspended in endotoxin-free water and filtered through a 40 μm-mesh Falcon Cell Strainer (Corning, Cat: 352340) before use to prevent the clogging of the 10X microfluidics with large items of debris. After stimulation, cells were washed three times with 0.5% FBS in PBS, filtered through a 40 μm-mesh Falcon Cell Strainer (Corning, Cat: 352340), and subjected to single-cell sequencing as described above. Approximately 10,000 cells were sequenced per sample. Cell types were identified by unsupervised clustering and manual inspection. Pseudobulk DE for the HKMTb vs. NS log_2_FC in LY9-deficient cells relative to control cells was determined with DESeq2. GSEA was performed with fgsea to determine whether each cytokine-dependent DEG set was significantly enriched or depleted in terms of its fold-change in expression ranking in LY9-deficient cells. For immune response enrichment analysis (IREA) (48), precompiled Seurat datasets were downloaded from the Immune Dictionary web portal (https://www.immune-dictionary.org/app/home) for the following leukocyte subsets: CD4 T cells, CD8 T cells, γδ T cells, Tregs, B cells, NK cells, monocytes and macrophages (combined), cDC1 and cDC2 (combined), and pDCs. Mouse-to-human gene symbol conversion was performed according to HomoloGene build 68. Our dataset for a given leukocyte was normalized via the *SCTransform* framework, with regression against the percentage of mitochondrial genes and then projection onto the reference dataset for a given cytokine or PBS (preprocessed in the same manner) via the *FindTransferAnchors* and *TransferData* framework implemented in Seurat v5 (92, 93). The probability of a given cell being assigned to the cytokine concerned (and not to PBS) was used as a metric for assessing cytokine activity. The area under the ROC curve (AUC) was then computed via the fast implementation in Presto to obtain a quantitative assessment of the induction or repression of the activity of a given cytokine by HKMTb relative to the non-stimulated state. High AUC values indicate an HKMTb-induced upregulation of cytokine activity. The difference between the AUC values for control and LY9-deficient cells (ΔAUC) was used to assess the impairment attributable to LY9 deficiency. Only cytokine-cell type pairs with an AUC ≥ 0.8 in control cells were selected for visualization in the heatmap. Principal component analysis of the activity of 86 cytokines was performed in FactoMineR. For the representative gene analysis contributing to the IFN-γ activity, the genes in the reference dataset displaying differential expression were obtained from the *FindMarkers* framework in Seurat.

### Analysis of cytokine secretion by stimulated leukocytes

PBMCs were dispensed into a U-bottom 96-well plate (1 × 10^5^ cells in 100 μL lymphocyte medium per well) and were either left non-stimulated or were stimulated with lipopolysaccharides from *Salmonella enterica* serotype Minnesota (LPS; Sigma-Aldrich, Cat: L4641, 10 ng/mL), HKMTb (InVivoGen, 100 μg/mL), phytohemagglutinin-M (PHA; Gibco, Cat: 10576015, 1:100), Dynabeads^™^ Human T-Activator CD3/CD28 (Thermo Fisher Scientific, Cat: 11161D, Bead:Cell=1:1), ImmunoCult^™^ Human CD3/CD28/CD2 T Cell Activator (STEMCELL, Cat: 109700, 1:100), or Cell Stimulation Cocktail (eBioscience, Cat: 00–4970-93, 1:1000) for 24 hours at 37°C. For autologous T- and B-lymphocyte engagement assay, PBMCs, were cultured in lymphocyte medium supplemented with IL-2 (Roche, 10 ng/mL) and stimulated with anti-CD19-anti-CD3 bispecific antibody (BPS Bioscience, Cat: 100441–1, 10 ng/mL) at 37°C for 5 days. Supernatants were harvested and stored at −20°C until use. The levels of secreted cytokines were measured in a LEGENDplex assay (Human Th Cytokine Panel V02 or Human Macrophage/Microglia Panel, BioLegend, Cat: 741027 or 740503) and with the Circulex Human ISG15 ELISA kit (MBL International, Cat: CY8085).

### Analysis of cellular responses to live BCG by flow cytometry

PBMCs were stimulated with IL-12 or IL-23, with or without live BCG mycobacteria, and analyzed via flow cytometry, as previously described (32). Data were analyzed in R.

### *Analysis of cellular responses to* M.tb *by flow cytometry*

PBMCs (3 × 10^6^ cells) were first surface-stained in PBS plus 2% FBS with the staining reagents (Table S4, Panel 4, Surface 1) for 30 minutes at 4°C in the dark. The cells were then directly mixed with the second surface-staining mix (Table S4, Panel 4, Surface 2) in PBS plus 2% FBS and incubated for an additional 30 minutes at 4°C in the dark. The cells were then centrifuged, washed once with lymphocyte medium, and resuspended in lymphocyte medium supplemented with gentamicin (1:500), brefeldin A (Cytek, Cat: SKU TNB-4506-L001, 1:1000), and monensin (Cytek, Cat: SKU TNB-4505-L001, 1:1000). Cells were dispensed into a U-bottom 96-well plate at a density of ~0.5 × 10^6^ cells/100 μL/well and were either left non-stimulated or were stimulated with filtered HKMTb (InVivoGen, 400 μg/mL), CytoStim (Miltenyi Biotec, Cat: 130–092-172, 1:100), HKMTb + CytoStim, ImmunoCult^™^ Human CD3/CD28/CD2 T Cell Activator (STEMCELL, 1:100), or Cell Stimulation Cocktail (eBioscience, 1:1000) for 6 hours at 37°C. The cells were transferred to a V-bottom 96-well plate, centrifuged to obtain a cell pellet, washed once with PBS, and stained with Ghost Dye Violet 510 (Cytek, Cat: SKU 13–0870-T100, 1:1000 in PBS) for 15 minutes at 4°C in the dark. The cells were then washed once with FACS buffer and fixed with the FOXP3/Transcription Factor Staining Buffer Kit (Cytek) at room temperature for 45 minutes in the dark. The cells were then washed twice in permeabilization buffer and stained overnight in the intracellular staining reagents (Table S4, Panel 4, ICS) in permeabilization buffer. The cells were washed three times with FACS buffer and acquired with an Aurora Cytometer (Cytek). The data were analyzed with FlowJo and R (fig. S9A). Primary readouts (% IFN-γ^+^ or TNF^+^ cells) were normalized against those in total CD4^+^ αβ T lymphocytes to show the relative contribution of each T_H_ compartment within a given donor.

### THP-1:CD4^+^ T-cell coculture assay

THP-1-derived macrophages were prepared by incubating THP-1 cells with PMA (Sigma-Aldrich, Cat: P8139–1MG, 50 ng/mL (1:246,000 of a stock solution at 12.3 mg/mL in DMSO)) in lymphocyte medium at a density of 1 × 10^6^ cells per 1 mL of lymphocyte medium in a U-bottom 96-well plate (5 × 10^4^ cells per 50 μL per well) for 48 hours. The cells became adherent after differentiation. The cells were washed once with PBS and then cocultured with MACS-enriched CD4^+^ T-blasts (2 × 10^4^ cells per well) and stimuli [filtered HKMTb (InVivoGen, 400 μg/mL), CytoStim (Miltenyi Biotec, 1:1000), HKMTb + CytoStim, or Cell Stimulation Cocktail (eBioscience, 1:1000)] in 100 μL lymphocyte medium for 18 hours. For comparison, CD4^+^ T-blasts were cultured in isolation or with non-differentiated THP-1 cells (5 × 10^4^ cells per well) with or without stimuli. The amounts of cytokines secreted were determined with a LEGENDplex assay (Human Th Cytokine Panel V02, BioLegend).

### Raji:CD4^+^ T-cell coculture assay

Raji B-lymphoma cells underwent *LY9* knockout (R-KO) or were treated with a scramble negative control sgRNA (R-NC) and expanded for at least seven days. LY9 expression was undetectable on the surface of R-KO cells. MACS-enriched CD4^+^ T-blasts (2 × 10^4^ cells per well) and Raji cells (2 × 10^5^ cells per well) were cocultured in lymphocyte medium supplemented with IL-2 (Roche, 10 ng/mL) with or without anti-CD19-anti-CD3 bispecific antibody (BPS Bioscience, 10 ng/mL) in 100 μL lymphocyte medium for three days. The amounts of cytokines secreted were determined with a LEGENDplex assay (Human Th Cytokine Panel V02, BioLegend).

### Listeria *growth suppression assay*

*Listeria monocytogenes* (1/2a) stably expressing GFP was purchased from Microbiologics (Cat: 01249UV-V, Lot: 1249–13-1). One lyophilized pellet was resuspended in 1 mL of lymphocyte medium and split into aliquots, which were stored at −80°C. THP-1 cells were first stained with CellTrace Far Red (Thermo Fisher Scientific, C34572, 1:1000 in PBS) for 10 minutes at 37°C, and then infected with GFP-*L. monocytogenes* (used at 1:100, 1:1000, or 1:10000, roughly corresponding to an MOI=0.01, 0.001, or 0.0001, respectively, based on a preliminary analysis of the proportions of infected cells by flow cytometry) in lymphocyte medium (5 × 10^5^ cells per mL) in six-well plates for 90 minutes at 37°C. After infection, the cells were harvested by centrifugation and resuspended in fresh lymphocyte medium with or without gentamicin (1:500). THP-1 cells infected with GFP-*L. monocytogenes* (5 × 10^4^ cells per well) were then cocultured with or without MACS-enriched CD4^+^ T-blasts (2 × 10^5^ cells per well) and with or without filtered HKMTb (InVivoGen, 400 μg/mL) or Cell Stimulation Cocktail (eBioscience, 1:1000) for 20 hours at 37°C. Cells were transferred to a V-bottom 96-well plate, harvested by centrifugation, washed once with PBS, and stained with Ghost Dye Violet 510 (Cytek, 1:1000 in PBS) for 15 minutes at 4°C in the dark. They were then washed, fixed by incubation in 1% PFA/PBS for 10 minutes at 4°C in the dark, washed again, and acquired with an Attune NxT Flow Cytometer with the CytKick MAX Autosampler (Invitrogen). Based on our preliminary testing, these mild fixation conditions did not substantially decrease the GFP signal relative to non-fixed and no-GFP controls. The data were analyzed with FlowJo and R (fig. S12B).

### Analysis of antigen-specific CD4^+^ αβ T-cell clones

PBMCs from P4, P4’s ethnicity-matched travel control, and healthy controls were labeled with CFSE (Invitrogen, Cat: C1157, 5 μM) in PBS plus 5% human serum and stimulated with gamma-irradiated *M.tb* H37Rv whole-cell lysate (5 μg/mL, Cat: NR-14822, BEI Resources, NIAID, NIH), heat-inactivated *C. albicans* (10^5^ particles per well), or were incubated without antigen. IL-2 (50 IU/mL) was added on day 6. Using a FACSAria Fusion cell sorter (BD Biosciences), we identified proliferating T cells by CFSE dilution on day 22 after stimulation. These cells were sorted singly into 96-well plates containing complete RPMI medium, irradiated allogeneic PBMCs as feeder cells (10^5^ cells/well), PHA (1 μg/mL), and IL-2 (500 IU/mL). After 21 days, clones were stimulated under various conditions (5 × 10^4^ cells/well) for 6 hours: 1) plate-bound anti-CD3 mAb (clone TR66, 1 μM); 2) ImmunoCult^™^ Human CD3/CD28/CD2 T-Cell Activator (STEMCELL); 3) PMA (0.2 μM) and ionomycin (1 μg/mL); 4) PHA (1 μg/mL); 5) medium. Brefeldin A (10 μg/mL) was added for the last four hours of stimulation. Intracellular cytokines and transcription factors were stained with the eBioscience FOXP3/Transcription Factor Staining Buffer Set (Invitrogen, Cat: 00–5523-00) according to the manufacturer’s instructions and with the following antibody panel: anti-T-bet-APC (Miltenyi Biotec, Clone REA102, Cat: 130–119-821), anti-GATA3-PE/Vio 615 (Miltenyi Biotec, Clone: REA174, Cat: 130–109-161), anti-RORγT-PE (BD Biosciences, Clone: Q21–559, Cat: 563081), anti-IFN-γ-BUV395 (BD Biosciences, Clone: B27, Cat: 563563), anti-IL-17A-BV605 (BioLegend, Clone: BL168, Cat: 512325), anti-IL-4-BV711 (BD Biosciences, Clone: MP4–25D2, Cat: 564112), anti-TNF-BV785 (BioLegend, Clone: MAb11, Cat: 502947), anti-IL-22-BUV737 (eBioscience, Clone: 22URTI, Cat: 367–7229-42), and anti-Granzyme B-Pacific Blue (BioLegend, Clone: GB11, Cat: 515407). Dead cells were labeled with LIVE/DEAD Fixable Aqua (Invitrogen). The cells were analyzed with a BD LSRFortessa Cell Analyzer (BD Biosciences). The expression levels of IFN-γ, TNF, or IL-4, T-bet, and RORγT were quantified with FlowJo (fig. S11B, S11C, S19A, and S19B).

A mixed-effects linear regression analysis of the relationship between the percentage of cells producing IFN-γ, TNF, or IL-4 and the MFI of T-bet or RORγT was performed in R using an nlme package (fig. S20A–D). The model was estimated through a maximum likelihood method and an nlminb optimizer. The MFI of T-bet or RORγT and genotype (controls vs. LY9 deficiency) were included as fixed effects, while stimulation, donor IDs, and clone IDs were included as random effects. The significance of genotype as a predictor was determined by a likelihood ratio test between two mixed-effects models with or without each genotypes as a variable. Both T-bet and RORγT levels were significantly associated with higher levels of IFN-γ production (T-bet: *P* < 1 × 10^−4^, marginal R^2^ = 0.13, conditional R^2^ = 0.92; RORγT: *P* < 1 × 10^−4^, marginal R^2^ = 0.13, conditional R^2^ = 0.90) (fig. S20E and F). By contrast, T-bet and RORγT levels were not significantly correlated with the production of TNF (*P* = 0.65 and 0.36 for T-bet and RORγT, respectively) or IL-4 (*P* = 0.18 and 0.24 for T-bet and RORγT, respectively) based on similar mixed-effects modeling.

*M.tb*-specific CD4^+^ αβ T-cell clones from P4 (*N*=3 clones), P4’s ethnicity-matched travel control (*N*=1 clone), and two healthy controls (*N*=1 clone each) with high levels of RORγT expression were selected at random for further analysis. Cryopreserved clones were thawed and expanded further for another two weeks. The cells were dispensed into a U-bottom 96-well plate (5 × 10^4^ cells in 100 μL lymphocyte medium per well) and stimulated by incubation for two hours with in-house anti-CD3 mAb-conjugated beads (cell:bead ratio=1:4), Dynabeads Human T-Activator CD3/CD28 (Gibco, cell:bead ratio=1:4), PHA-M (Gibco, 1:100), or Cell Stimulation Cocktail (eBioscience, 1:1000) at 37°C. The supernatants were harvested and stored at −20°C until use. The levels of IFN-γ secreted into the supernatants were determined with the IFN gamma Human ProQuantum Immunoassay Kit (Invitrogen, Cat: A35576). RNA was extracted from the cell pellets for bulk RNASeq analysis, as previously described (85). Sequence alignment, quantification of gene-level features, and differential expression (DE) analysis were performed as previously described (85). Transcripts per million (TPM) were computed using edgeR (94) (fig. S11F and S17D).

### Analysis of cytokine production by sorted naïve and memory CD4^+^ T lymphocytes

Naïve and memory CD4^+^ T cells were sorted from the cryopreserved PBMCs of P4 and two healthy controls, as previously described (95) (fig. S15C), cultured with T-cell activation and expansion (TAE) beads (anti-CD2/CD3/CD28; Miltenyi Biotec) plus IL-2 for seven days, and then under T_H_0 conditions (TAE beads alone) for five days. Secreted cytokine levels were assessed with the BD cytometric bead array (BD Bioscience), whereas cytokine levels at the end of culture were assessed by intracellular flow cytometry. Naïve and memory CD4^+^ T cells were sorted from the cryopreserved PBMCs of P3 and two healthy controls and cocultured with THP-1 alone or THP-1 plus HKMTb, as described above. Secreted cytokine levels were assessed with LEGENDplex Human CD8/NK Panel V02 (BioLegend, Cat: 741187). The ratio of the amount of cytokines produced by memory CD4^+^ T cells to the amount of cytokines produced by their naïve counterparts was calculated to evaluate the enhancement of cytokine-producing capacity due to naïve-to-memory differentiation *in vivo*.

### Analysis of transcription factors in PBMCs and CD4^+^ T-blasts

PBMCs were dispensed into a U-bottom 96-well plate (1 × 10^5^ cells in 100 μL lymphocyte medium per well) and were either left non-stimulated or were stimulated with PHA-M (Gibco, 1:100), ImmunoCult^™^ Human CD3/CD28/CD2 T Cell Activator (STEMCELL, 1:100), or Cell Stimulation Cocktail (eBioscience, 1:1000) for 24 hours at 37°C. The cells were stained by incubation with Zombie NIR Fixable Viability dye (BioLegend, Cat: 423105, 1:1000 in PBS) for 15 minutes at 4°C in the dark, and then with the staining reagents (Table S4, Panel 5, Surface) in FACS buffer supplemented with 0.1% sodium azide for 1 hour at 4°C in the dark. The cells were then washed with FACS buffer and fixed and permeabilized with the FOXP3/Transcription Factor Staining Buffer Kit (Cytek, Cat: SKU TNB-0607-KIT). The cells were stained with the staining mix (Table S4, Panel 5, ICS) in permeabilization buffer overnight at 4°C in the dark. The cells were washed with FACS buffer and acquired with an Aurora Cytometer (Cytek). Data were analyzed with FlowJo and R (fig. S17A and B).

Total or MACS-enriched CD4^+^ T-blasts or HuT78 T-lymphoma cells (1 × 10^5^ cells per well) were left non-stimulated, were stimulated with polyclonal stimuli [PHA-M (Gibco, 1:100), ImmunoCult^™^ Human CD3/CD28/CD2 T Cell Activator (STEMCELL, 1:100), CytoStim (Miltenyi Biotec, 1:100), or Cell Stimulation Cocktail (eBioscience, 1:1000)], or were cocultured with target cells (1 × 10^5^ cells per well) and IL-2 (Roche, 10 ng/mL) with or without anti-CD19-anti-CD3 bispecific antibody (BPS Bioscience, 10 ng/mL) (fig. S16E and F). For allogeneic EBV-B cell coculture experiments, equal numbers of EBV-B cells from three healthy allogeneic donors were used after irradiation at 90 Gy to stop proliferation. For allogeneic PBMC coculture experiments, allogeneic PBMCs were first stained with CFSE (Cytek, 1:10000 in PBS) and then irradiated at 45 Gy to stop proliferation (fig. S16G). For RT-qPCR, the cells were pelleted by centrifugation and lysed in DNA/RNA Shield (Zymo Research, Cat: R1200–25) prediluted 1:1 with fresh lymphocyte medium; the resulting lysate was then stored at 4°C or −20°C until use. Total RNA was isolated with the Quick-RNA 96 Kit (Zymo Research, Cat: R1052) and reverse-transcribed with SuperScript IV Reverse Transcriptase (Invitrogen). Real-time PCR was performed with the cDNA and FAM-MGB TaqMan probes for *TBX21*, *GATA3,* and *RORC* (Thermo Fisher Scientific, s00203436_m1, Hs00231122_m1, and Hs01076122_m1/ Hs00172860_m1, respectively), as described above (fig. S17C). For intracellular flow cytometry, the cells were transferred to a V-bottom 96-well plate, washed once with PBS, and stained by incubation with Zombie NIR Fixable Viability dye (BioLegend, 1:1000 in PBS) for 15 minutes at 4°C in the dark. In the case of total T-blasts, the cells were stained with anti-CD4 and anti-CD8 mAbs for 30 minutes at 4°C in the dark. The cells were then washed with FACS buffer and fixed and permeabilized with the FOXP3/Transcription Factor Staining Buffer Kit (Cytek). The cells were stained by incubation overnight at 4°C in the dark with the following in permeabilization buffer: FcR blocking reagent (Miltenyi Biotec, 1:50), anti-CD3-APC (Tonbo Biosciences, Clone: UCHT1, 1:100), anti-CD3-BV421 (BioLegend, Clone: UCHT1, 1:100) or anti-CD3-V450 (BD Biosciences, Clone: UCTH1, 1:100), anti-T-bet-PE/Cy7 (BioLegend, Clone: 4B10, 1:500) or anti-T-bet-PE/Cy5 (eBioscience, Clone: 4B10, 1:500), anti-GATA3-PE/Vio 615 (Miltenyi Biotec, Cat: 130–109-161, Clone: REA174, 1:100) or anti-GATA3-APC (Miltenyi Biotec, Clone: REA174, 1:250), and anti-RORγT-PerCP/eFluor 710 (eBioscience, Clone: AFKJS-9, 1:500) antibodies. The cells were washed with FACS buffer and acquired with an Aurora Cytometer (Cytek) or an Attune NxT Flow Cytometer with the CytKick MAX Autosampler (Invitrogen). Data were analyzed with FlowJo and R.

### DNA methylation analysis

MACS-enriched CD4^+^ T-blasts from eight healthy donors, P2, P3, P4, a patient with X-linked SAP deficiency, a patient with T-bet deficiency (T-blasts were induced from PBMCs sampled on two different occasions as technical duplicates) (45), and three patients with RORγT deficiency (43) were analyzed in two batches. LY9-deficient CD4^+^ T-blasts from P2, P3, and P4 expressed lower levels of T-bet and RORγT than cells from healthy controls even after polyclonal stimulation (Fig. S18A and B), as observed for CD4^+^ αβ T lymphocytes in PBMCs (Fig. 5E–G).After 14 days of expansion following restimulation with ImmunoCult Human CD3/CD28/CD2 T-Cell Activator (STEMCELL, 1:100) and IL-2 (Roche, 10 ng/mL), genomic DNA was extracted with the PureLink Genomic DNA Mini Kit (Invitrogen, Cat: K182001) with proteinase K digestion. DNA was quantified with the Quant-iT PicoGreen dsDNA Assay Kit (Invitrogen, Cat: P7589). The DNA samples were deaminated with the EZ-96 DNA Methylation Kit (Zymo Research) according to Illumina’s recommended deamination protocol. Equal amounts of DNA for each sample were loaded onto the Illumina Infinium MethylationEPIC BeadChip array (850K; Batch 1) or the Illumina Infinium MethylationEPIC V2 BeadChip array (935K; Batch 2). The raw data were imported into R and normalized by the stratified quantile normalization method. Failed probes, for which the signals obtained for the methylated and unmethylated channels were similar to the background, were removed from further analyses (~1.5% and ~0.1% in Batches 1 and 2, respectively). Probes for the X and Y chromosomes were also removed from further analyses. EPIC V2 probe names were matched with those in EPIC V1. Unmatched probes were not used for further analyses. A total of 699,415 CpG sites were retained for subsequent analyses. Beta values for the top 10,000 probes were used for principal component analysis (PCA) after the elimination of batch effects with the *removeBatchEffect* function in limma. PCA revealed tight clustering by genotype, with PC1 higher in all the genotypes tested than in healthy controls (Fig. 5I). Differential methylation analysis was performed with limma (87) on M values, with batch and genotype as variables. CpG sites were considered to be LY9-, T-bet-, or RORγT-dependent when significantly (FDR-adjusted *P* value < 0.05 and |log2FC| > 1) more or less methylated in the LY9-, T-bet-, or RORγT-deficient cells than in control cells. T-bet- or RORγT-dependent CpG sites were defined based on differential regulation with respect to control cells in 1) T-bet- but not RORγT-deficient cells, 2) RORγT- but not T-bet-deficient cells, or 3) in both T-bet- and RORγT-deficient cells (hypermethylated CpG sites: *N* = 784, 109, 13; hypomethylated CpG sites: *N* = 1,181, 190, 11 for the three differential categories). LY9-dependent CpG sites were also identified (*N* = 536 and 1,283 for hyper- and hypomethylated CpG sites). Hyper- and hypo-methylated regions are denoted as “Hi” and “Lo” in Fig. 5J and K. Statistics for the overlap between LY9-dependent and T-bet- or RORγT-dependent CpG sites were calculated using Fisher’s exact tests. This analysis is similar to hypergeometric overrepresentation tests commonly used in geneset analysis in that we can estimate the statistical significance of the overlaps between two sets of CpG sites governed by two genotypes. If there is no correlation/overlap between the two sets, the odds ratio is expected to become 1. LY9-deficient cells had differential DNA methylation patterns that significantly overlapped with those in T-bet- and RORγT-deficient cells (Fig. 5J and K).

### Chromatin accessibility analysis

MACS-enriched CD4^+^ T-blasts from four healthy donors, P2, P4, a patient with T-bet deficiency (T-blasts were induced from PBMCs sampled on two different occasions as technical duplicates) (45), and three patients with RORγT deficiency (43) were analyzed in one batch. Omni-ATAC-seq library preparation was performed as previously described (45). Briefly, a two-sided size selection of amplified libraries was performed with AMPure XP beads (Beckman Coulter, Cat: A63880). Equal amounts of each sample were pooled and subjected to 50 bp paired-end sequencing on a NovaSeq 6000 sequencer. Bowtie2 (version 2.4.2) was used to map raw sequencing reads onto the reference genome (hg38) with default parameters. The resulting aligned reads were then subjected to peak calling with MACS2 (version 2.2.7.1) with a *q*-value threshold of 0.05. Downstream analyses were performed in R (version 4.2). We used ChIPseeker and GenomeInfoDb to annotate the peaks with their genomic locations and associated genes. The ChIPQC package was used to define a set of non-redundant chromatin accessibility regions across all samples. A total of 38,644 regions were identified. The reads aligned with each region were then counted with the *summarizeOverlaps* function, and the count matrix was analyzed with DESeq2 (90). Count data normalized by variance-stabilizing transformation were used for visualization in the PCA and heatmap analysis. Differential chromatin accessibility analysis was conducted according to the standard DESeq2 workflow. Chromatin regions were considered to be LY9-, T-bet-, or RORγT-dependent when significantly (FDR-adjusted *P* value < 0.05 and |log2FC| > 1) more or less accessible in the LY9-, T-bet-, or RORγT-deficient cells than in control cells. The numbers of differential chromatin accessibility peaks were as follows: 1) T-bet- but not RORγT-deficient cells (*N* = 788 and 1,106 for open and closed regions, respectively), 2) RORγT- but not T-bet-deficient cells (*N* = 38 and 32), or 3) in both T-bet- and RORγT-deficient cells (*N* = 112 and 20). There were also LY9-dependent chromatin regions (*N* = 322 and 195 for opened and closed regions, respectively). Chromatin regions with significantly higher or lower accessibility are denoted as “Hi” and “Lo” in Fig. 5K and J. Statistics for the overlap between LY9-dependent and T-bet- or RORγT-dependent chromatin regions were calculated using Fisher’s exact tests. LY9-deficient cells had differential chromatin accessibility patterns that significantly overlapped with those in T-bet- and RORγT-deficient cells (Fig. 5J and K).

### LY9 crosslinking assay in CD4^+^ T cells

Total or MACS-enriched CD4^+^ T-blasts or HuT78 cells were dispensed into a U-bottom 96-well plate (5 × 10^4^ to 2 × 10^5^ cells in 100 μL lymphocyte medium per well) and stimulated with in-house antibody-conjugated beads (cell:bead ratio=1:1) or Cell Stimulation Cocktail (eBioscience, 1:1000) at 37°C. For bulk RNASeq analysis, monensin and brefeldin A (Cytek, 1:1000 each) were added to prevent the paracrine effect of secreted cytokines. For pharmacological NFAT inhibition, MACS-enriched CD4^+^ T-blasts from P4 lentivirally transduced with EV or WT LY9 were dispensed in a U-bottom 96-well plate (5 × 10^4^ cells in 50 μL lymphocyte medium per well), and 25 μL lymphocyte medium supplemented with 3.57 μL PBS alone or 11R-VIVIT (Tocris, Cat: 5710, Batch: 5A; 10 μM) in PBS was added. One hour later, the cells were mixed with 25 μL lymphocyte medium supplemented with in-house antibody-conjugated beads (cell:bead ratio=1:1) and incubated for 24 hours at 37°C.

Supernatants were harvested and analyzed in a LEGENDplex assay. The delta increase and fold-changes in the levels of cytokines secreted (value obtained with anti-CD3 plus anti-LY9 antibodies subtracted from or divided by the value obtained with anti-CD3 antibody plus isotype control) are calculated as a readout to assess the effect of LY9 crosslinking. For intracellular cytokine staining, T-blasts were stimulated in the presence of monensin and brefeldin A (Cytek, 1:1000 each). The cells were stained by incubation with Zombie NIR Fixable Viability dye (BioLegend, 1:1000 in PBS) for 15 minutes at 4°C in the dark, surface-stained for ~ 1 hour at 4°C in the dark, fixed and permeabilized with the FOXP3/Transcription Factor Buffer Staining Kit (Cytek). The cells were then stained by overnight incubation with the following in permeabilization buffer at 4°C in the dark: FcR blocking reagent (Miltenyi Biotec, 1:50), anti-CD3-APC (Tonbo Biosciences, Clone: UCHT1, 1:100), anti-IFN-γ-PE/Dazzle 594 (BioLegend, Clone: 4S.B3, 1:500), anti-TNF-BV711 (BioLegend, Clone: MAb11, 1:500), anti-T-bet-PE/Cy7 (BioLegend, Clone: 4B10, 1:500), and anti-RORγT-PerCP/eFluor 710 (eBioscience, Clone: AFKJS-9, 1:500) antibodies. The cells were washed with FACS buffer and acquired with an Aurora Cytometer (Cytek) or an Attune NxT Flow Cytometer with the CytKick MAX Autosampler (Invitrogen). Data were analyzed with FlowJo and R. Fold-changes in the percentage of cytokine-producing cells (value obtained with anti-CD3 plus anti-LY9 antibodies divided by that obtained with anti-CD3 antibody plus isotype control) are calculated and normalized against the mean for the control cells in each batch.

For RT-qPCR and bulk RNASeq analysis, the cells were then pelleted and lysed in DNA/RNA Shield (Zymo Research, Cat: R1200–25) prediluted 1:1 with fresh lymphocyte medium and stored at 4°C or −20°C until use. Total RNA was isolated with the Quick-RNA 96 Kit (Zymo Research, Cat: R1052) and reverse-transcribed with SuperScript IV Reverse Transcriptase (Invitrogen). Real-time PCR was performed with the cDNA and FAM-MGB TaqMan probes for *IFNG* and *TNF* (Thermo Fisher Scientific, Hs00989291_m1 and Hs99999043_m1/Hs00174128_m1, respectively), as described above. *GUSB* was used as an endogenous control. Fold-changes in mRNA levels (value obtained with anti-CD3 plus anti-LY9 antibodies divided by value obtained with anti-CD3 antibody plus isotype control) are calculated as a readout to assess the effect of LY9 crosslinking. Bulk RNASeq was conducted as described in the previous section. Differential expression (DE) analysis was performed with DESeq2 (90). Geneset enrichment analysis (GSEA) was conducted with fgsea by projecting genes ranked according to fold-change in expression with effect-size shrinkage (91) onto the genesets retrieved from the MSigDB database (https://www.gsea-msigdb.org/gsea/msigdb/). For T_H_ gene signature analysis, log-normalized expression values were first retrieved from the public human leukocyte RNASeq dataset (GSE107011) (68) for T_H_1, T_H_2, T_H_17, and T_H_1* cells. Genes were considered to be differentially expressed between the T_H_ subsets if the difference in mean normalized expression was >= 1 or <= −1. The set of genes was then used for GSEA to test if any TH signature genes were significantly enriched in the LY9 crosslinking-induced genes. In the transcription factor motif enrichment analysis, only motifs for which significant enrichment was detected in either WT or YY are shown in the figure. For Hallmark genesets, only genesets for which significant enrichment was detected in both WT and YY are shown in the figure.

### Statistical analysis

All statistical analyses were performed in R v. 4 (http://www.R-project.org/) (96). The statistical significance of quantitative differences between groups was assessed in unpaired Wilcoxon’s rank-sum tests unless otherwise stated. False-discovery rate (FDR) adjustment was performed using the Benjamini and Hochberg method (97). *P* values below 0.05 were considered statistically significant.

## Supplementary Material

Data File 1

Data File 2

Table 4 Table S4. List of antibodies used for multi-color flow cytometry.

Reproducibility Checklist

Supp Materials Case report
fig. S1. Three unrelated cases of tuberculosis with biallelic *LY9* mutations.
fig. S2. Analysis of *LY9* alleles in an overexpression system.
fig. S3. Analysis of endogenous LY9 expression.
fig. S4. Presence of all lymphoid and myeloid leukocyte subsets in LY9-deficient individuals.
fig. S5. Normal serum antibody repertoire in LY9 deficiency.
fig. S6. Normal B-cell phenotypes in LY9 deficiency.
fig. S7. Analysis of the cellular responses of LY9-deficient leukocytes.
fig. S8. Analysis of cellular responses to mycobacterial stimulation *in vitro*.
fig. S9. Analysis of IFN-γ and TNF production by LY9-deficient lymphoid and myeloid leukocyte subsets.
fig. S10. Mechanistic analysis of cytokine production by CD4^+^ T lymphocytes.
fig. S11. Analysis of cytokine production by LY9-deficient antigen-specific CD4^+^ αβ T-cell clones.
fig. S12. Impairment of the ability of LY9-deficient CD4^+^ T lymphocytes to restrict the growth of *Listeria monocytogenes* in THP-1 phagocytes.
fig. S13. Quantitative analysis of LY9 expression in leukocyte subsets.
fig. S14. Transcriptomic analysis of LY9-deficient T_H_1* cells.
fig. S15. Analysis of naïve and memory CD4^+^ T lymphocytes in LY9 deficiency.
fig. S16. LY9 governs T-bet and RORγT expression in a CD4^+^ T-cell-intrinsic manner.
fig. S17. High levels of GATA3 expression in LY9-deficient CD4^+^ T lymphocytes.
fig. S18. Analysis of T-bet and RORγT expression in cultured LY9-deficient CD4^+^ T lymphocytes.
fig. S19. Analysis of T-bet and RORγT levels in LY9-deficient antigen-specific CD4^+^ αβ T-cell clones.
fig. S20. Correlation between IFN-γ-producing capacity and the levels of T-bet and RORγT in *M.tb*-specific CD4^+^ αβ T-cell clones.
fig. S21. Enhanced IFN-γ production through LY9 costimulation in CD4^+^ T lymphocytes.
fig. S22. Enhanced IFN-γ production through LY9 costimulation in T_H_1* cells.
fig. S23. Graphical summary.
Table S1. Enrichment of our in-house TB cohort in LY9 variants relative to healthy controls or patients with non-mycobacterial infectious diseases under various models.
Table S2. Laboratory test results for P1 at the age of two months.
Table S3. Laboratory test results for P4 at the age 16 years.

## Data Availability

The RNASeq, scRNASeq, and ATAC-Seq data have been deposited in the NCBI Sequence Read Archive (SRA) under the accession number PRJNA1185189. The methylation microarray data have been deposited in the Gene Expression Omnibus (GEO) repository under the accession number GSE281929. All raw data to generate figure panels are summarized in Data file S1. Raw data for the immunoblotting figures (Fig. 2B and fig. S2A) are provided in Data file S2. All raw and processed data and biological materials, including immortalized cell lines from patients, are available upon request from the corresponding authors under a material/data transfer agreement.
