# Supplementary figures and images for: Human LY9 governs CD4+ T-cell IFN-γ immunity to Mycobacterium tuberculosis
Source: Sci Immunol. Author manuscript; Available in PMC 2025 Jul 10. (PMC12242830; doi:10.1126/sciimmunol.ads7377)

Figure 2B


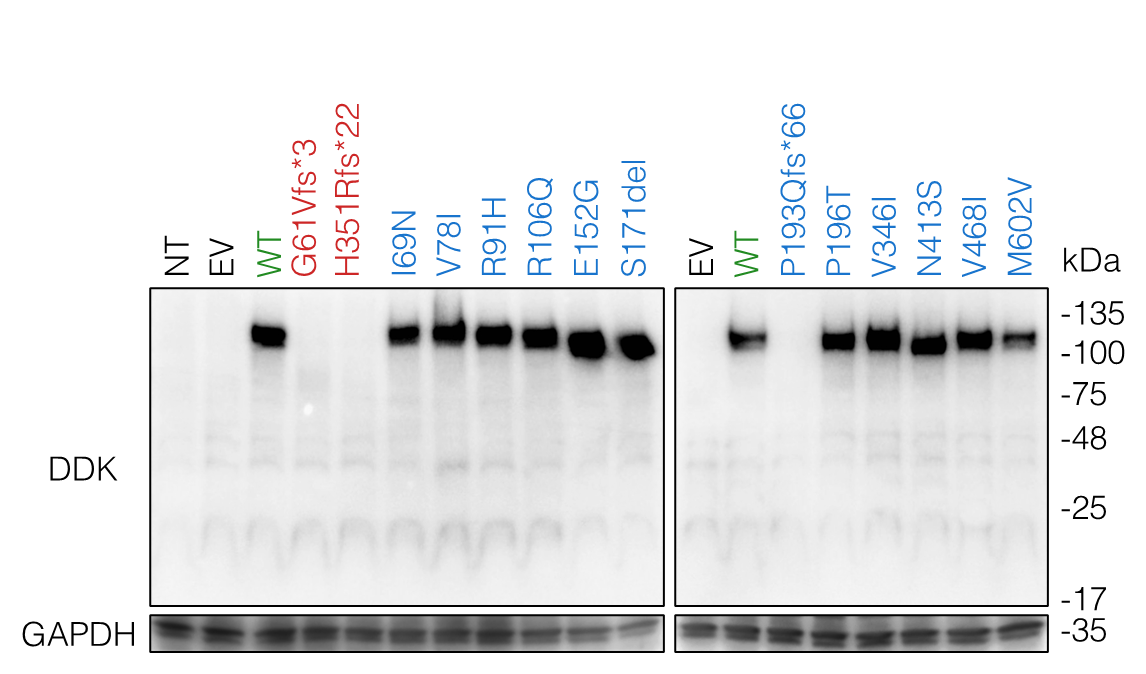


Anti-DDK


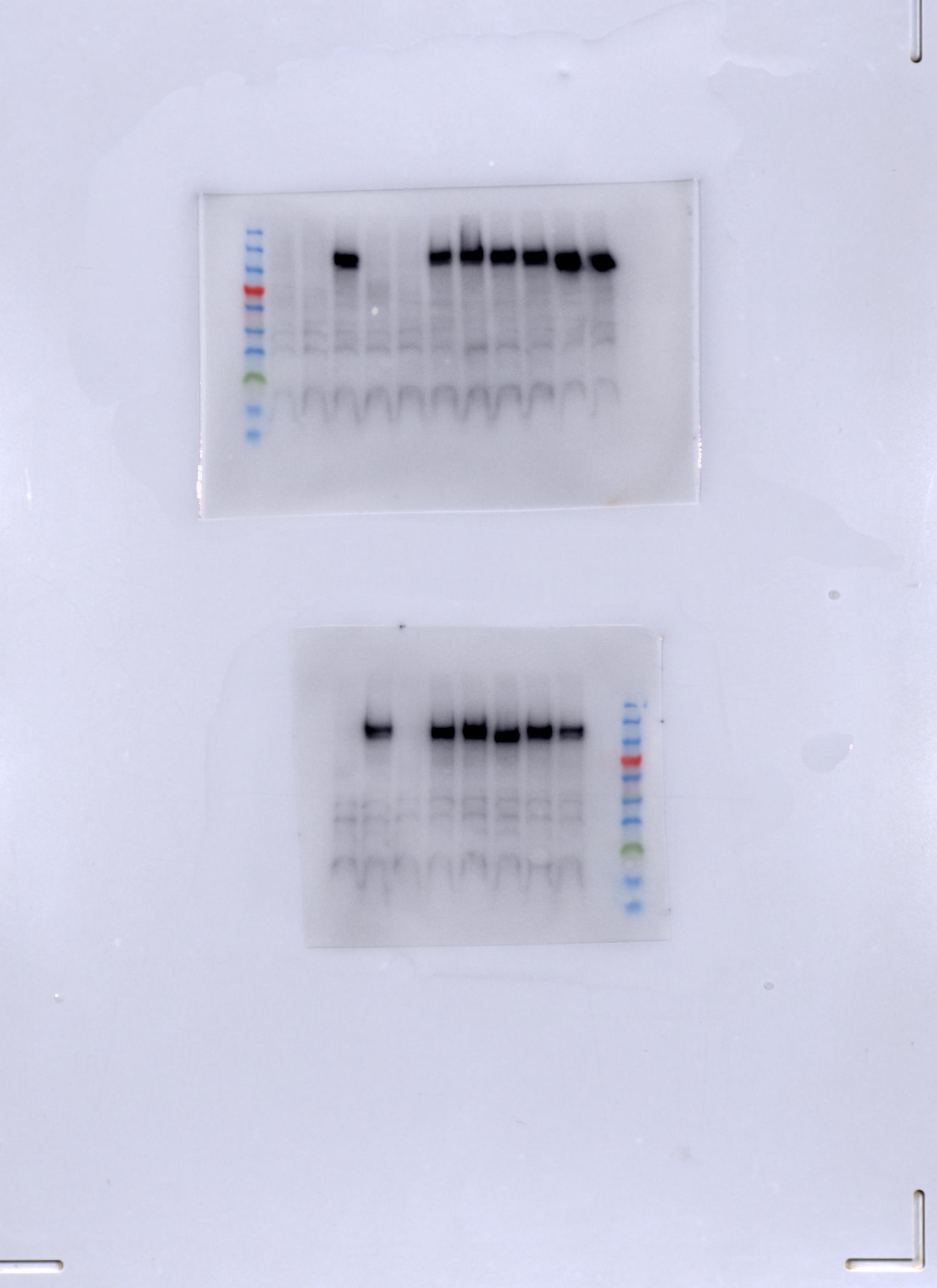


Anti-GAPDH


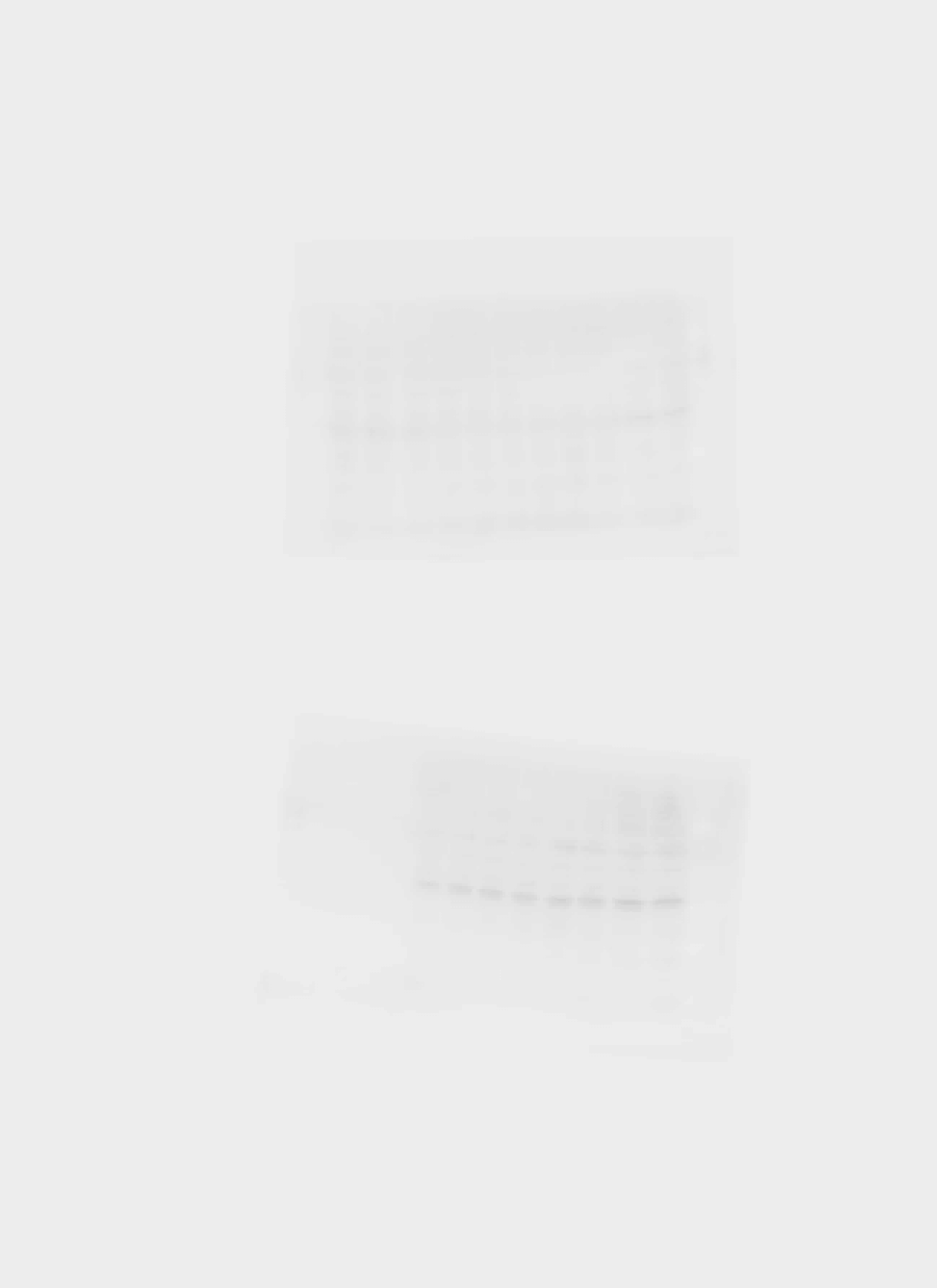


Figure S2


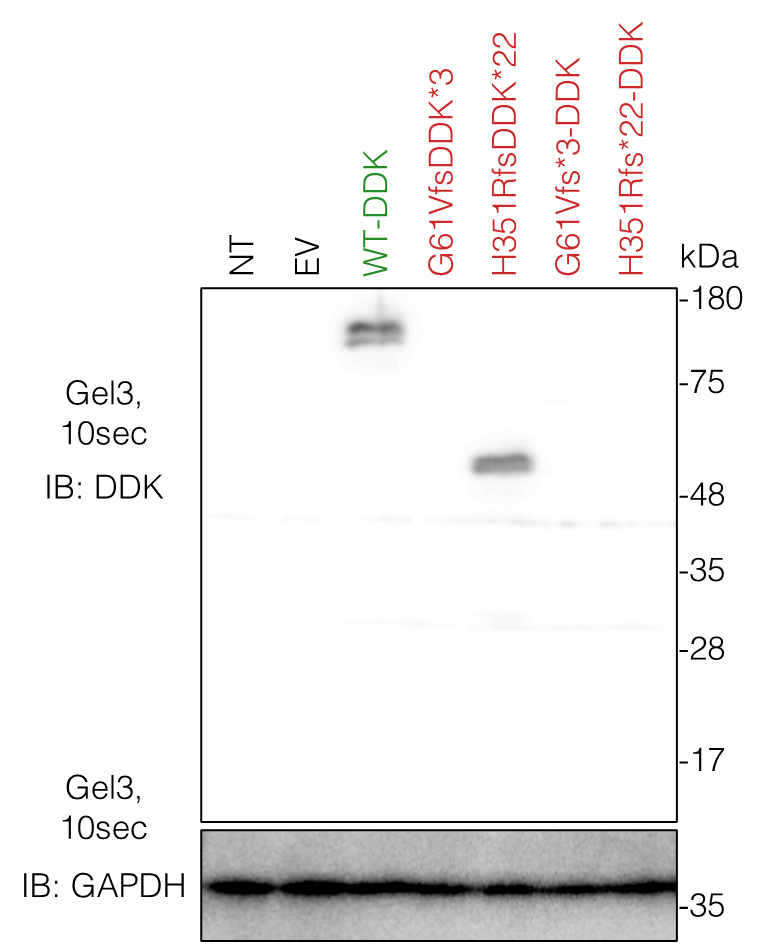


Anti-DDK


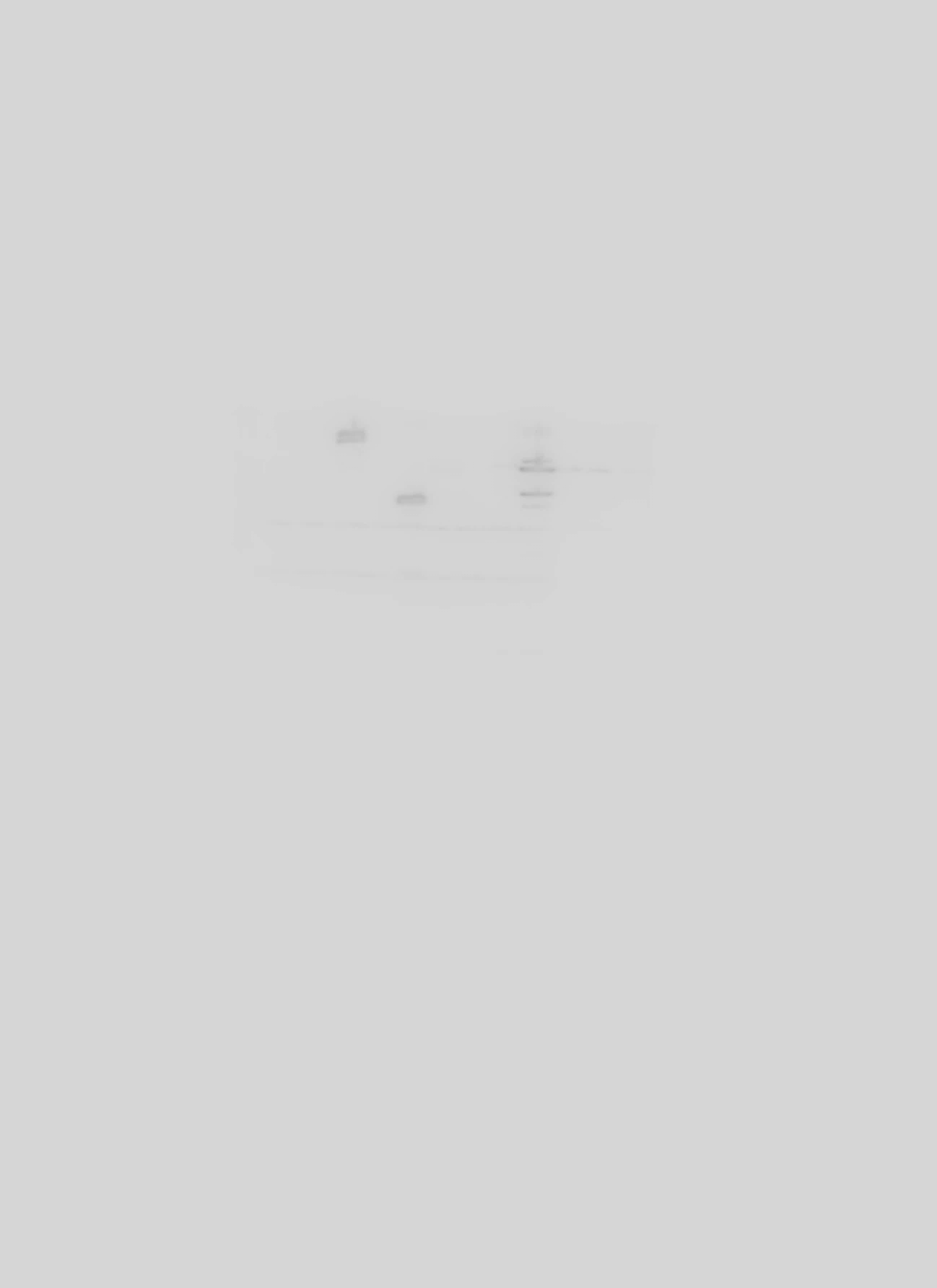


Anti-GAPDH


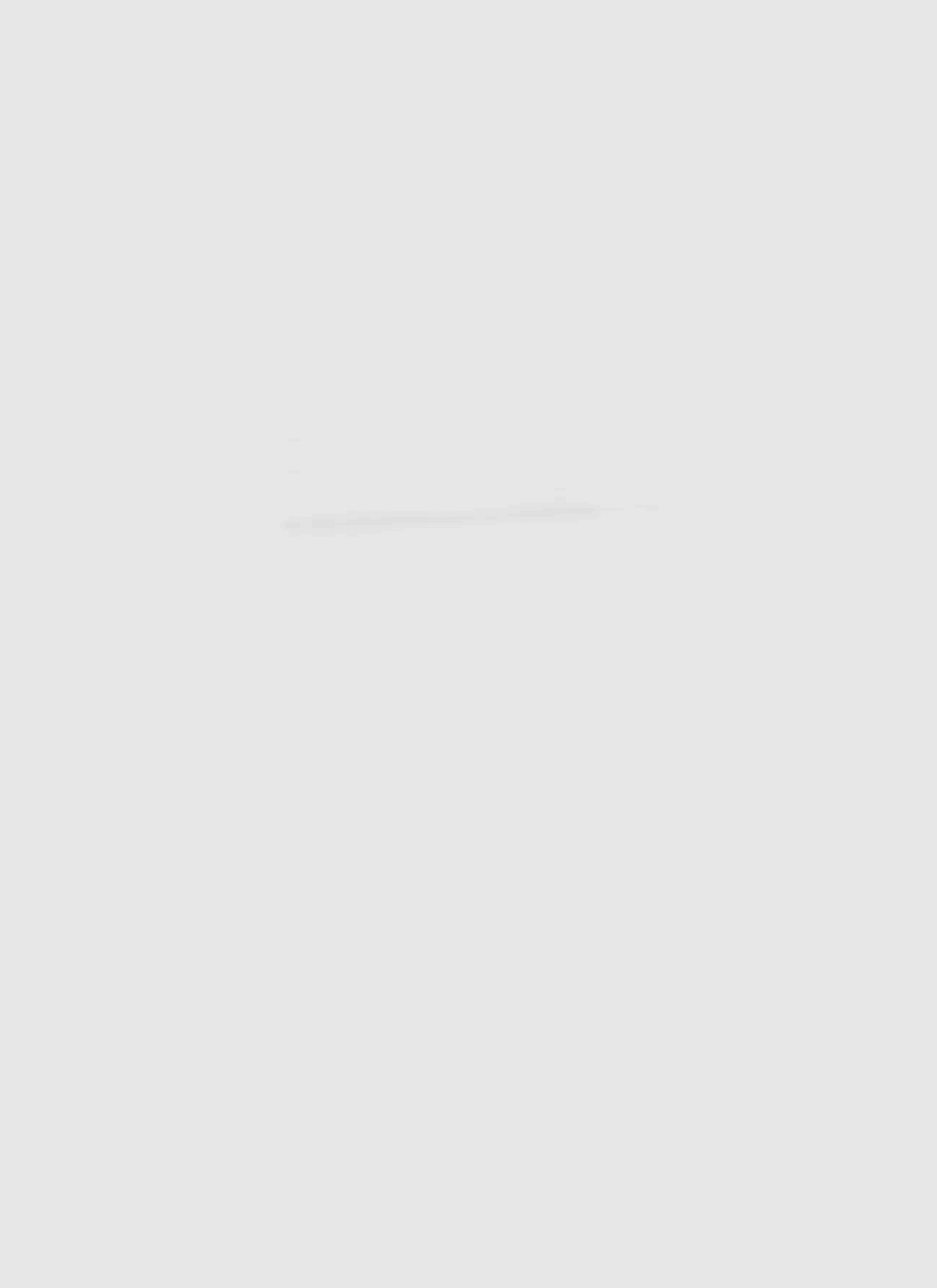

Supplement: Data File 2 [file NIHMS2092213-supplement-Data_File_2.docx]
