# Supplementary material for: Human LY9 governs CD4+ T-cell IFN-γ immunity to Mycobacterium tuberculosis
Source: Sci Immunol. Author manuscript; Available in PMC 2025 Jul 10. (PMC12242830; doi:10.1126/sciimmunol.ads7377)
Supplement: Reproducibility Checklist [file NIHMS2092213-supplement-Reproducibility_Checklist.pdf]

Materials Design Analysis Reporting (MDAR)
Checklist for Authors

The MDAR framework establishes a minimum set of requirements in transparent reporting applicable to studies in the life sciences (see Statement of Task: doi:10.31222/osf.io/9sm4x.). The MDAR checklist is a tool for authors, editors, and others seeking to adopt the MDAR framework for transparent reporting in manuscripts and other outputs. Please refer to the MDAR Elaboration Document for additional context for the MDAR framework.

For all that apply, please note where in the manuscript the required information is provided.

Materials:

Newly created materials	indicate where provided: page no/section/legend)	n/a
The manuscript includes a dedicated "materials availability statement" providing transparent disclosure about availability of newly created materials including details on how materials can be accessed and describing any restrictions on access.		n/a

Antibodies	indicate where provided: page no/section/legend)	n/a
For commercial reagents, provide supplier name, catalogue number and RRID, if available.	anti-CCR4-BUV615 (BD Biosciences, Cat: 613000, Clone: 1G1, 1:25<br>anti-CCR6-BV650 (BioLegend, Cat: 353426, Clone: G034E3, 1:25<br>anti-CCR6-BV711 (BioLegend, Cat: 353436, Clone: G034E3, 3:250<br>anti-CCR7-Alexa Fluor 647 (BioLegend, Cat: 353218, Clone: G043H7, 1:25<br>anti-CCR7-BV785 (BioLegend, Cat: 353230, Clone: G043H7, 1:40<br>anti-CD117-PE-Dazzle 594 (BioLegend, Cat: 313226, Clone: 104D2, 3:250<br>anti-CD11b-BUV563 (BD Biosciences, Cat: 741357, Clone: ICRF44, 1:100<br>anti-CD11c-BV480 (BD Biosciences, Cat: 566135, Clone: B-ly6, 1:40<br>anti-CD11c-BV510 (BioLegend, Cat: 301633, Clone: 3.9, 1:100<br>anti-CD123-BUV496 (BD Biosciences, Cat: 751836, Clone: 6H6, 1:100<br>anti-CD123-Super Bright 436 (Invitrogen, Cat: 62-1239-42, Clone: 6H6, 1:40<br>anti-CD127-APC-R700 (BD Biosciences, Cat: 565185, Clone: HIL-7R-M21, 1:50<br>anti-CD141-BB515 (BD Biosciences, Cat: 565084, Clone: 1A4, 1:40<br>anti-CD14-APC-Cy7 (BioLegend, Cat: 325620, Clone: HCD14, 1:100<br>anti-CD14-Spark Blue 550 (BioLegend, Cat: 367148, Clone: 63D3, 1:40<br>anti-CD14-Spark NIR 685 (BioLegend, Cat: 367149, Clone: 63D3, 1:100<br>anti-CD161-BV650 (BD Biosciences, Cat: 563864, Clone: DX12, 1:25<br>anti-CD16-APC (Tonbo Biosciences, Cat: 20-0166-T100, Clone: 3G8, 1:100<br>anti-CD16-BUV496 (BD Biosciences, Cat: 612944, Clone: 3G8, 3:500<br>anti-CD16-BUV496 (BioLegend, Cat: 302054, Clone: 3G8, 1:100<br>anti-CD16-PE/Dazzle 594 (BioLegend, Cat: 302054, Clone: 3G8, 1:100<br>anti-CD16-PE/Fire 640 (BioLegend, Cat: 302068, Clone: 3G8, 1:100<br>anti-CD19-Spark NIR 685 (BioLegend, Cat: 302270, Clone: HIB19, 1:200<br>anti-CD19-Super Bright 645 (eBioscience, Cat: 64-0198-41, Clone: SJ25C1, 1:100<br>anti-CD1c-Alexa Fluor 647 (BioLegend, Cat: 331510,	

	Clone: L161, 1:50<br>anti-CD209-PE/Cy7 (BioLegend, Cat: 330114, Clone: 9E9A8, 1:25<br>anti-CD20-Pacific Orange (Invitrogen, Cat: MHCD2030, Clone: HI47, 1:50<br>anti-CD24-PE-Alexa Fluor 610 (Invitrogen, Cat: MHCD2422, Clone: SN3, 1:25<br>anti-CD25-PE-Alexa Fluor 700 (Invitrogen, Cat: MHCD2524, Clone: 3G10, 1:25<br>anti-CD27-APC/H7 (BD Biosciences, Cat: 560222, Clone: M-T271, 1:50<br>anti-CD33-BV570 (BioLegend, Cat: 303417, Clone: WM53, 3:250<br>anti-CD38-APC-Fire 810 (BioLegend, Cat: 356644, Clone: HB-7, 3:100<br>anti-CD3-BV421 (BioLegend, Cat: 300434, Clone: UCHT1, 1:100<br>anti-CD3-Pacific Blue (BioLegend, Cat: 344824, Clone: SK7, 3:250<br>anti-CD3-V450 (BD Biosciences, Cat: 560366, Clone: UCHT1, 1:100<br>anti-CD45-Alexa Fluor 532 (eBioscience, Cat: 58-0459-42, Clone: HI30, 1:100<br>anti-CD45-BV510 (BD Biosciences, Cat: 563204, Clone: HI30, 3:250<br>anti-CD45RA-BUV395 (BD Biosciences, Cat: 740315, Clone: 5H9, 3:250<br>anti-CD4-BUV395 (BD Biosciences, Cat: 563552, Clone: SK3, 1:100<br>anti-CD4-BUV563 (BD Biosciences, Cat: 612913, Clone: SK3, 1:200<br>anti-CD4-redFluor 710 (Tonbo Biosciences, Cat: 80-0048-T100, Clone: OKT4, 1:25<br>anti-CD56-Alexa Fluor 488 (BD Biosciences, Cat: 557699, Clone: B159, 1:100<br>anti-CD56-BUV395 (BD Biosciences, Cat: 563555, Clone: NCAM16.2, 1:100<br>anti-CD56-BUV737 (BD Biosciences, Cat: 612767, Clone: NCAM16.2, 3:250<br>anti-CD56-cFluor BYG750 (Cytek, Cat: SKU R7-20284, Clone: TULY56, 1:100<br>anti-CD57-FITC (BD Biosciences, Cat: 347393, Clone: HNK-1, 3:250<br>anti-CD66b-FITC (BioLegend, Cat: 305104, Clone: G10F5, 1:100<br>anti-CD8-BUV737 (BD Biosciences, Cat: 612755, Clone: SK1, 1:450<br>anti-CD8-BUV805 (BD Biosciences, Cat: 612889, Clone: SK1, 3:250<br>anti-CD8-BV711 (BioLegend, Cat: 301044, Clone: RPA-T8, 1:100<br>anti-CRTH2-Biotin (Invitrogen, Cat: 13-2949-82, Clone: BM16, 1:50<br>anti-CXCR3-BUV737 (BD Biosciences, Cat: 746895, Clone: 1C6, 1:25<br>anti-CXCR3-BV750 (BD Biosciences, Cat: 746895, Clone: 1C6, 1:20<br>anti-GATA3-APC (Miltenyi Biotec, Cat: 130-128-583, Clone: REA174, 1:250<br>anti-GATA3-PE/Vio 615 (Miltenyi Biotec, Cat: 130-109-161, Clone: REA174, 1:100<br>anti-gdTCR-BB700 (BD Biosciences, Cat: 745944, Clone: 11F2, 1:25<br>anti-gdTCR-BUV661 (BD Biosciences, Cat: 750019, Clone:	

	11F2, 1:50<br>anti-gdTCR-PE/Cy7 (BioLegend, Cat: 306720, Clone: IP26, 1:100<br>anti-Granzyme B-Pacific Blue (BioLegend, Clone: GB11, Cat: 515407<br>anti-HLA-DR-APC/Fire 810 (BioLegend, Cat: 307674, Clone: L243, 1:100<br>anti-IFN-g-BUV395 (BD Biosciences, Clone: B27, Cat: 563563<br>anti-IFN-g-PE/Dazzle 594 (BioLegend, Cat: 502546, Clone: 43.B3, 1:100<br>anti-IL-17A-BV605 (BioLegend, Clone: BL168, Cat: 512325<br>anti-IL-22-BUV737 (eBioscience, Clone: 22URTI, Cat: 367-7229-42<br>anti-IL-4-BV711 (BD Biosciences, Clone: MP4-25D2, Cat: 564112<br>anti-iNKT-BV480 (BD Biosciences, Cat: 746788, Clone: 6B11, 1:100<br>anti-iNKT-BV605 (BD Biosciences, Cat: 743999, Clone: 6B11, 1:25<br>anti-LY9-APC (eBioscience, Cat: 17-2299-42, Clone: HLy9.25, 1:100<br>anti-LY9-PE (BioLegend, Cat: 326108, Clone: HLy-9.1.25, 1:100<br>anti-LY9-PE (BioLegend, Clone: HLy-9.1.25, Cat: 326108, 1:100<br>anti-phospho-ERK1/2-Alexa Fluor 647 (BioLegend, Cat: 369504, Clone: 6B8B69, 1:20<br>anti-RORgT-PE (BD Biosciences, Cat: 563081, Clone: Q21-559, 1:100<br>anti-RORgT-PE (BD Biosciences, Cat: 563081, Clone: Q21-559, 1:50<br>anti-T-bet-APC (Miltenyi Biotec, Clone REA102, Cat: 130-119-821<br>anti-T-bet-PE/Cy5 (eBioscience, Cat: 15-5825-82, Clone: 4B10, 1:500<br>anti-T-bet-PE/Cy7 (BioLegend, Cat: 644824, Clone: 4B10, 1:1000<br>anti-TNF-BV785 (BioLegend, Clone: MAb11, Cat: 502947<br>anti-TNF-PE/Vio 770 (Miltenyi Biotec, Cat: 130-127-550, Clone: REA656, 1:1000<br>anti-Va7.2-Alexa Fluor 700 (BioLegend, Cat: 351728, Clone: 3C10, 1:50<br>anti-Va7.2-PerCP/Cy5.5 (BioLegend, Cat: 351710, Clone: 3C10, 1:40<br>anti-Vb11-APC/Vio 770 (Miltenyi Biotec, Cat: 130-127-292, Clone: REA559, 1:100<br>anti-Vb11-PE (Miltenyi Biotec, Cat: 130-123-561, Clone: REA559, 3:500<br>anti-Vd1-PerCP/Vio 700 (Miltenyi Biotec, Cat: 130-120-441, Clone: REA173, 1:100<br>anti-Vd1-VioBright B515 (Miltenyi Biotec, Cat: 130-126-973, Clone: REA173, 1:100<br>anti-Vd2-BUV805 (BD Biosciences, Cat: 748580, Clone: B6, 1:450<br>anti-Vd2-PerCP (BioLegend, Cat: 331410, Clone: B6, 3:500<br>Streptavidin-PE-Cy5 (BioLegend, Cat: 405205, 1:3000<br>Mouse IgG1 isotype control-PE (eBioscience, Cat: 12-4714-42, Clone: P3.6.2.8.1<br>FcR blocking reagent (Miltenyi Biotec, Cat: 130-059-901, 1:50<br>CFSE (Invitrogen, Cat: C1157	

	Ghost Dye UV450 (Cytek, Cat: SKU 13-0868-T100, 1:1000 in PBS<br>Ghost Dye Violet 510 (Cytek, Cat: SKU 13-0870-T100, 1:1000 in PBS<br>LIVE/DEAD Fixable Aqua (Invitrogen, Cat: L34957, 1:1000 in PBS<br>LIVE/DEAD Fixable Blue (Invitrogen, Cat: L23105, 1:800 in PBS<br>Zombie NIR Fixable Viability dye (BioLegend, Cat: 423105, 1:1000 in PBS<br>Monensin (Cytek, Cat: SKU TNB-4505-L001, 1:1000<br>Brefeldin A (Cytek, Cat: SKU TNB-4506-L001, 1:1000<br>Brilliant Stain Buffer Plus (BD Biosciences, Cat: 566385, 1:5<br>HRP-conjugated anti-DYKDDDDK Tag antibody (BioLegend, Cat: 637312, Clone: L5, 1:2000<br>HRP-conjugated anti-GAPDH antibody (Santa Cruz, Cat: sc-47724 HRP, Clone: 0411, 1:5000<br>Dynabeads M-450 Epoxy (Invitrogen, Cat: 14011<br>Ultra-LEAF purified anti-CD3 antibody (BioLegend, Cat: 317236, Clone: OKT3<br>Ultra-LEAF purified anti-human LY9 antibody (BioLegend, Cat: 326109, Clone: HLy-9.1.25<br>Ultra-LEAF purified mouse IgG1 isotype control (BioLegend, Cat: 400166, Clone: MOPC-21<br>CD4 MicroBeads (Miltenyi Biotec, Cat: 130-045-101<br>anti-CD19-anti-CD3 bispecific antibody (BPS Bioscience, Cat: 100441-1<br>CytoStim (Miltenyi Biotec, Cat: 130-092-172, 1:100<br>Dynabeads Human T-Activator CD3/CD28 (Thermo Fisher Scientific, Cat: 11161D, Bead:Cell=1:1<br>ImmunoCult Human CD3/CD28/CD2 T-Cell Activator (STEMCELL Technologies, Cat: 10970; 1:100<br>ImmunoCult XF T-Cell Expansion Medium (STEMCELL Technologies, Cat: 10981<br>Recombinant human interleukin 2 (rhIL-2; Roche, Cat: 11147528001<br>Phytohemagglutinin-M (PHA; Gibco, Cat: 10576015<br>PMA (Sigma-Aldrich, Cat: P8139-1MG<br>Cell Stimulation Cocktail (eBioscience, Cat: 00-4970-93; 1:1000<br>MEGACD40L (ENZO, Cat: ALX-522-110-C010<br>IL-21 (PeproTech, Cat: 200-21<br>Gamma-irradiated M.tb H37Rv whole-cell lysate (BEI Resources, NIAID, NIH, Cat: NR-14822<br>heat-killed M.tb (HKMTb; InVivoGen, Cat: tlrl-hkmt-5<br>Listeria monocytogenes (1/2a) stably expressing GFP (Microbiologics, Cat: 01249UV-V, Lot: 1249-13-1<br>Salmonella enterica serotype Minnesota (LPS; Sigma-Aldrich, Cat: L4641	

DNA and RNA sequences	indicate where provided: page no/section/legend)	n/a
**Short novel DNA or RNA including primers, probes:** Sequences should be included or deposited in a public repository.		n/a

Cell materials	indicate where provided: page no/section/legend	n/a
**Cell lines:** Provide species information, strain. Provide accession number in repository **OR** supplier name, catalog number, clone number, **OR** RRID.	HEK293T, HuT78, Raji, and THP-1 cell lines were purchased from ATCC. Details are provided in the Materials and Methods.	
**Primary cultures:** Provide species, strain, sex of origin, genetic modification status.		n/a

Experimental animals	indicate where provided: page no/section/legend)	n/a
**Laboratory animals or Model organisms:** Provide species, strain, sex, age, genetic modification status. Provide accession number in repository **OR** supplier name, catalog number, clone number, **OR** RRID.		n/a
**Animal observed in or captured from the field:** Provide species, sex, and age where possible.		n/a

Plants and microbes	indicate where provided: page no/section/legend)	n/a
**Plants:** provide species and strain, ecotype and cultivar where relevant, unique accession number if available, and source (including location for collected wild specimens).		n/a
**Microbes:** provide species and strain, unique accession number if available, and source.		n/a

Human research participants	indicate where provided: page no/section/legend) or state if these demographics were not collected	n/a
If collected and within the bounds of privacy constraints report on age, sex and gender or ethnicity for all study participants.	P1 is a 3mo Moroccan female. P2 is a 29-yo Moroccan male (patternal uncle of P1). P3 is a 40-yo Moroccan male. P4 is a 14-yo Turkish male.	

Design:

Study protocol	indicate where provided: page no/section/legend)	n/a
If study protocol has been pre-registered, provide DOI. For clinical trials, provide the trial registration number **OR** cite DOI.		n/a

Laboratory protocol	indicate where provided: page no/section/legend)	n/a
Provide DOI **OR** other citation details if detailed step-by-step protocols are available.		n/a

Experimental study design (statistics details)		
**For in vivo studies:** State whether and how the following have been done	**indicate where provided: page no/section/legend. If it could have been done, but was not, write not done**	**n/a**
Sample size determination		n/a
Randomisation		n/a
Blinding		n/a
Inclusion/exclusion criteria		n/a

Sample definition and in-laboratory replication	indicate where provided: page no/section/legend	n/a
State number of times the experiment was replicated in laboratory.	The number of repetitions is described in each Figure legend.	
Define whether data describe technical or biological replicates.	The nature of replicates is described in each Figure legend.	

Ethics	indicate where provided: page no/section/legend	n/a
**Studies involving human participants:** State details of authority granting ethics approval (IRB or equivalent committee(s), provide reference number for approval.	All individuals were recruited according to protocols approved by local IRBs. Written informed consent was obtained in the country of residence of each patient. Experiments were conducted in France and the United States in accordance with local regulations and with the approval of the IRB of the Institut National de la Santé et de la Recherche Médicale and Rockefeller University, respectively. Approval was obtained from the French Ethics Committee (Comité de Protection des Personnes), the French National Agency for Medicine and Health Product Safety, the Institut National de la Santé et de la Recherche Médicale in Paris, France (protocol no. C10-16; ID-RCB 2010-A00650-39), and the Rockefeller University IRB in New York, New York, USA (protocol no. JCA-0699).	
**Studies involving experimental animals:** State details of authority granting ethics approval (IRB or equivalent committee(s), provide reference number for approval.		n/a
**Studies involving specimen and field samples:** State if relevant permits obtained, provide details of authority approving study; if none were required, explain why.		n/a

Dual Use Research of Concern (DURC)	indicate where provided: page no/section/legend	n/a

If study is subject to dual use research of concern regulations, state the authority granting approval and reference number for the regulatory approval.		n/<br>a

Analysis:

Attrition	indicate where provided: page no/section/legend	n/a
Describe whether exclusion criteria were preestablished. Report if sample or data points were omitted from analysis. If yes report if this was due to attrition or intentional exclusion and provide justification.		n/a

Statistics	indicate where provided: page no/section/legend	n/a
Describe statistical tests used and justify choice of tests.	Statistical tests were described in each Figure legend.	

Data availability	indicate where provided: page no/section/legend	n/a
For newly created and reused datasets, the manuscript includes a data availability statement that provides details for access or notes restrictions on access.	The RNASeq, scRNASeq, and ATAC-Seq data have been deposited in the NCBI Sequence Read Archive (SRA) under the accession number PRJNA1185189. The methylation microarray data have been deposited in the Gene Expression Omnibus (GEO) repository under the accession number GSE281929.	
If newly created datasets are publicly available, provide accession number in repository **OR** DOI **OR** URL and licensing details where available.	The RNASeq, scRNASeq, and ATAC-Seq data have been deposited in the NCBI Sequence Read Archive (SRA) under the accession number PRJNA1185189. The methylation microarray data have been deposited in the Gene Expression Omnibus (GEO) repository under the accession number GSE281929.	
If reused data is publicly available provide accession number in repository **OR** DOI **OR** URL, **OR** citation.		n/a

Code availability	indicate where provided: page no/section/legend	n/a
For all newly generated custom computer code/software/mathematical algorithm or re-used code essential for replicating the main findings of the study, the manuscript includes a data availability statement that provides details for access or notes restrictions.		n/a
If newly generated code is publicly available, provide accession number in repository, **OR** DOI **OR** URL and licensing details where available. State any restrictions on code availability or accessibility.		n/a
If reused code is publicly available provide accession number in repository **OR** DOI **OR** URL, **OR** citation.		n/a

Reporting

MDAR framework recommends adoption of discipline-specific guidelines, established and endorsed through community initiatives. Journals have their own policy about requiring specific guidelines and recommendations to complement MDAR.

Adherence to community standards	indicate where provided: page no/section/legend	n/a
State if relevant guidelines (e.g., ICMJE, MIBBI, ARRIVE) have been followed, and whether a checklist (e.g., CONSORT, PRISMA, ARRIVE) is provided with the manuscript.		n/a
