# Supplementary material for: Human LY9 governs CD4+ T-cell IFN-γ immunity to Mycobacterium tuberculosis
Source: Sci Immunol. Author manuscript; Available in PMC 2025 Jul 10. (PMC12242830; doi:10.1126/sciimmunol.ads7377)
Supplement: Supp Materials — Case report fig. S1. Three unrelated cases of tuberculosis with biallelic LY9 mutations. fig. S2. Analysis of LY9 alleles in an overexpression system. fig. S3. Analysis of endogenous LY9 expression. fig. S4. Presence of all lymphoid and myeloid leukocyte subsets in LY9-deficient individuals. fig. S5. Normal serum antibody repertoire in LY9 deficiency. fig. S6. Normal B-cell phenotypes in LY9 deficiency. fig. S7. Analysis of the cellular responses of LY9-deficient leukocytes. fig. S8. Analysis of cellular responses to mycobacterial stimulation in vitro. fig. S9. Analysis of IFN-γ and TNF production by LY9-deficient lymphoid and myeloid leukocyte subsets. fig. S10. Mechanistic analysis of cytokine production by CD4+ T lymphocytes. fig. S11. Analysis of cytokine production by LY9-deficient antigen-specific CD4+ αβ T-cell clones. fig. S12. Impairment of the ability of LY9-deficient CD4+ T lymphocytes to restrict the growth of Listeria monocytogenes in THP-1 phagocytes. fig. S13. Quantitative analysis of LY9 expression in leukocyte subsets. fig. S14. Transcriptomic analysis of LY9-deficient TH1* cells. fig. S15. Analysis of naïve and memory CD4+ T lymphocytes in LY9 deficiency. fig. S16. LY9 governs T-bet and RORγT expression in a CD4+ T-cell-intrinsic manner. fig. S17. High levels of GATA3 expression in LY9-deficient CD4+ T lymphocytes. fig. S18. Analysis of T-bet and RORγT expression in cultured LY9-deficient CD4+ T lymphocytes. fig. S19. Analysis of T-bet and RORγT levels in LY9-deficient antigen-specific CD4+ αβ T-cell clones. fig. S20. Correlation between IFN-γ-producing capacity and the levels of T-bet and RORγT in M.tb-specific CD4+ αβ T-cell clones. fig. S21. Enhanced IFN-γ production through LY9 costimulation in CD4+ T lymphocytes. fig. S22. Enhanced IFN-γ production through LY9 costimulation in TH1* cells. fig. S23. Graphical summary. Table S1. Enrichment of our in-house TB cohort in LY9 variants relative to healthy controls or patients with non-mycobacterial i [file NIHMS2092213-supplement-Supp_Materials.pdf]

Science Immunology
AAAS

Supplementary Materials for

Human LY9 governs CD4$^+$ T cell IFN-γ immunity to *Mycobacterium tuberculosis*

Masato Ogishi *et al.*

Corresponding author: Masato Ogishi, mogishi@rockefeller.edu;
Stéphanie Boisson-Dupuis, stbo603@rockefeller.edu; Jean-Laurent Casanova, casanova@rockefeller.edu

Sci. Immunol. **10**, eads7377 (2025)
DOI: 10.1126/sciimmunol.ads7377

The PDF file includes:

Case Reports
Figs. S1 to S23
Tables S1 to S3

Other Supplementary Material for this manuscript includes the following:

Table S4
Data files S1 and S2
MDAR Reproducibility Checklist

Case Reports

P1 was a three-month-old Moroccan girl with first-degree consanguineous parents. She was delivered via the vaginal route and had no neonatal problems. She was breastfed for one month and then fed with formula milk. She was vaccinated according to local vaccination schedules, including BCG vaccination. No adverse events, including BCG-itis, were noted following vaccination. At the age of two months, P1 presented with a persistent cough with occasional hemoptysis of more than 20 days' duration. At presentation, she measured 59 cm and weighed 4 kg. Physical examination revealed bilateral rales. No palpable mass or hepatosplenomegaly was noted. Serological tests for HIV-1 and 2 were negative. A thoracic computed tomography (CT) scan revealed bilateral lobar consolidation with atelectasis predominantly in the left lung. A clinical diagnosis of pulmonary TB was made based on the radiological findings and the endemic nature of TB in the region in which the family lived. Treatment with anti-tuberculous drugs (ethambutol, rifampicin, isoniazid, and pyrazinamide) was initiated. After three months of anti-tuberculous treatment, the patient's clinical status improved, and she was discharged from the hospital. However, 4.5 months after the initiation of anti-tuberculous treatment, the patient experienced respiratory distress and fever and was readmitted to the hospital. Unfortunately, she died at the age of seven months. Her family history was unremarkable, with none of her relatives having a history suggestive of TB. Aside from TB, dermatological examinations on presentation revealed crusty lesions on an erythematous background on the lower leg and bullous lesions on the left thumb, consistent with epidermolysis bullosa. Whole-exome sequencing identified a homozygous stop-gain variant of *PLEC*, suggesting that P1 also had AR plectin deficiency (*98*). P1 received only non-invasive, conservative care for her dermatological conditions.

P2 is a paternal uncle of P1. Genotyping of the *LY9* allele (c.182del) by Sanger sequencing in relatives of P1 revealed that P2 was also homozygous for the c.182del allele. P2 was healthy at genotyping and had no remarkable antecedents, such as unusually severe infections, in his medical history. He had been vaccinated with BCG, with no adverse effects. His chest X ray was clear, and a

Quantiferon-TB Gold Plus test was negative (TB1: 0.03 IU/mL, TB2: 0.00 IU/mL, mitogen: >10.00 IU/mL, Nul: 0.05 IU/mL) at the age of 29 years.

P3 is a 28-year-old Moroccan man who developed pulmonary TB. His sputum smear was positive for acid-fast bacilli, and a Quantiferon-TB Gold Plus test was positive (TB1: 3.78 IU/mL, TB2: 3.60 IU/mL, mitogen: >10.00 IU/mL, Nul: 1.31 IU/mL). He had been vaccinated with BCG, with no adverse effects. All his relatives were healthy, with unremarkable medical histories.

P4 is a 14-year-old boy from a consanguineous Turkish family. He has suffered from asthma since the age of six years (controlled with beta-agonists). He was fully vaccinated, including BCG vaccination, with no adverse events. He was referred to the hospital due to an abnormal finding on a chest X ray that persisted over a period of one month. A posteroanterior and lateral chest X ray revealed mediastinal widening and right hilar lymph node enlargement. Thoracic contrasted CT scan revealed right upper mediastinal lymphadenopathy with calcification. Abdominal ultrasound results were unremarkable. A tuberculin skin test yielded strongly positive results (induration of 15 mm). IGRA was not performed. The patient's gastric fluid tested negative for acid-fast bacilli on microscopy, and a mycobacterial culture was negative. Following the clinical and radiological diagnosis of mediastinal tuberculous lymphadenitis, the patient was treated with four anti-TB drugs (isoniazid, rifampicin, pyrazinamide, and ethambutol) for two months and then with two drugs (isoniazid and rifampicin) for four months. The mediastinal widening was no longer visible on the follow-up chest X ray obtained after six months of treatment. The patient was well after treatment and did not experience any recurrence during the routine follow-up.

Supplementary Figures

Figure S1

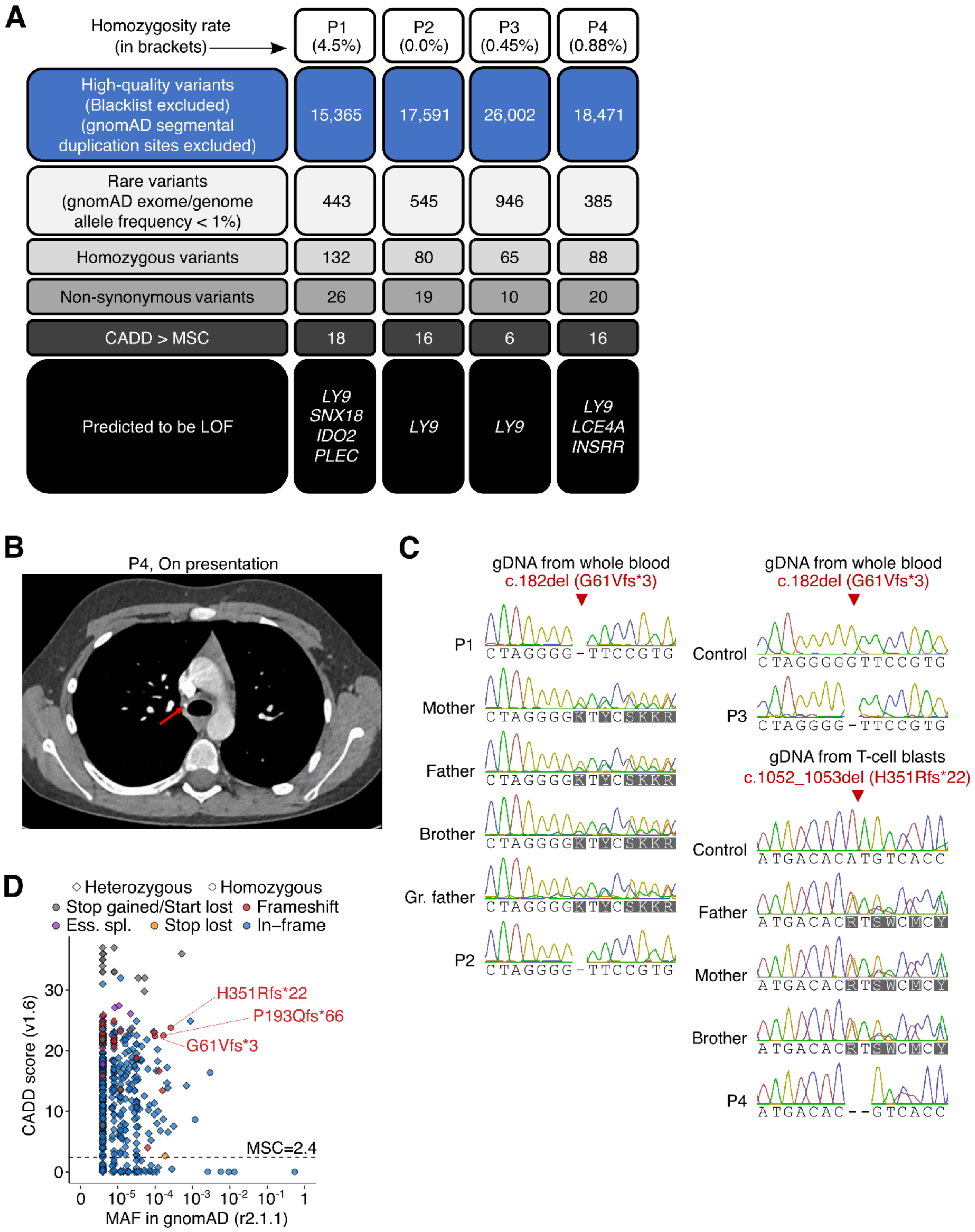

Figure S1. Three unrelated cases of tuberculosis with biallelic *LY9* mutations. (A) Whole-exome sequencing (WES) variant filtering strategy. Biallelic non-synonymous (missense, in-frame, frameshift, stop-gained, and essential splicing site) variants were further filtered based on their minor allele frequencies (MAFs) and combined annotation-dependent depletion (CADD) scores. MSC, mutation significance cutoff; LOF, loss-of-function. (B) A thoracic computed tomography scan for P4 on presentation, showing a mediastinal calcification (arrow). (C) Sanger sequencing chromatograms for the *LY9* region harboring the corresponding mutations. (D) Population genetics of *LY9*. MAFs and CADD scores for all non-synonymous variants reported in the gnomAD database are shown.

Figure S2

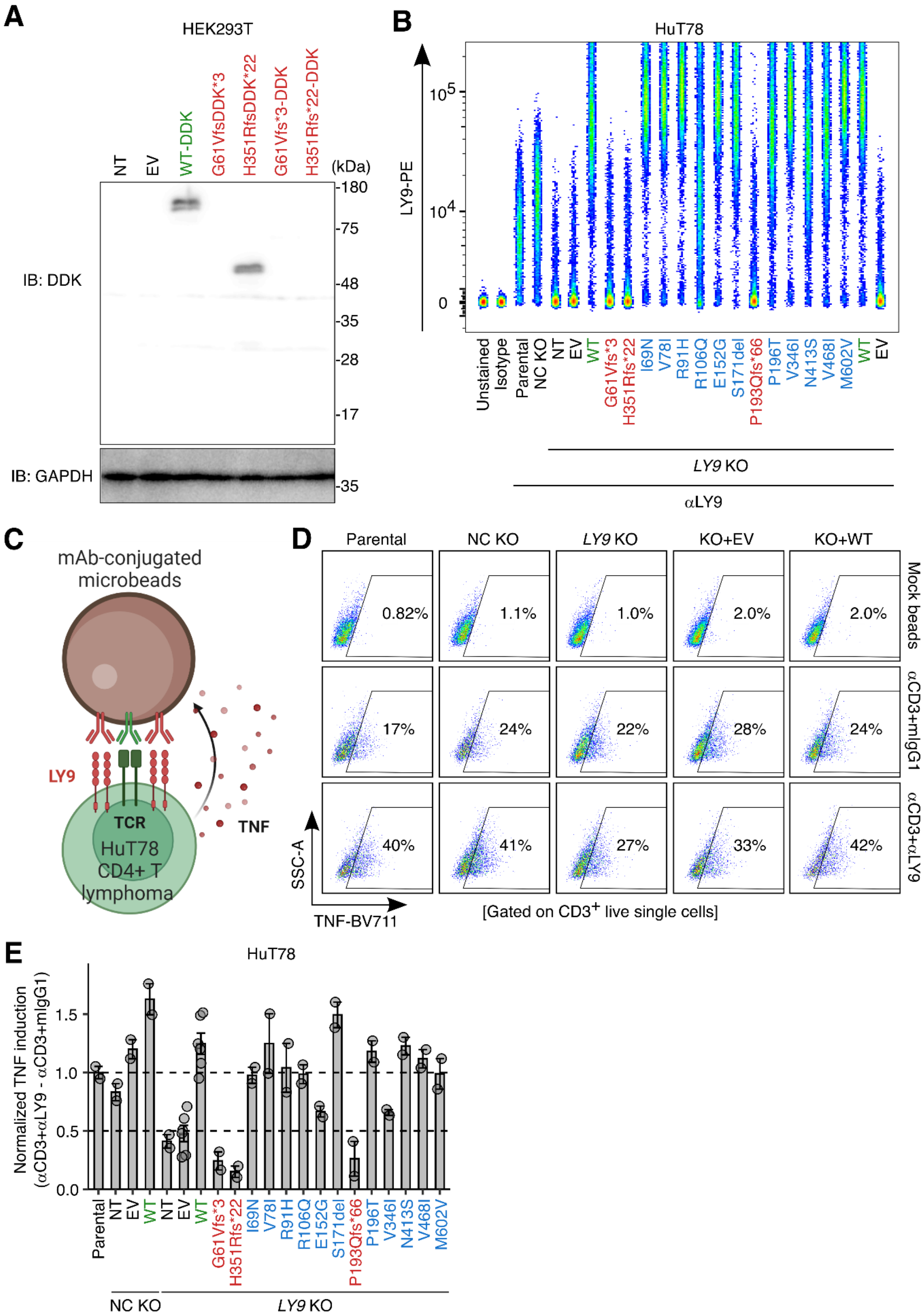
A
HEK293T
NT
EV
WT-DDK
G61VfsDDK*3
H351RfsDDK*22
G61Vfs*3-DDK
H351Rfs*22-DDK
(kDa)
-180
-75
-48
-35
-28
-17
IB: DDK
IB: GAPDH
-35
B
HuT78
LY9-PE
10^5
10^4
0
Unstained
Isotype
Parental
NC KO
NT
EV
WT
G61Vfs*3
H351Rfs*22
I69N
V78I
R91H
R106Q
E152G
S171del
P193Qfs*66
P196T
V346I
N413S
V468I
M602V
WT
EV
LY9 KO
αLY9
C
mAb-conjugated microbeads
LY9
TCR
HuT78 CD4+ T lymphoma
TNF
D
Parental
NC KO
LY9 KO
KO+EV
KO+WT
0.82%
1.1%
1.0%
2.0%
2.0%
Mock beads
17%
24%
22%
28%
24%
αCD3+mIgG1
40%
41%
27%
33%
42%
αCD3+αLY9
SSC-A
TNF-BV711
[Gated on CD3+ live single cells]
E
HuT78
Normalized TNF induction (αCD3+αLY9 - αCD3+mIgG1)
1.5
1.0
0.5
0.0
Parental
NT
EV
WT
NT
EV
WT
G61Vfs*3
H351Rfs*22
I69N
V78I
R91H
R106Q
E152G
S171del
P193Qfs*66
P196T
V346I
N413S
V468I
M602V
NC KO
LY9 KO

Figure S2. Analysis of *LY9* alleles in an overexpression system. (A) HEK293T cells were transfected with an empty vector (EV) or plasmids encoding the wild-type (WT) or truncated LY9 proteins with a C-terminal DDK tag or truncated LY9 proteins with a DDK tag inserted immediately upstream from the newly created stop codon in frameshift variants. Representative results from two independent experiments are shown. (B and C) HuT78 T-lymphoma cells with CRISPR/Cas9-mediated *LY9* knockout (KO) or nucleofection with a scrambled negative control (NC) sgRNA were lentivirally transduced with WT LY9 or variants. Transduced cells were selected with puromycin. (B) Surface LY9 expression, as determined by flow cytometry with a PE-conjugated anti-LY9 mAb. (C-E) LY9 crosslinking assay. (C) Schematic diagram. Cells were stimulated by incubation for 2 hours with bead-conjugated mAbs and secretion inhibitors, and the levels of TNF accumulating within the cells were determined by flow cytometry. (D) Representative plots. (E) Summary. The increase in the percentage of TNF-producing cells due to LY9 crosslinking was calculated and normalized by dividing by the mean value for parental HuT78 cells. Technical duplicates were performed for all conditions. *LY9* KO cells were transduced with EV and WT LY9 and tested three times. Representative results from two independent experiments are shown.

Figure S3

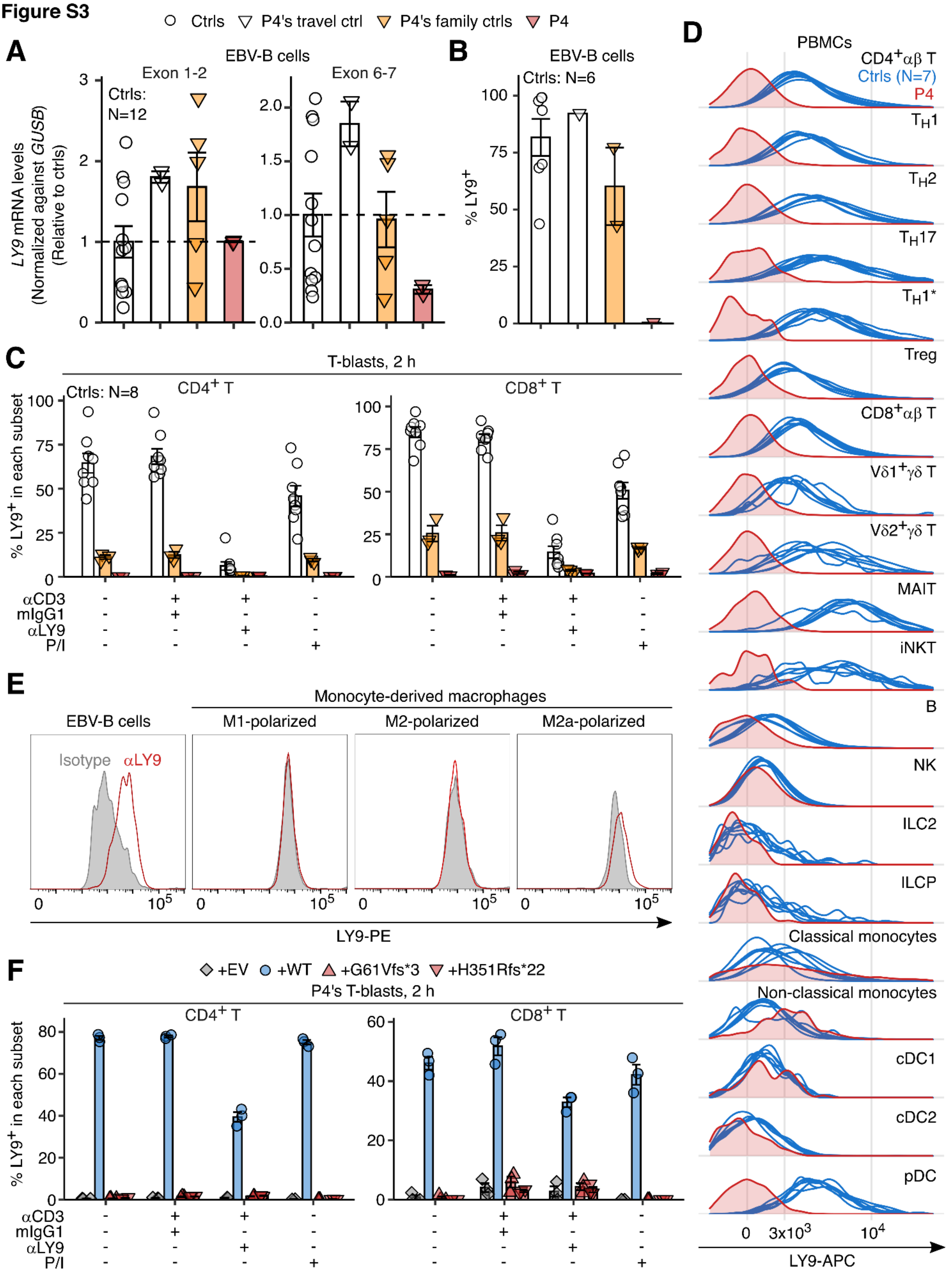
Ctrls
P4's travel ctrl
P4's family ctrls
P4
A
EBV-B cells
Exon 1-2
Exon 6-7
Ctrls: N=12
LY9 mRNA levels (Normalized against GUSB) (Relative to ctrls)
B
EBV-B cells
Ctrls: N=6
% LY9+
C
T-blasts, 2 h
Ctrls: N=8
CD4+ T
CD8+ T
% LY9+ in each subset
αCD3
mIgG1
αLY9
P/I
D
PBMCs
CD4+αβ T
Ctrls (N=7)
P4
TH1
TH2
TH17
TH1*
Treg
CD8+αβ T
Vδ1+γδ T
Vδ2+γδ T
MAIT
iNKT
B
NK
ILC2
ILCP
Classical monocytes
Non-classical monocytes
cDC1
cDC2
pDC
0
3x10^3
10^4
LY9-APC
E
EBV-B cells
Monocyte-derived macrophages
M1-polarized
M2-polarized
M2a-polarized
Isotype
αLY9
LY9-PE
F
+EV
+WT
+G61Vfs*3
+H351Rfs*22
P4's T-blasts, 2 h
CD4+ T
CD8+ T
% LY9+ in each subset
αCD3
mIgG1
αLY9
P/I

Figure S3. Analysis of endogenous LY9 expression. (A) *LY9* mRNA levels in EBV-B cells, as determined by RT-qPCR. Two different probes targeting *LY9* were used. *GUSB* was used as an endogenous control. Results from two experiments were compiled. (B-D) Surface LY9 expression on P4's (B) EBV-B cells, (C) T-blasts, (D) lymphoid and myeloid cell subsets of PBMCs. In B and C, representative results from two independent experiments are shown. In E, the experiment was performed once. (E) Surface LY9 expression on macrophages derived from peripheral blood monocytes from one healthy donor. EBV-B cells were used as a positive control. Representative results from two independent experiments. Red, anti-LY9 mAb. Gray, isotype control. (F) T-blasts from P4 transduced with EV, WT LY9, or two frameshift variants, with determination by flow cytometry. Representative results from two independent experiments are shown. P/I, phorbol 12-myristate 13-acetate (PMA) and ionomycin. Bars represent the mean and SEM.

Figure S4

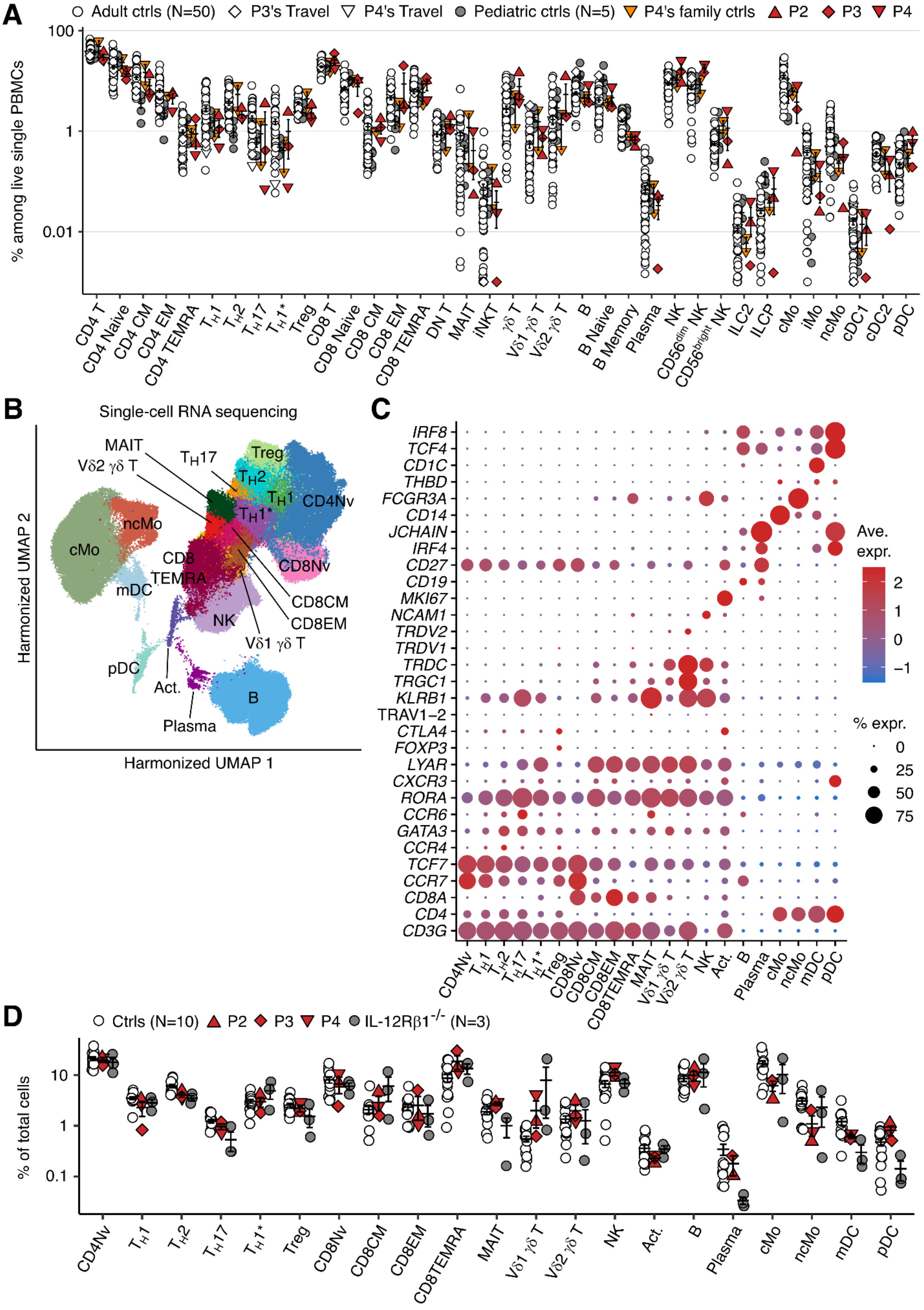

A
Adult ctrls (N=50)
P3's Travel
P4's Travel
Pediatric ctrls (N=5)
P4's family ctrls
P2
P3
P4
% among live single PBMCs
100
1
0.01
CD4 T
CD4 Naive
CD4 CM
CD4 EM
CD4 TEMRA
TH1
TH2
TH17
TH1*
Treg
CD8 T
CD8 Naive
CD8 CM
CD8 EM
CD8 TEMRA
DN T
MAIT
iNKT
γδ T
Vδ1 γδ T
Vδ2 γδ T
B
B Naive
B Memory
Plasma
NK
CD56dim NK
CD56bright NK
ILC2
ILCP
cMo
iMo
ncMo
cDC1
cDC2
pDC
B
Single-cell RNA sequencing
Harmonized UMAP 2
Harmonized UMAP 1
MAIT
Vδ2 γδ T
TH17
Treg
TH2
TH1
CD4Nv
TH1*
ncMo
cMo
CD8 TEMRA
CD8Nv
mDC
CD8CM
NK
CD8EM
Vδ1 γδ T
pDC
Act.
B
Plasma
C
IRF8
TCF4
CD1C
THBD
FCGR3A
CD14
JCHAIN
IRF4
CD27
CD19
MKI67
NCAM1
TRDV2
TRDV1
TRDC
TRGC1
KLRB1
TRAV1−2
CTLA4
FOXP3
LYAR
CXCR3
RORA
CCR6
GATA3
CCR4
TCF7
CCR7
CD8A
CD4
CD3G
CD4Nv
TH1
TH2
TH17
TH1*
Treg
CD8Nv
CD8CM
CD8EM
CD8TEMRA
MAIT
Vδ1 γδ T
Vδ2 γδ T
NK
Act.
B
Plasma
cMo
ncMo
mDC
pDC
Ave. expr.
2
1
0
−1
% expr.
0
25
50
75
D
Ctrls (N=10)
P2
P3
P4
IL-12Rβ1-/- (N=3)
% of total cells
10
1
0.1
CD4Nv
TH1
TH2
TH17
TH1*
Treg
CD8Nv
CD8CM
CD8EM
CD8TEMRA
MAIT
Vδ1 γδ T
Vδ2 γδ T
NK
Act.
B
Plasma
cMo
ncMo
mDC
pDC

Figure S4. Presence of all lymphoid and myeloid leukocyte subsets in LY9-deficient individuals. (A) Immunophenotyping. PBMCs from P2, P3, and P4 (aged 29, 40, and 15 years, respectively) were analyzed by flow cytometry. Results from four experiments were compiled. (B-D) Single-cell transcriptomic analysis. Single-cell RNA sequencing (scRNASeq) was performed on PBMCs from P2, P3, and P4 (aged 29, 40, and 16 years, respectively), three IL-12Rβ1-deficient patients, and 10 healthy controls. (B) Clustering analysis. Graph-based clustering was conducted after the removal of batch effects with Harmony (*99*). Data from seven batches of experiments were integrated. Clusters were identified with SingleR (*100*) guided by the Monaco datasets (*68*), followed by manual inspection. (C) Representative marker gene expression. (D) Proportions of annotated cell subsets. In A and D, bars represent the mean and SEM.

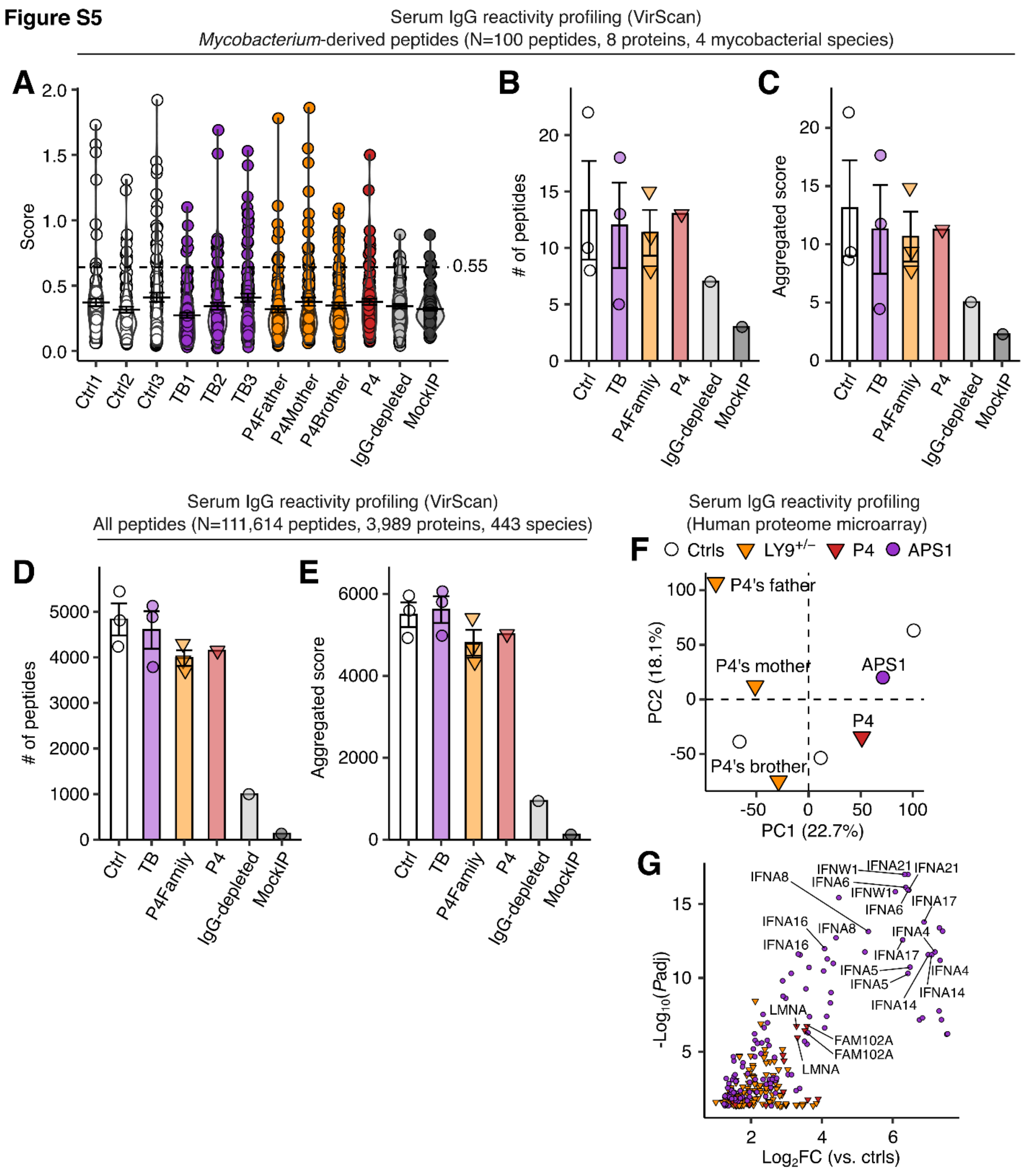

Figure S5. Normal serum antibody repertoire in LY9 deficiency. (A-E) VirScan. Serum IgG reactivity was profiled against a library of phages displaying diverse 45aa linear peptides derived from microbial pathogens. Serum samples from P4 (aged 15 years), P4's relatives, three TB patients, and three healthy donors were analyzed. (A-C) *Mycobacterium*-derived peptides. (A) Reactivity score distribution. The horizontal dotted line (0.55) indicates the upper limit of the 95% confidence interval

for the two negative controls (i.e., IgG-depleted and mock immunoprecipitation controls) as a cutoff. (B) The number of peptides above the cutoff (0.55). (C) Aggregate scores of peptides above the cutoff (0.55). (D and E) All microbial peptides. (D) The number of peptides above the cutoff. (E) Aggregate scores of peptides above the cutoff. (F and G) Serum IgG autoreactivity profiling with a human proteome microarray. (F) PCA. (G) Differential reactivity analysis between healthy controls and a comparator. P4, P4's relatives (brother, mother, and father), and one APS-1 patient were each compared with healthy controls. Only peptides more strongly recognized (i.e., $log_2FC > 1$ and FDR-adjusted *P* value < 0.05) in the comparator than in healthy controls were retained. We then retained only protein antigens with two or more significantly enriched peptides. The log_2FC and FDR-adjusted *P* values for the retained peptides for each individual are shown. Protein antigens of interest are labeled. In A-E, bars represent the mean and SEM.

Figure S6

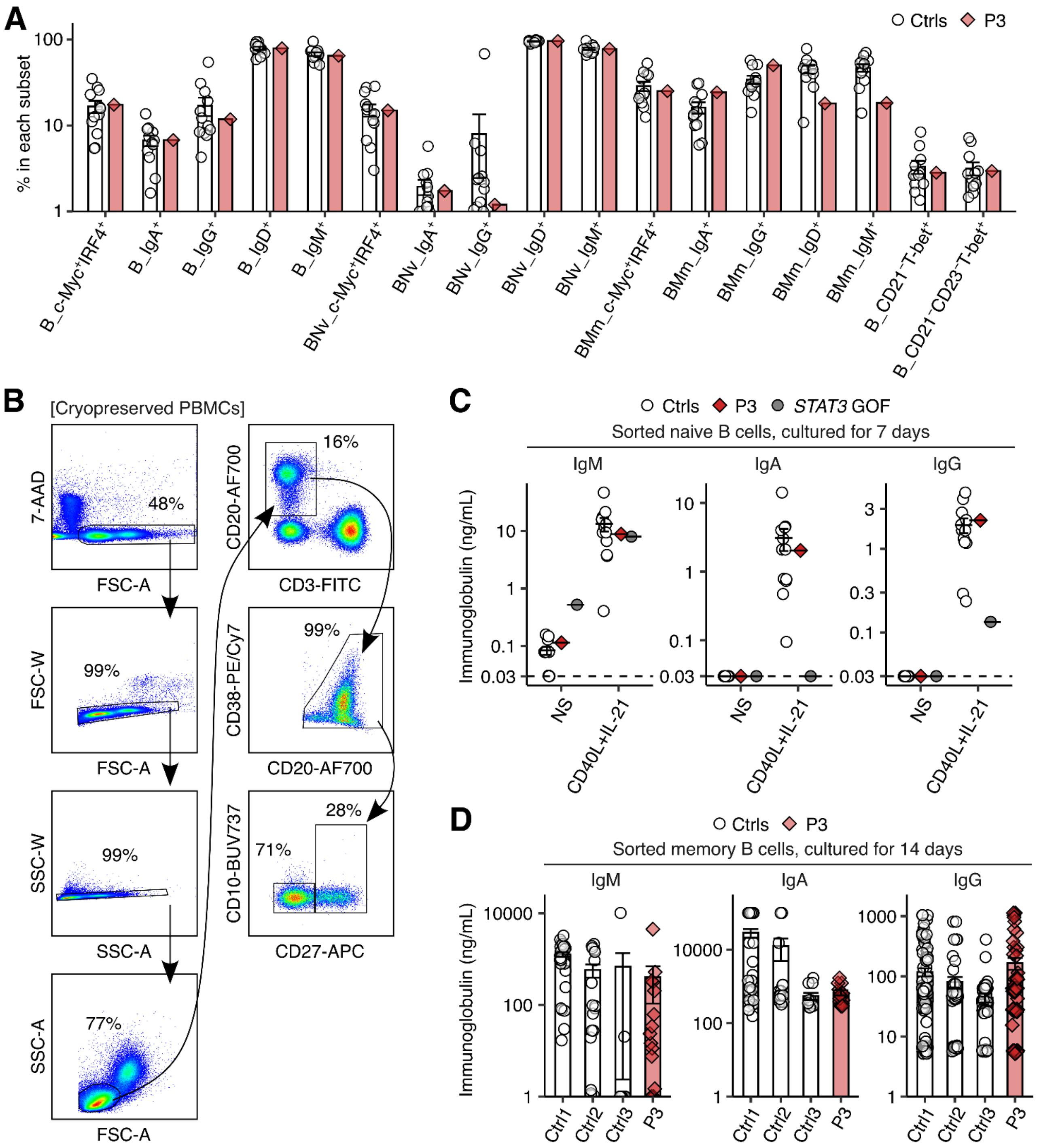

Figure S6. Normal B-cell phenotypes in LY9 deficiency. PBMCs from P3 (aged 40 years) and healthy controls were analyzed. (A) B-cell phenotyping by flow cytometry. (B) Sorting strategy for naïve and memory B cells. (C) Naïve B-cell stimulation assay. Naive B cells sorted from the PBMCs of P3, one patient heterozygous for a *STAT3* gain-of-function (GOF) mutation, and healthy controls were cultured for seven days. Immunoglobulin levels were determined in a LEGENDplex assay. (D)

Memory B-cell expansion assay. Memory B cells sorted from the PBMCs of P3 and three healthy donors were cultured in optimized conditions for B-cell expansion for 14 days (Method). Immunoglobulin levels were determined in a LEGENDplex assay. In A, C, and D, bars represent the mean and SEM. A subset of data for the healthy controls and the *STAT3* gain-of-function (GOF) patient in A, C, and D have been published elsewhere (*85*).

Figure S7

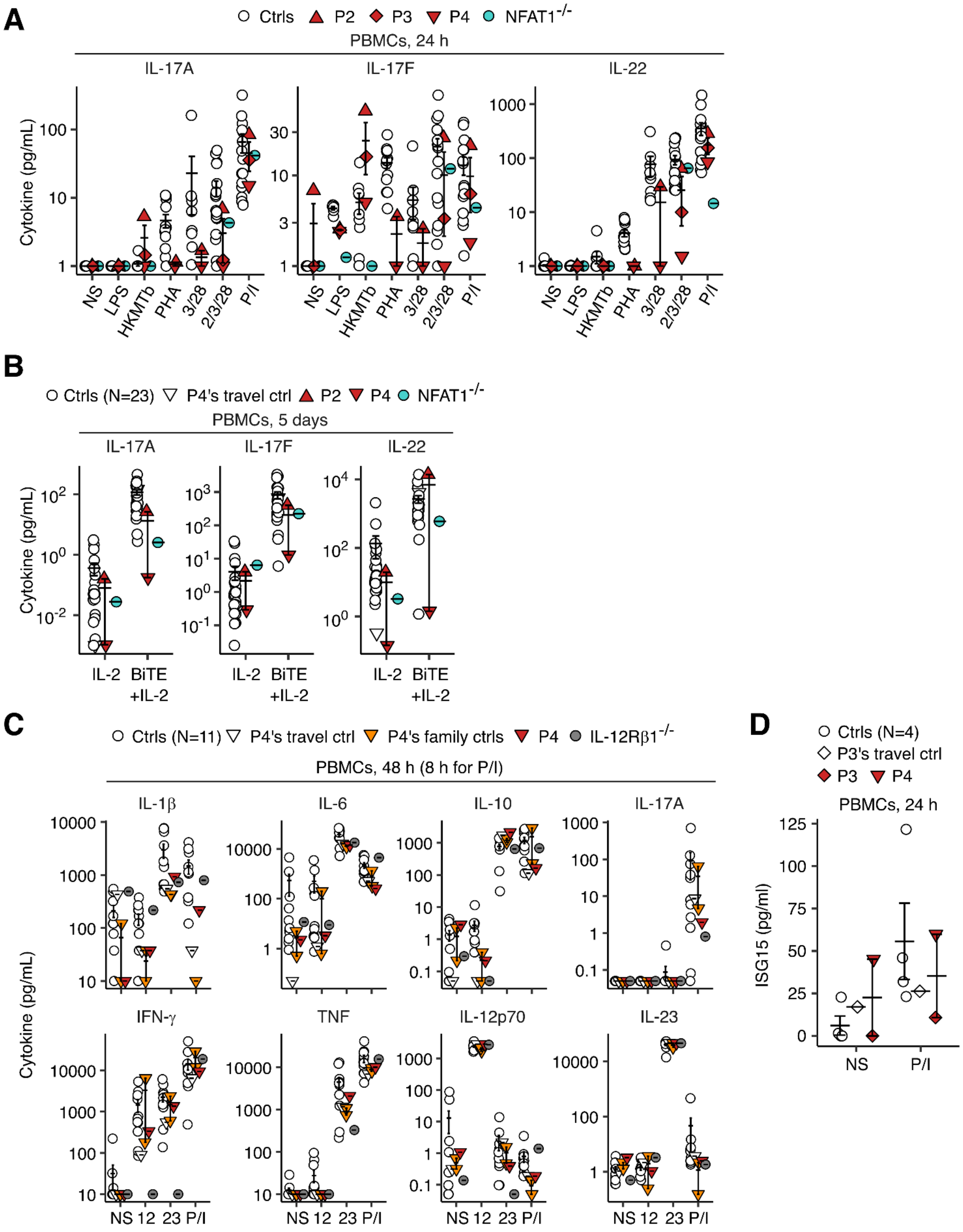

Figure S7. Analysis of the cellular responses of LY9-deficient leukocytes. (A) PBMCs from P2, P3, P4 (aged 29, 40, and 17 years, respectively), one NFAT1-deficient patient (*50*), and healthy

controls were stimulated with the indicated stimuli for 24 hours. The cytokines secreted into the medium were determined in a LEGENDplex assay. Results from three experiments are compiled, with all technical replicates averaged. (B) PBMCs from P2, P4 (aged 29 and 17, respectively), one NFAT1-deficient patient, and healthy controls were stimulated with blinatumomab (a bispecific antibody targeting CD3 and CD19 to induce immune synapses between autologous T and B lymphocytes) for 5 days. The cytokines secreted into the medium were determined in a LEGENDplex assay. Results from eight experiments are compiled, with all technical replicates averaged. (C) PBMCs from healthy controls, P4's travel control, P4's family controls, P4 (aged 15 years, in complete remission from TB), and one IL-12Rβ1-deficient patient were stimulated with IL-12 or IL-23 for 48 hours or P/I for 8 hours. Secreted cytokine levels were determined in a LEGENDplex assay. The experiment was performed once. (D) PBMCs from healthy controls, P3's travel control, P3, and P4 (aged 40 and 17 years, respectively; both patients were in complete remission from TB) were stimulated with P/I for 24 hours. Secreted levels of ISG15 were determined by ELISA. Results from two experiments were compiled. Bars represent the mean and SEM. LPS, lipopolysaccharides; HKMTb, heat-killed *M.tb* lysate; PHA, phytohemagglutinin; BiTE, blinatumomab; P/I, PMA and ionomycin.

Figure S8

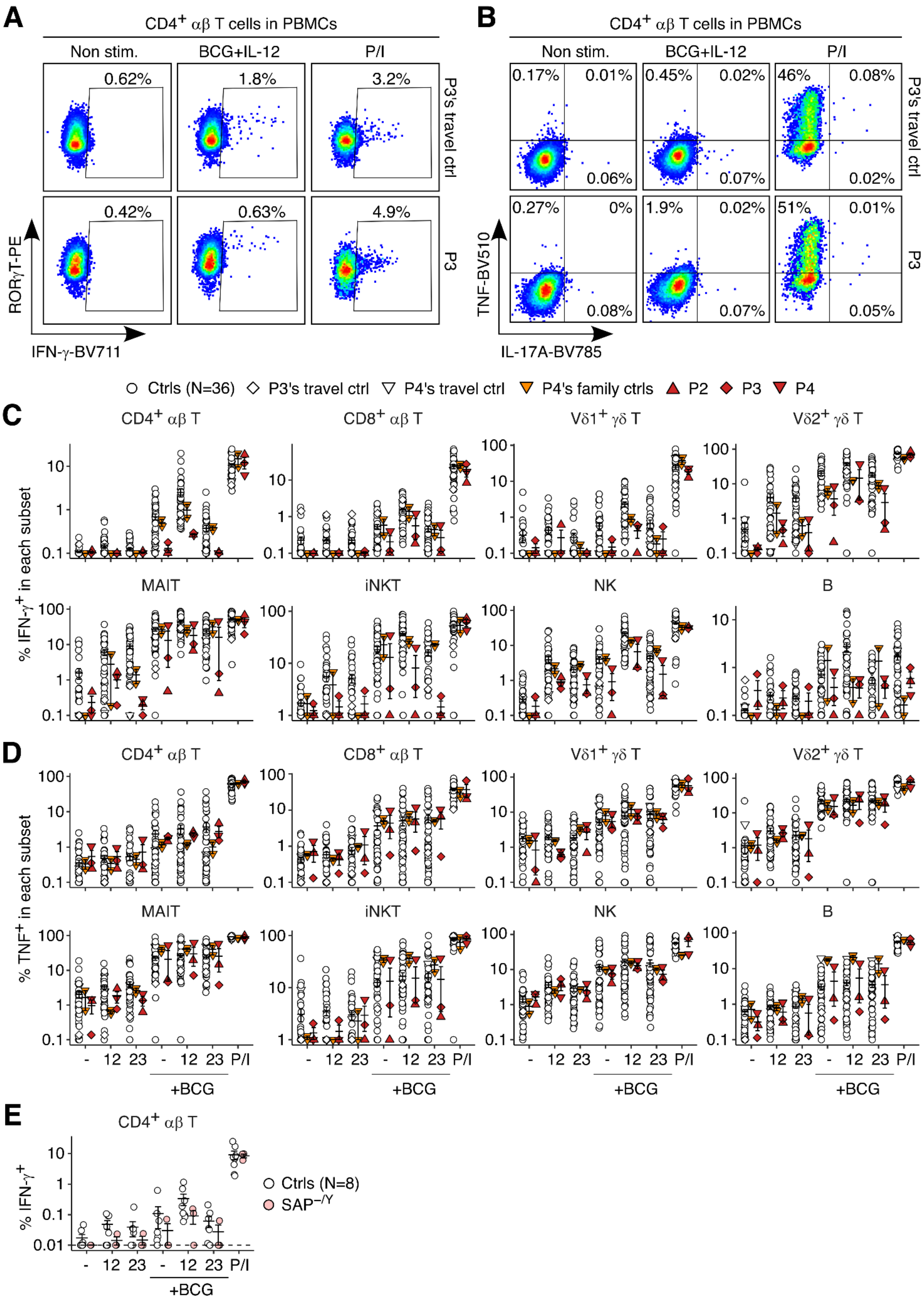

A
CD4+ αβ T cells in PBMCs
Non stim.
BCG+IL-12
P/I
0.62%
1.8%
3.2%
0.42%
0.63%
4.9%
P3's travel ctrl
P3
RORγT-PE
IFN-γ-BV711
B
CD4+ αβ T cells in PBMCs
Non stim.
BCG+IL-12
P/I
0.17% 0.01% 0.06%
0.45% 0.02% 0.07%
46% 0.08% 0.02%
0.27% 0% 0.08%
1.9% 0.02% 0.07%
51% 0.01% 0.05%
TNF-BV510
IL-17A-BV785
Ctrls (N=36)
P3's travel ctrl
P4's travel ctrl
P4's family ctrls
P2
P3
P4
C
CD4+ αβ T
CD8+ αβ T
Vδ1+ γδ T
Vδ2+ γδ T
MAIT
iNKT
NK
B
% IFN-γ+ in each subset
D
CD4+ αβ T
CD8+ αβ T
Vδ1+ γδ T
Vδ2+ γδ T
MAIT
iNKT
NK
B
% TNF+ in each subset
- 12 23 - 12 23 P/I
+BCG
E
CD4+ αβ T
% IFN-γ+
Ctrls (N=8)
SAP−/Y
- 12 23 - 12 23 P/I
+BCG

Figure S8. Analysis of cellular responses to mycobacterial stimulation *in vitro*. PBMCs from P2, P3, P4 (aged 29, 40, and 15 years, respectively; P2 had no unusually severe infections; P3 and P4 were in complete remission from TB and off all treatment), and healthy donors were stimulated with live BCG mycobacteria with or without IL-12 or IL-23 for 48 hours. Secretion inhibitors were added for the last 8 hours. Intracellular cytokine levels were quantified by flow cytometry. (A and B) Representative plots for (A) IFN-γ and (B) IL-17A/TNF production in $CD4^+$ αβ T lymphocytes. (C and D) Summary of four experiments, with P2, P3, and P4 tested on different occasions. (C) IFN-γ production by lymphocyte subsets. (D) TNF production by lymphocyte subsets. (E) IFN-γ production by $CD4^+$ αβ T lymphocytes from three SAP-deficient patients. In C-E, bars represent the mean and SEM. P/I, PMA and ionomycin.

Figure S9

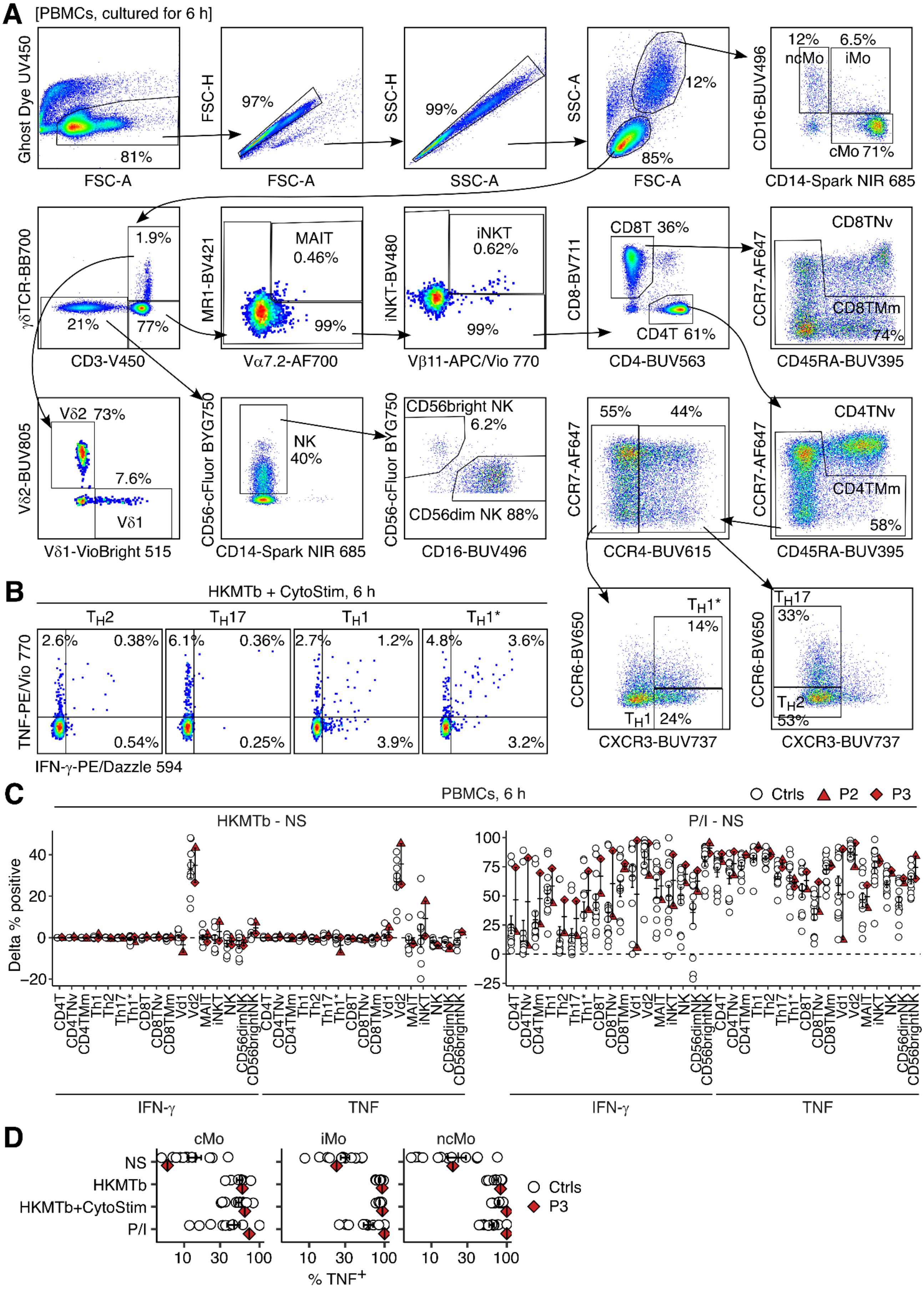

A
[PBMCs, cultured for 6 h]
Ghost Dye UV450
FSC-A
81%
FSC-H
97%
SSC-H
SSC-A
99%
12%
85%
CD16-BUV496
CD14-Spark NIR 685
12% ncMo
6.5% iMo
cMo 71%
γδTCR-BB700
CD3-V450
1.9%
21%
77%
MR1-BV421
Vα7.2-AF700
MAIT 0.46%
99%
iNKT-BV480
Vβ11-APC/Vio 770
iNKT 0.62%
99%
CD8-BV711
CD4-BUV563
CD8T 36%
CD4T 61%
CCR7-AF647
CD45RA-BUV395
CD8TNv
CD8TMm
74%
Vδ2-BUV805
Vδ1-VioBright 515
Vδ2 73%
7.6%
Vδ1
CD56-cFluor BYG750
NK 40%
CD16-BUV496
CD56bright NK 6.2%
CD56dim NK 88%
CCR4-BUV615
55%
44%
CD4TNv
CD4TMm
58%
B
HKMTb + CytoStim, 6 h
TH2
TH17
TH1
TH1*
TNF-PE/Vio 770
IFN-γ-PE/Dazzle 594
2.6%
0.38%
0.54%
6.1%
0.36%
0.25%
2.7%
1.2%
3.9%
4.8%
3.6%
3.2%
CCR6-BV650
CXCR3-BUV737
TH1* 14%
TH1 24%
TH17 33%
TH2 53%
C
PBMCs, 6 h
Ctrls
P2
P3
HKMTb - NS
P/I - NS
Delta % positive
CD4T
CD4TNv
CD4TMm
Th1
Th2
Th17
Th1*
CD8T
CD8TNv
CD8TMm
Vd1
Vd2
MAIT
iNKT
NK
CD56dimNK
CD56brightNK
IFN-γ
TNF
D
cMo
iMo
ncMo
NS
HKMTb
HKMTb+CytoStim
P/I
% TNF+
Ctrls
P3

Figure S9. Analysis of IFN-γ and TNF production by LY9-deficient lymphoid and myeloid leukocyte subsets. PBMCs from P2 and P3 (aged 29 and 40 years, respectively) and healthy donors were either left non-stimulated or were stimulated with the indicated reagents for 6 hours with secretion inhibitors. Cytokine production was assessed by intracellular staining and flow cytometry. (A) Gating strategy. (B) IFN-γ and TNF production by the four T-helper subsets in a representative healthy donor. (C) IFN-γ and TNF production by lymphocyte subsets. The percentage of IFN-γ- or TNF-producing cells within each subset was quantified. The background without stimulation was subtracted. (D) TNF production by the three monocyte subsets. Bars represent the mean and SEM. HKMTb, heat-killed *M.tb* lysate; P/I, PMA and ionomycin.

Figure S10

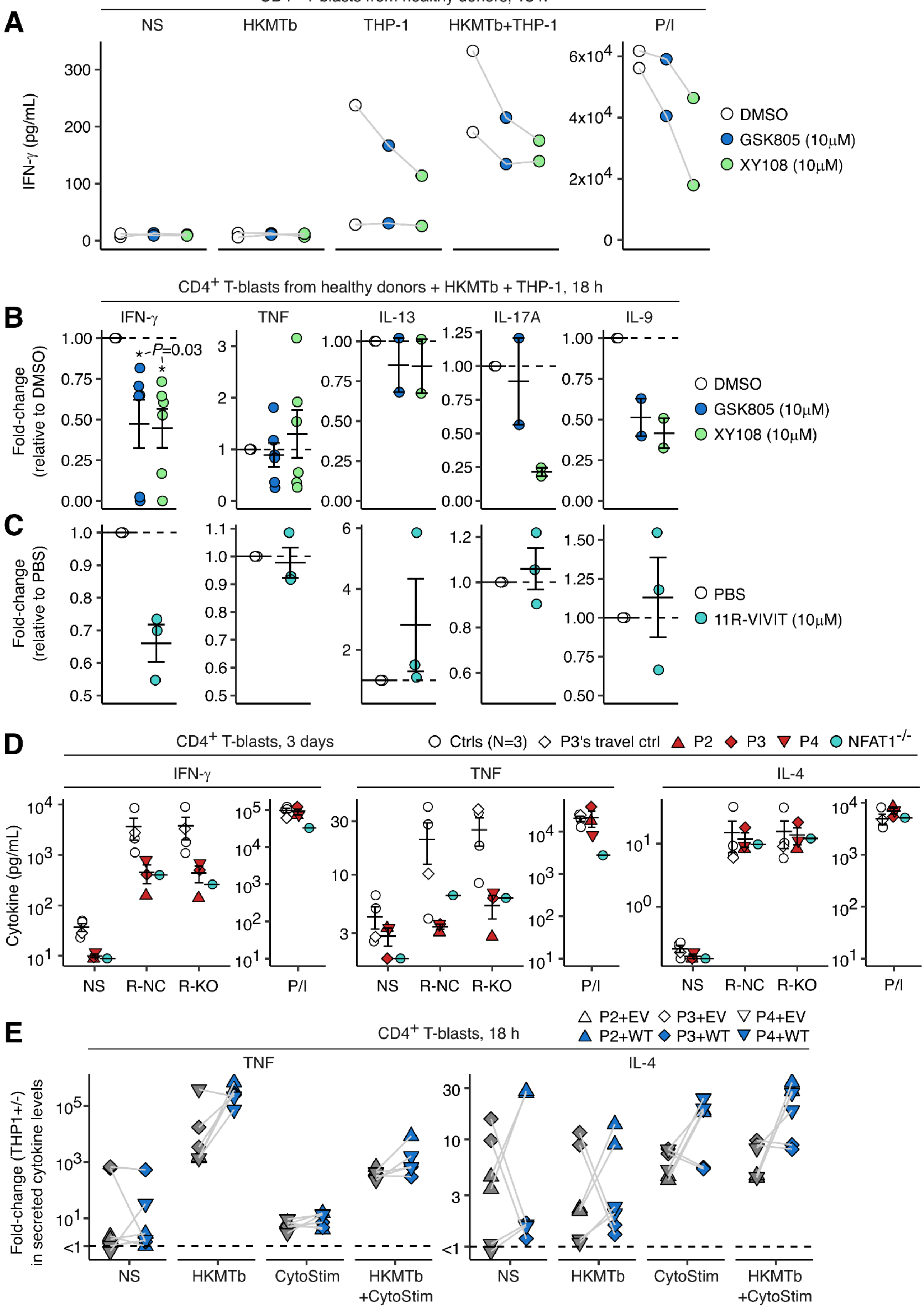

A
CD4+ T-blasts from healthy donors, 18 h
NS
HKMTb
THP-1
HKMTb+THP-1
P/I
IFN-γ (pg/mL)
DMSO
GSK805 (10μM)
XY108 (10μM)
B
CD4+ T-blasts from healthy donors + HKMTb + THP-1, 18 h
IFN-γ
TNF
IL-13
IL-17A
IL-9
P=0.03
Fold-change (relative to DMSO)
C
Fold-change (relative to PBS)
PBS
11R-VIVIT (10μM)
D
CD4+ T-blasts, 3 days
Ctrls (N=3)
P3's travel ctrl
P2
P3
P4
NFAT1-/-
IFN-γ
TNF
IL-4
Cytokine (pg/mL)
NS
R-NC
R-KO
P/I
E
CD4+ T-blasts, 18 h
P2+EV
P3+EV
P4+EV
P2+WT
P3+WT
P4+WT
TNF
IL-4
Fold-change (THP1+/-) in secreted cytokine levels
NS
HKMTb
CytoStim
HKMTb +CytoStim

Figure S10. Mechanistic analysis of cytokine production by CD4$^+$ T lymphocytes. (A-C) Pharmacological inhibition. CD4$^+$ T-blasts from healthy donors were cocultured with THP-1 monocytic leukemia cells and heat-killed *M.tb* (HKMTb) for 18 hours with two RORγT inhibitors (GSK805 and XY108), a cell-permeable NFAT inhibitor (11R-VIVIT peptide), or corresponding vehicle controls. Secreted cytokine levels were determined in a LEGENDplex assay. (A) Representative results for IFN-γ secretion by cells from two healthy controls with RORγT inhibition. (B and C) Fold-change decrease in secreted cytokine levels relative to vehicle controls for (B) RORγT inhibition (*N*=6 controls for IFN-γ and TNF; *N*=2 for other cytokines) and (C) NFAT inhibition (*N*=3 controls). For IFN-γ in the RORγT inhibition assay, the same set of data is also presented in Fig. 4F. (D) Raji B-lymphoma cell coculture assay. CD4$^+$ T-blasts from P2, P3, P4, one NFAT1-deficient patient, and four healthy controls (one of whom was P3's travel control) were cocultured with Raji B-lymphoma cells knocked out for *LY9* (R-KO) or treated with a scramble negative control sgRNA (R-NC) for 3 days. Secreted cytokine levels were determined in a LEGENDplex assay. (E) CD4$^+$ T-blasts from P2, P3, and P4 lentivirally transduced with empty vector (EV) or WT LY9 were cocultured with THP-1 cells and the indicated reagents. Secreted cytokine levels were determined in LEGENDplex assays. The fold-change in secreted cytokine levels was determined by dividing cytokine levels in the presence of THP-1 cells by those in the absence of THP-1 cells. In A, B, and D, bars represent the mean and SEM. HKMTb, heat-killed *M.tb* lysate; P/I, PMA and ionomycin.

Figure S11

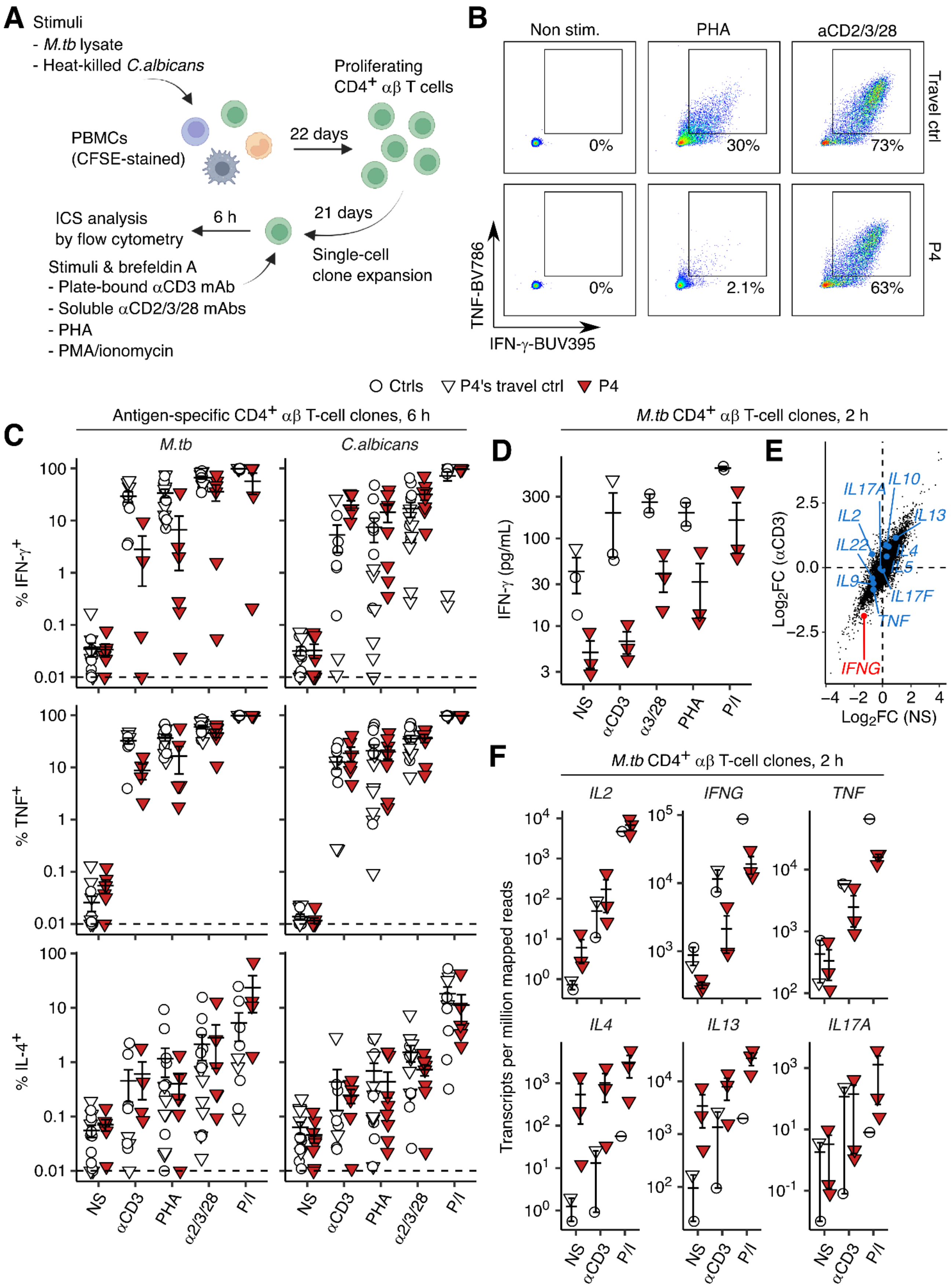
A
Stimuli
- M.tb lysate
- Heat-killed C.albicans
PBMCs (CFSE-stained)
22 days
Proliferating CD4+ αβ T cells
21 days
Single-cell clone expansion
6 h
ICS analysis by flow cytometry
Stimuli & brefeldin A
- Plate-bound αCD3 mAb
- Soluble αCD2/3/28 mAbs
- PHA
- PMA/ionomycin
B
Non stim.
PHA
aCD2/3/28
Travel ctrl
P4
0%
30%
73%
0%
2.1%
63%
TNF-BV786
IFN-γ-BUV395
Ctrls
P4's travel ctrl
P4
C
Antigen-specific CD4+ αβ T-cell clones, 6 h
M.tb
C.albicans
% IFN-γ+
% TNF+
% IL-4+
NS
αCD3
PHA
α2/3/28
P/I
D
M.tb CD4+ αβ T-cell clones, 2 h
IFN-γ (pg/mL)
NS
αCD3
α3/28
PHA
P/I
E
Log2FC (αCD3)
Log2FC (NS)
IL17A
IL10
IL2
IL13
IL22
IL4
IL5
IL9
IL17F
TNF
IFNG
F
M.tb CD4+ αβ T-cell clones, 2 h
IL2
IFNG
TNF
IL4
IL13
IL17A
Transcripts per million mapped reads
NS
αCD3
P/I

Figure S11. Analysis of cytokine production by LY9-deficient antigen-specific CD4$^+$ αβ T-cell clones. (A) Experimental design. CFSE-stained PBMCs from P4 (aged 17 years), his travel control, and BCG-vaccinated controls were stimulated with antigens derived from *M.tb* or *C. albicans.* Reactive CD4$^+$ αβ T-cell clones were isolated, expanded, and restimulated with the indicated reagents. Cytokine production and intracellular protein levels were quantified by flow cytometry. Results from two experiments were compiled. (B) Representative plots. (C) Cytokine production. (D and E) Randomly selected *M.tb*-specific RORγT^{hi} CD4$^+$ αβ T-cell clones (*N*=3 from P4 and *N*=3 from healthy controls, one of whom was an ethnicity-matched travel control) were further expanded *in vitro* for 14 days and restimulated for 2 hours with various stimuli. (D) Secreted IFN-γ levels were determined in a ProQuantum immunoassay. Results from technical duplicates were averaged. (E and F) RNASeq analysis. Extracted RNA from technical duplicates was pooled before library preparation. (E) Differential expression (DE) between clones from controls and P4, as determined with DESeq2 (*90*) with effect-size shrinkage (*91*) in either non-stimulated or anti-CD3-stimulated conditions. Genes encoding cytokines of interest are colored. (F) Transcripts per million mapped reads (TPMs) for representative genes. In C, D, and F, bars represent the mean and SEM. PHA, phytohemagglutinin. P/I, PMA and ionomycin.

Figure S12

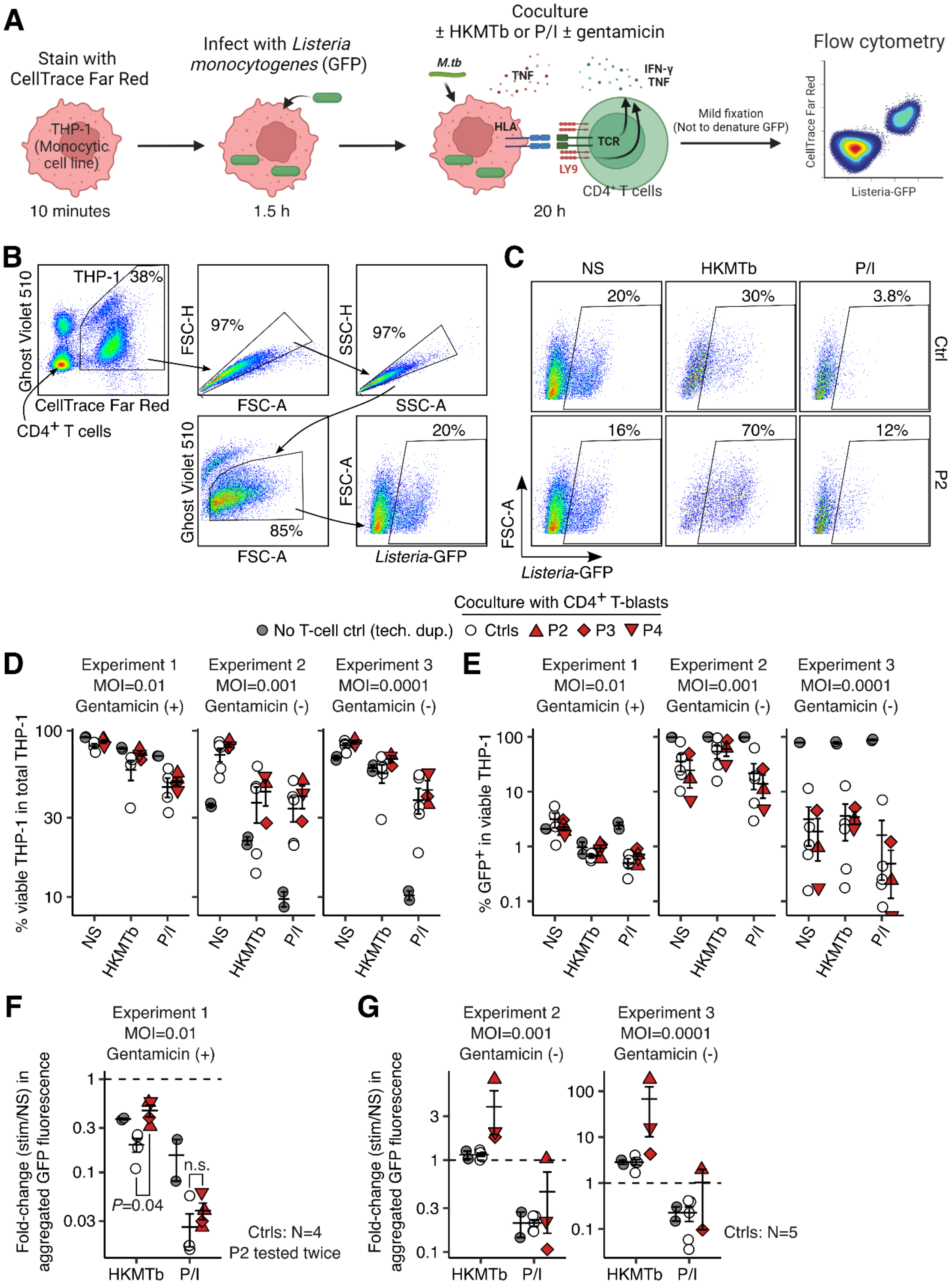
A
Stain with CellTrace Far Red
THP-1 (Monocytic cell line)
10 minutes
Infect with Listeria monocytogenes (GFP)
1.5 h
Coculture ± HKMTb or P/I ± gentamicin
M.tb
TNF
IFN-γ TNF
HLA
TCR
LY9
CD4+ T cells
20 h
Mild fixation (Not to denature GFP)
Flow cytometry
CellTrace Far Red
Listeria-GFP
B
THP-1 38%
Ghost Violet 510
CellTrace Far Red
CD4+ T cells
FSC-H
97%
FSC-A
SSC-H
97%
SSC-A
Ghost Violet 510
85%
FSC-A
FSC-A
20%
Listeria-GFP
C
NS
HKMTb
P/I
20%
30%
3.8%
Ctrl
16%
70%
12%
P2
FSC-A
Listeria-GFP
Coculture with CD4+ T-blasts
No T-cell ctrl (tech. dup.)
Ctrls
P2
P3
P4
D
Experiment 1 MOI=0.01 Gentamicin (+)
Experiment 2 MOI=0.001 Gentamicin (-)
Experiment 3 MOI=0.0001 Gentamicin (-)
% viable THP-1 in total THP-1
100
30
10
NS
HKMTb
P/I
E
Experiment 1 MOI=0.01 Gentamicin (+)
Experiment 2 MOI=0.001 Gentamicin (-)
Experiment 3 MOI=0.0001 Gentamicin (-)
% GFP+ in viable THP-1
100
10
1
0.1
NS
HKMTb
P/I
F
Experiment 1 MOI=0.01 Gentamicin (+)
Fold-change (stim/NS) in aggregated GFP fluorescence
1
0.3
0.1
0.03
P=0.04
n.s.
HKMTb
P/I
Ctrls: N=4
P2 tested twice
G
Experiment 2 MOI=0.001 Gentamicin (-)
Experiment 3 MOI=0.0001 Gentamicin (-)
Fold-change (stim/NS) in aggregated GFP fluorescence
3
1
0.3
0.1
100
10
1
0.1
HKMTb
P/I
Ctrls: N=5

Figure S12. Impairment of the ability of LY9-deficient CD4$^+$ T lymphocytes to restrict the growth of *Listeria monocytogenes* in THP-1 phagocytes. (A) Experimental design. *L. monocytogenes* expressing GFP was used as a probe of the capacity of THP-1 cells to restrict the growth of intramacrophagic microbes. (B) Gating strategy. (C) Representative results. (D-G) Three experiments were performed. In Experiments 1, 2, and 3, the initial infection of *L. monocytogenes* was performed at an MOI of 0.01, 0.001, and 0.0001, respectively. In Experiment 1, gentamicin was added during the coculture of *L. monocytogenes*-infected THP-1 and CD4$^+$ T-blasts to prevent the lateral spread of bacteria. (D) Viability of THP-1 cells. (E) Proportions of GFP$^+$ THP-1 cells. (F) Results from Experiment 1. Aggregate GFP expression levels in GFP$^+$ THP-1 cells were calculated as a surrogate metric for the total bacterial burden in the coculture. For assessment of the effect of external stimuli (i.e., HKMTb or P/I), the GFP levels in the coculture with external stimuli were divided by the GFP levels in the coculture without external stimuli. P2's CD4$^+$ T-blasts induced on different occasions were used as replicates. (G) Results from Experiments 2 and 3, as in F. In D-G, bars represent the mean and SEM. HKMTb, heat-killed *M.tb*. P/I, PMA and ionomycin.

Figure S13

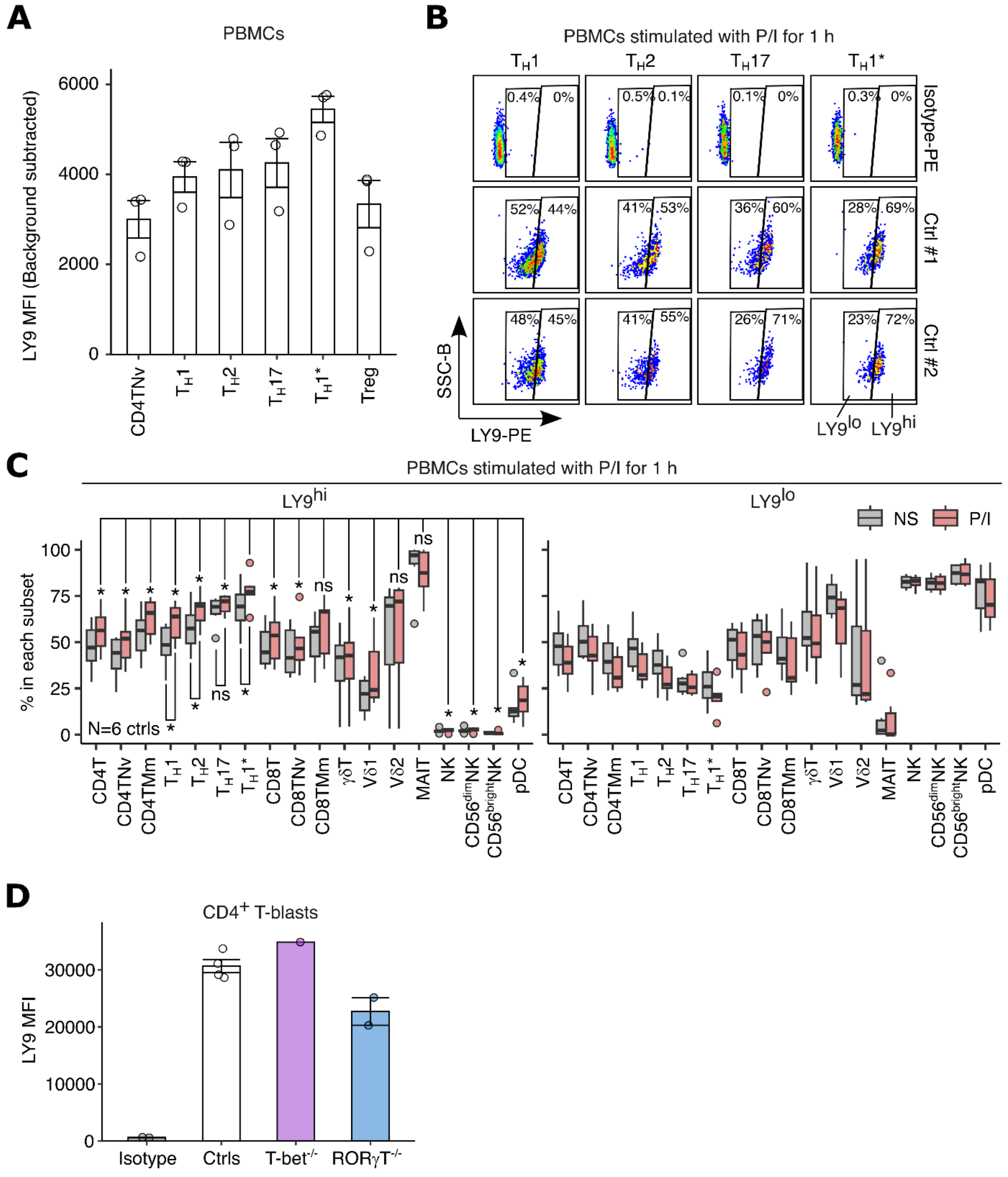

Figure S13. Quantitative analysis of LY9 expression in leukocyte subsets. (A) LY9 expression in leukocyte subsets in non-stimulated PBMCs. The same flow cytometry dataset is presented in Fig. 2F. Here, the LY9 MFI values in each subset are summarized. The values for P4's cells were considered to constitute the background. (B and C) LY9 expression in PBMCs stimulated with P/I for 1 hour. (B) Representative results for two healthy donors. (C) Summary plots. n.s., not significant. *, $P < 0.05$ by

unpaired Wilcoxon's rank sum tests. (D) LY9 expression in $CD4^+$ T-blasts from four healthy controls, one T-bet-deficient patient, and two RORγT-deficient patients.

Figure S14

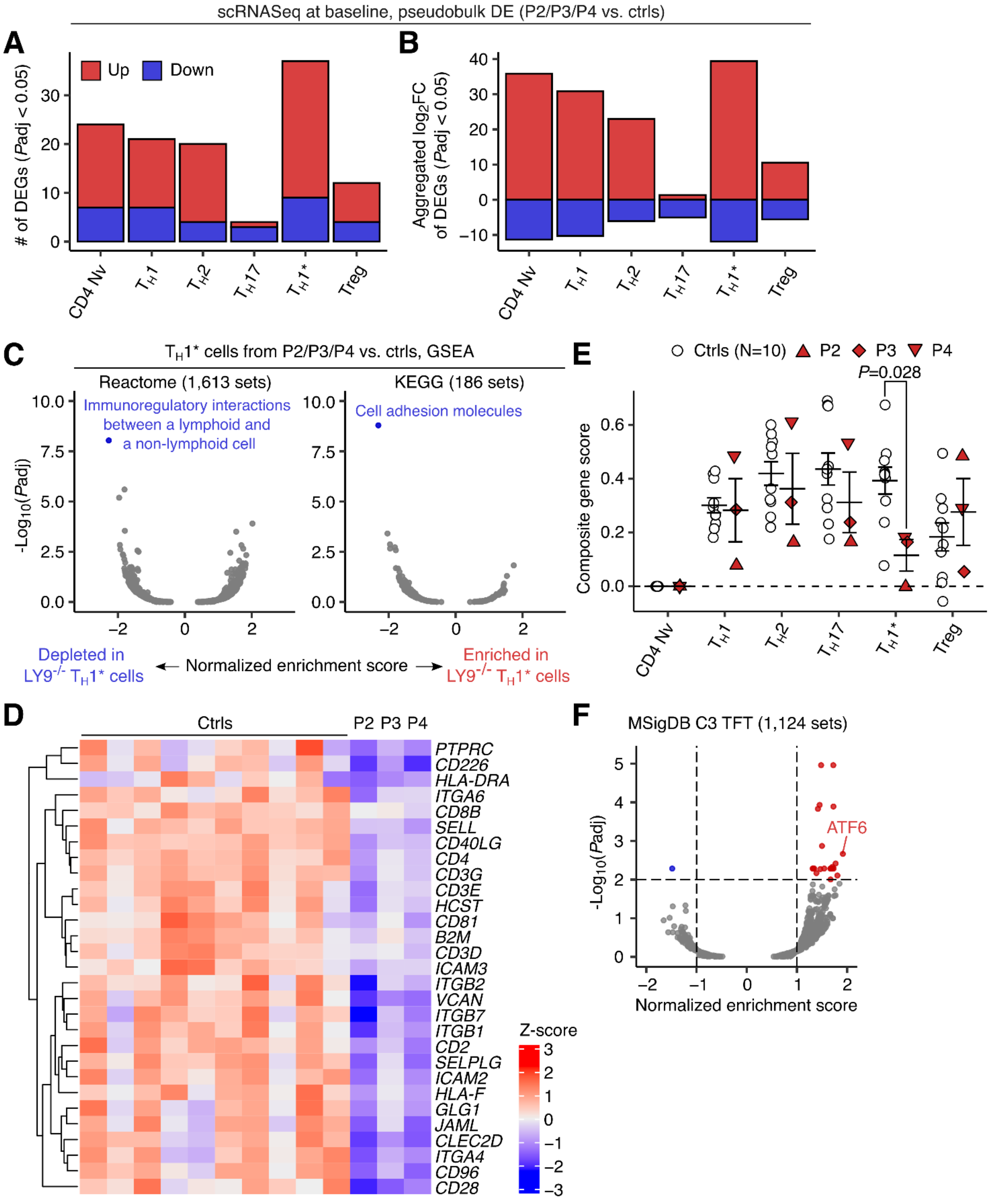

Figure S14. Transcriptomic analysis of LY9-deficient T$_{H}$**1* cells.** Single-cell RNA sequencing (scRNASeq) was performed on PBMCs from P2, P3, and P4 (aged 29, 40, and 16 years, respectively) and 10 healthy controls. Clustering analysis was performed as described in Fig. S4B-D. (A and B)

Pseudobulk differential expression (DE) analysis was was performed, comparing LY9-deficient and control CD4$^+$ T-cell subsets. (A) Numbers of DE genes (DEGs) upregulated or downregulated in LY9-deficient cells. (B) Aggregate log2FC values for up- and downregulated DEGs. (C) Geneset enrichment analysis (GSEA) was performed by projecting the fold-change-based gene rank onto the Reactome or KEGG pathway genesets. The genesets most strongly depleted in LY9-deficient TH1* cells are annotated. (D) Leading-edge genes for at least one of the two most depleted genesets shown in C. Z-transformed VST-transformed normalized pseudobulk read counts, shown as a heatmap. (E) Aggregate values of VST-transformed normalized pseudobulk read counts for the 29 leading-edge genes shown in D. The values for CD4 naïve T cells were used for normalization. Bars represent the mean and SEM. (F) GSEA for MsigDB C3 transcription factor motif genesets, as in C.

Figure S15

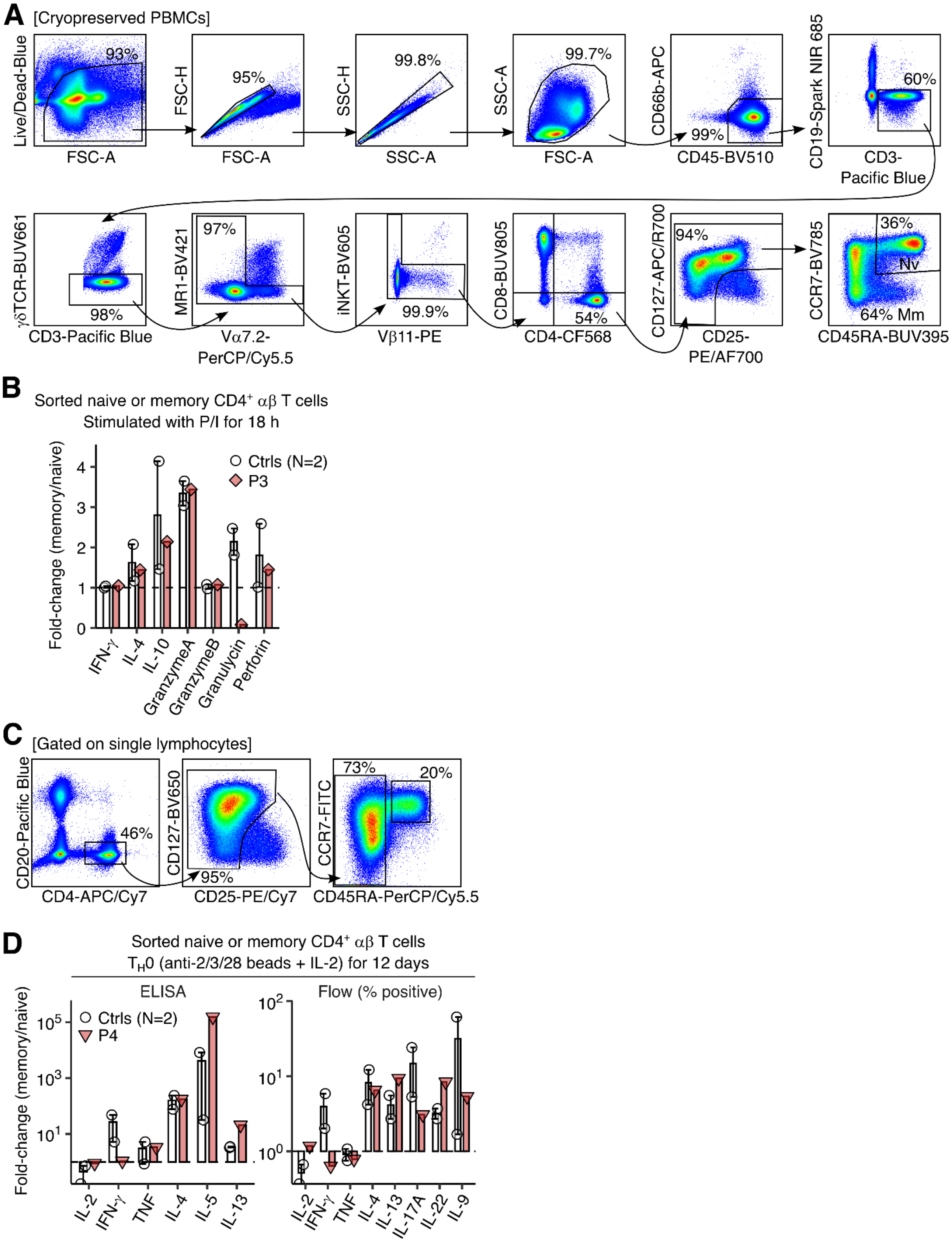
A
[Cryopreserved PBMCs]
Live/Dead-Blue
FSC-A
93%
FSC-H
95%
SSC-H
SSC-A
99.8%
99.7%
CD66b-APC
CD45-BV510
99%
CD19-Spark NIR 685
CD3-Pacific Blue
60%
γδTCR-BUV661
CD3-Pacific Blue
98%
MR1-BV421
Vα7.2-PerCP/Cy5.5
97%
iNKT-BV605
Vβ11-PE
99.9%
CD8-BUV805
CD4-CF568
54%
CD127-APC/R700
CD25-PE/AF700
94%
CCR7-BV785
CD45RA-BUV395
36%
Nv
64% Mm
B
Sorted naive or memory CD4⁺ αβ T cells
Stimulated with P/I for 18 h
Fold-change (memory/naive)
Ctrls (N=2)
P3
IFN-γ
IL-4
IL-10
GranzymeA
GranzymeB
Granulycin
Perforin
C
[Gated on single lymphocytes]
CD20-Pacific Blue
CD4-APC/Cy7
46%
CD127-BV650
CD25-PE/Cy7
95%
CCR7-FITC
CD45RA-PerCP/Cy5.5
73%
20%
D
Sorted naive or memory CD4⁺ αβ T cells
TH0 (anti-2/3/28 beads + IL-2) for 12 days
ELISA
Flow (% positive)
Fold-change (memory/naive)
Ctrls (N=2)
P4
IL-2
IFN-γ
TNF
IL-4
IL-5
IL-13
IL-17A
IL-22
IL-9

Figure S15. Analysis of naïve and memory CD4$^+$ T lymphocytes in LY9 deficiency. (A) Gating strategy for naive and memory CD4$^+$ αβ T lymphocytes. (B) Naïve and memory CD4$^+$ T lymphocytes sorted from the PBMCs of P3 (aged 40 years) and two healthy donors were stimulated with P/I for 18 hours. The production of cytokines and soluble mediators was assessed in a LEGENDplex assay. (C and D) Naïve and memory CD4$^+$ T lymphocytes sorted from the PBMCs of P4 (aged 17 years) and two healthy donors were cultured for 12 days under T_H0 conditions (i.e., anti-CD2/3/28 mAb beads plus IL-2). The production of cytokines was assessed by multiplex ELISA and flow cytometry at the end of culture. (C) Sorting strategy. (D) Results. The memory-to-naïve fold-change was calculated to assess the acquisition of cytokine-producing capacity in memory CD4$^+$ T lymphocytes relative to their naïve counterparts from the same individual. The dashed horizontal line represents 1.

Figure S16

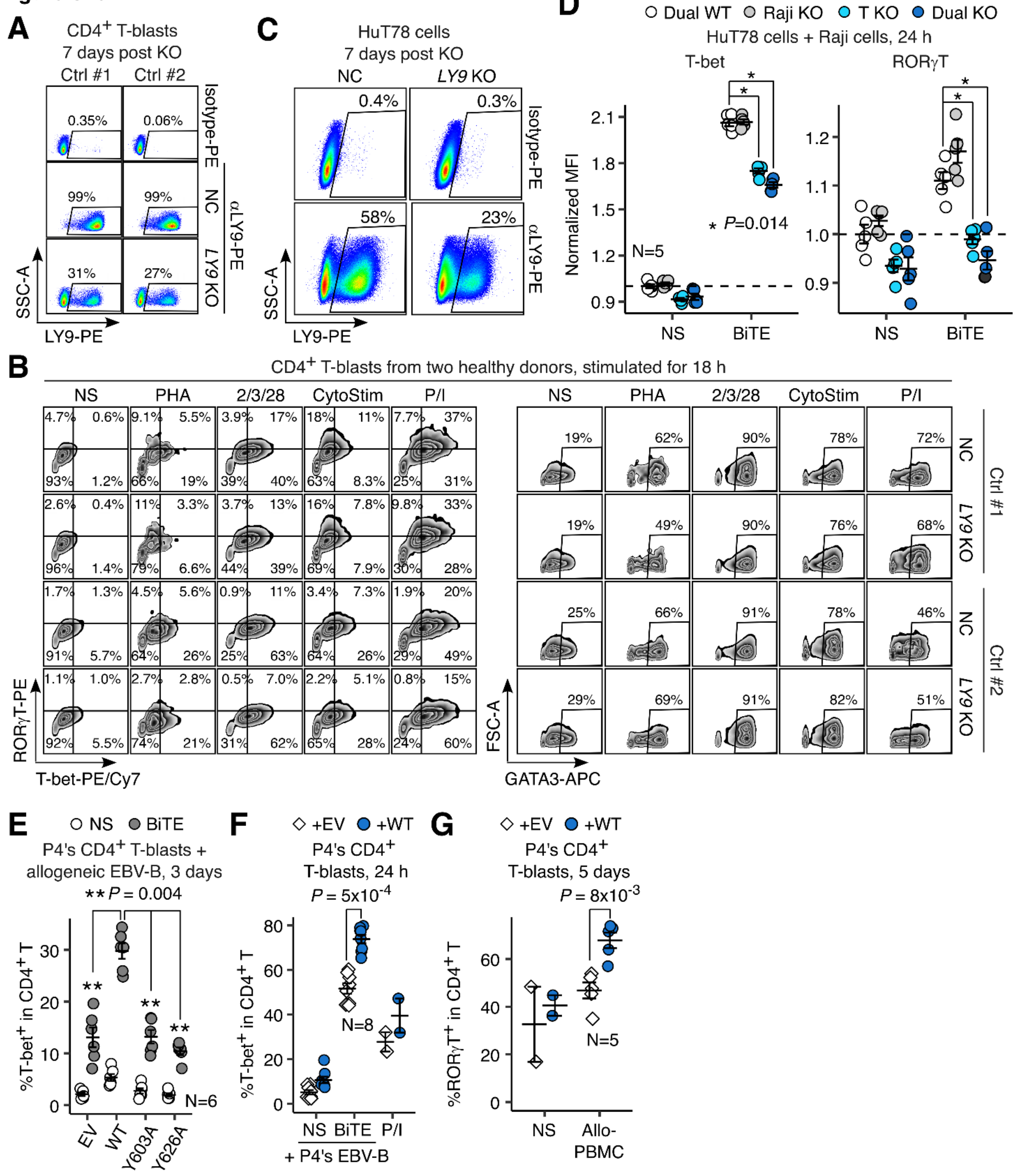

Figure S16. LY9 governs T-bet and RORγT expression in a CD4⁺ T-cell-intrinsic manner.

(A) *LY9* knockout (KO) in $CD4^+$ T-blasts from two healthy donors on day 7 after KO. Cells were nucleofected with Cas9 and either scrambled sgRNA or sgRNA pools for *LY9*, and were then restimulated with anti-CD2/3/28 mAb cocktail and expanded *in vitro* for 28 days after KO. (B)

Expanded $CD4^+$ T-blasts were stimulated with the indicated stimuli for 18 hours and analyzed by flow cytometry. (C) *LY9* KO in HuT78 $CD4^+$ T-lymphoma cells. (D) Coculture assay with HuT78 T-lymphoma and Raji B-lymphoma cells with or without *LY9* KO. For KO cells, enrichment in LY9-negative cells was achieved by FACS. Cells were either left non-stimulated or were stimulated with blinatumomab (anti-CD3-CD19 bispecific T-cell engager; BiTE) for 24 hours. The levels of T-bet and RORγT were determined by flow cytometry. MFI values were normalized against the mean value for non-stimulated cells with no KO. Five technical replicates were prepared. Representative results from two experiments are shown. (E) Coculture assay with allogeneic EBV-B cells and $CD4^+$ T-blasts from P4 transduced with EV, WT LY9, or LY9 mutants. T-bet protein was quantified by flow cytometry. Six technical replicates were prepared. Representative results from two experiments are shown. (F) Coculture assay with $CD4^+$ T-blasts from P4 transduced with EV or WT LY9 and P4's non-modified EBV-B cells. Cells were stimulated with blinatumomab (BiTE) for 24 hours. Transcription factor levels were determined by flow cytometry. Technical replicates (N=8; N=2 for P/I) were prepared. Representative data from two independent experiments are shown. (G) Coculture assay with $CD4^+$ T-blasts from P4 transduced with EV or WT LY9 incubated with allogeneic PBMCs (irradiated and CFSE-labeled) for 5 days. Transcription factor levels in CFSE-negative T-blasts were determined by flow cytometry. Technical replicates were prepared (N=5). Representative data from two independent experiments are shown.

Figure S17

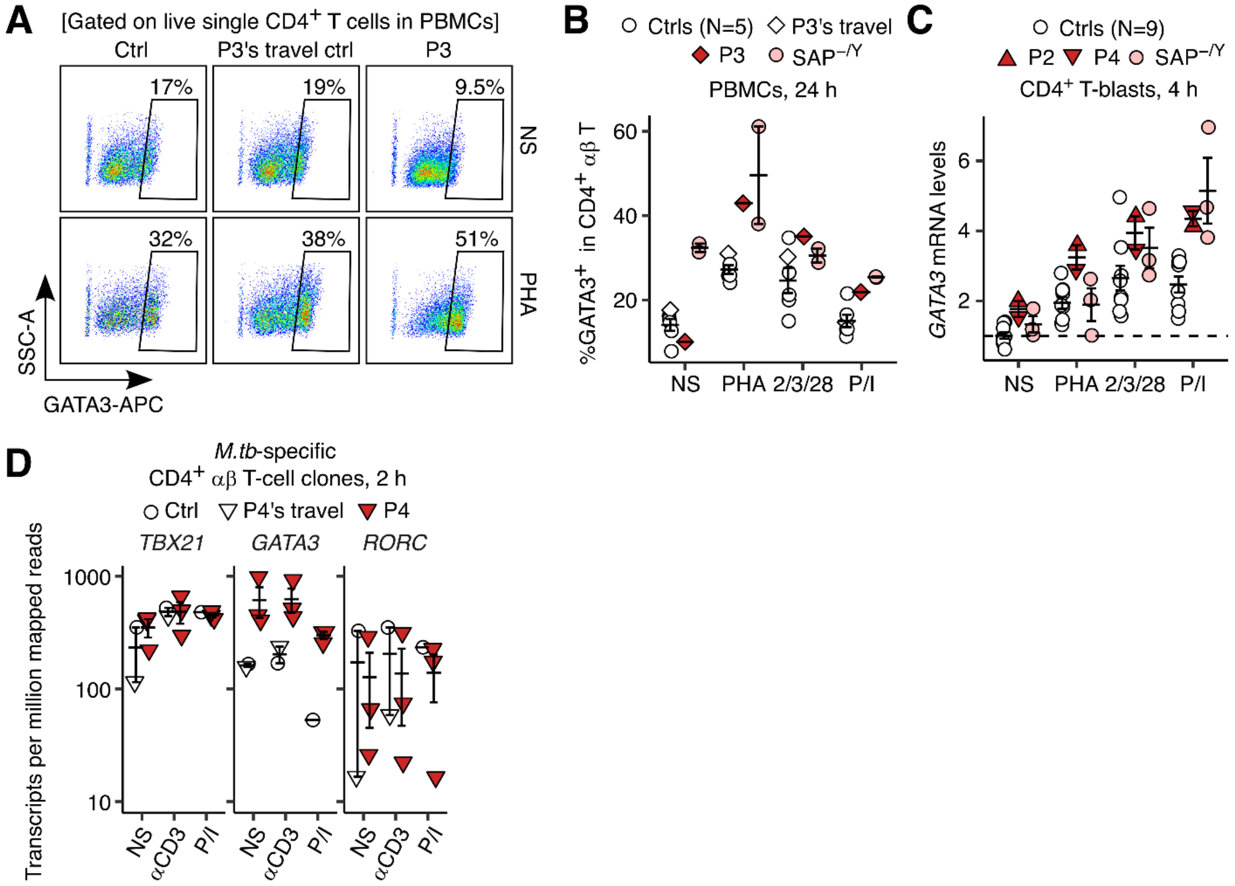

Figure S17. High levels of GATA3 expression in LY9-deficient CD4^{+} T lymphocytes. (A and B) The expression of GATA3 in CD4^{+} αβ T lymphocytes among PBMCs from P3 (aged 40 years), two SAP-deficient patients, and healthy controls was determined by flow cytometry after 24 hours of incubation with or without polyclonal stimuli. (A) Representative results. (B) Summary. Results from three experiments were compiled. Results from technical replicates were averaged. (C) CD4^{+} T-blast stimulation assay. The levels of *GATA3* mRNA were quantified by RT-qPCR, with *GUSB* as an endogenous control. The mRNA levels were normalized against the mean for non-stimulated control cells. (D) Levels of *TBX21*, *GATA3*, and *RORC* mRNA in *M.tb*-specific clones from controls and P4, as determined by RNASeq. In B-D, bars represent the mean and SEM.

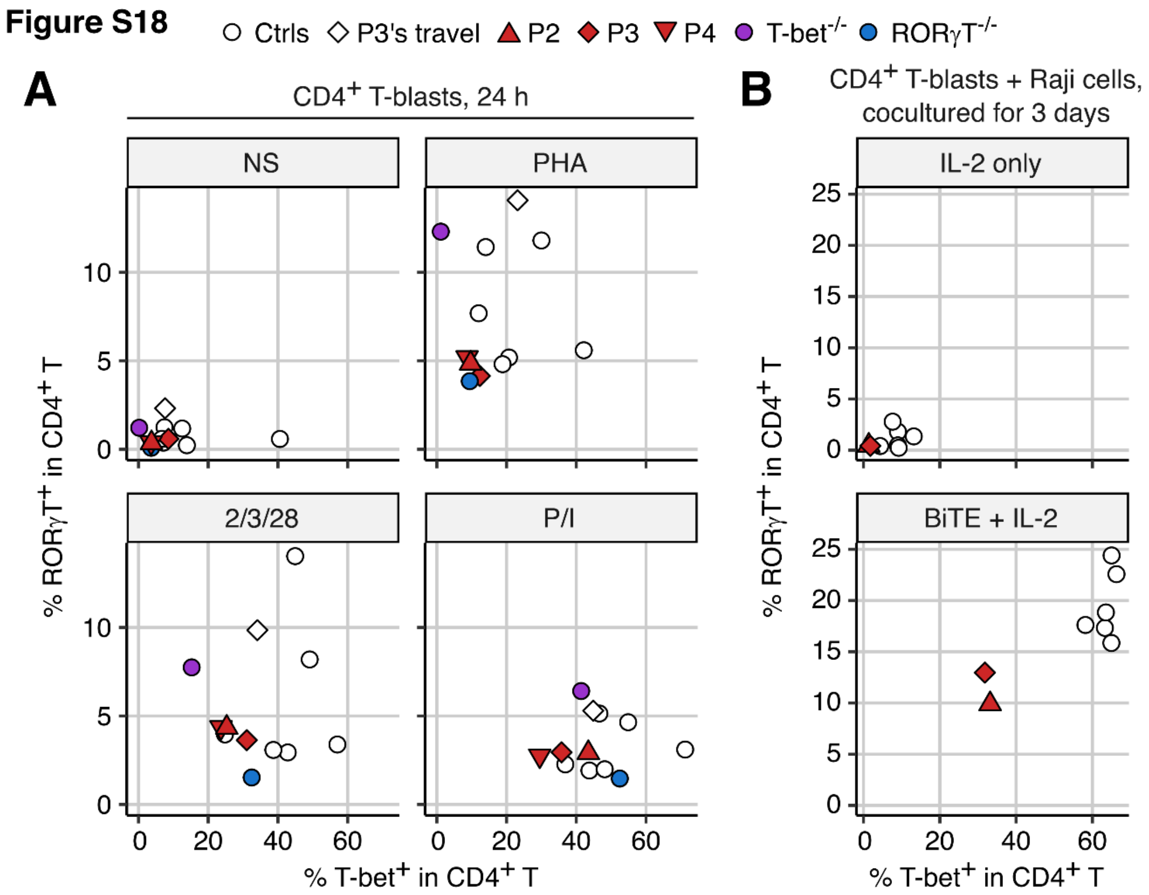

Figure S18. Analysis of T-bet and RORγT expression in cultured LY9-deficient CD4+ T lymphocytes. (A) MACS-enriched CD4+ T-blasts from healthy donors (*N*=6), P3's travel control, P2, P3, P4, one T-bet-deficient patient, and one RORγT-deficient patient were either left non-stimulated or were stimulated with polyclonal stimuli for 24 hours. The levels of T-bet and RORγT were determined by flow cytometry. (B) MACS-enriched CD4+ T-blasts from healthy donors (*N*=6), P2, and P4 were cocultured with Raji B-lymphoma cells. Cells were cultured with IL-2 alone or IL-2 and blinatumomab (BiTE) for 3 days. The levels of T-bet and RORγT were determined by flow cytometry.

Figure S19

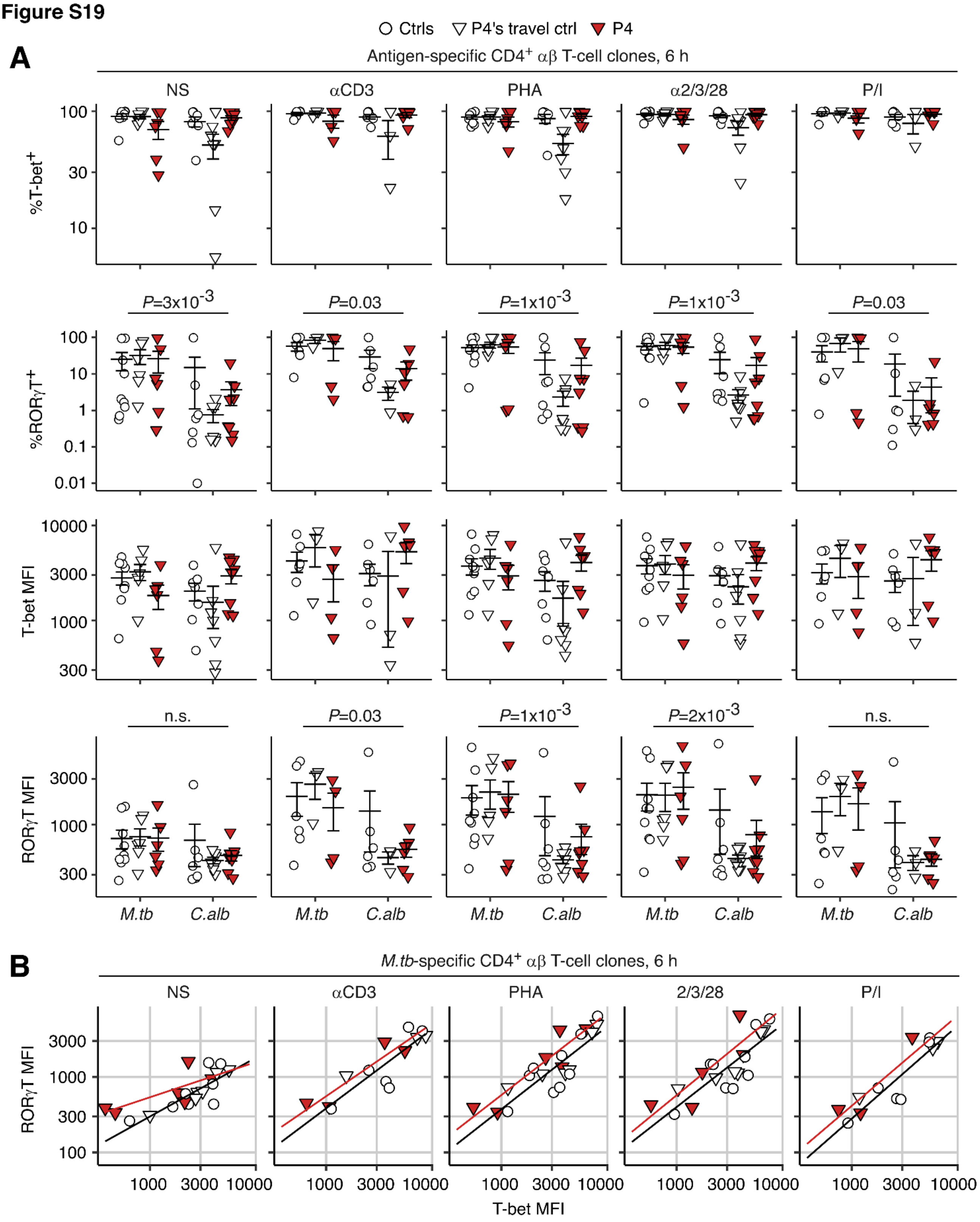

Figure S19. Analysis of T-bet and RORγT levels in LY9-deficient antigen-specific CD4+ αβ T-cell clones. (A) The levels of T-bet and RORγT in antigen-specific CD4+ αβ T-cell clones from P4 (aged 17 years), his travel control, and BCG-vaccinated controls was analyzed by flow cytometry.

Results from two experiments were compiled. For RORγT, statistical significance was determined for the difference between *M.tb*-specific clones and *C.albicans*-specific clones (controls and P4 combined). Bars represent the mean and SEM. (B) Correlation of T-bet and RORγT MFI values. Lines were fitted by linear regression by genotype (i.e., controls or LY9 deficiency). PHA, phytohemagglutinin. P/I, PMA and ionomycin.

Figure S20

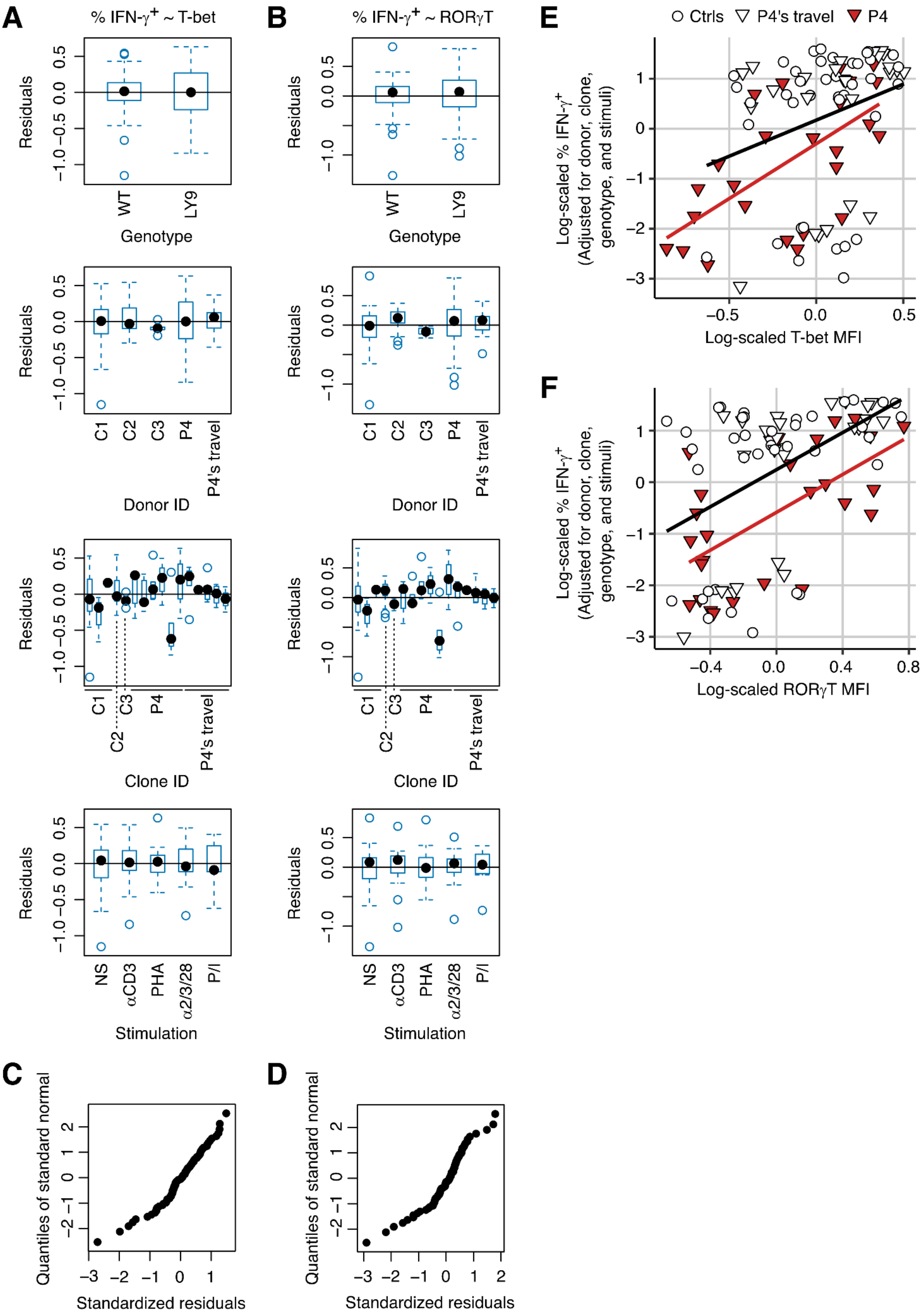
A
% IFN-γ+ ~ T-bet
B
% IFN-γ+ ~ RORγT
Residuals
WT
LY9
Genotype
C1
C2
C3
P4
P4's travel
Donor ID
Clone ID
NS
αCD3
PHA
α2/3/28
P/I
Stimulation
C
D
Quantiles of standard normal
Standardized residuals
E
Ctrls
P4's travel
P4
Log-scaled % IFN-γ+
(Adjusted for donor, clone, genotype, and stimuli)
Log-scaled T-bet MFI
F
Log-scaled RORγT MFI

Figure S20. Correlation between IFN-γ-producing capacity and the levels of T-bet and RORγT in *M.tb*-specific CD4$^+$ αβ T-cell clones. Linear mixed-effects models were fitted to the log-transformed percentage of IFN-γ-producing cells. The MFI for T-bet or RORγT and genotype (i.e., controls or LY9 deficiency) were incorporated as fixed effects, whereas donor ID, clone ID, and stimulus were incorporated as random effects. (A and B) Fitting of the residual distribution for the (A) T-bet and (B) RORγT models. (C and D) Quantile-quantile plots for the normality of residual fitting for (C) T-bet and (D) RORγT. (E and F) The percentage of IFN-γ-producing cells as a function of (E) T-bet and (F) RORγT levels. The MFI for T-bet or RORγT was significantly associated with enhanced IFN-γ production in the linear mixed-effects models ($P < 1 \times 10^{-4}$, conditional $R^2 = 0.92$ for T-bet; $P < 1 \times 10^{-4}$, conditional $R^2 = 0.90$ for RORγT). In these models, LY9 deficiency is associated with a significantly lower level of IFN-γ production ($P = 6 \times 10^{-3}$ and 7×10^{-4} for T-bet and RORγT, respectively, by likelihood ratio tests). PHA, phytohemagglutinin. P/I, PMA and ionomycin.

Figure S21

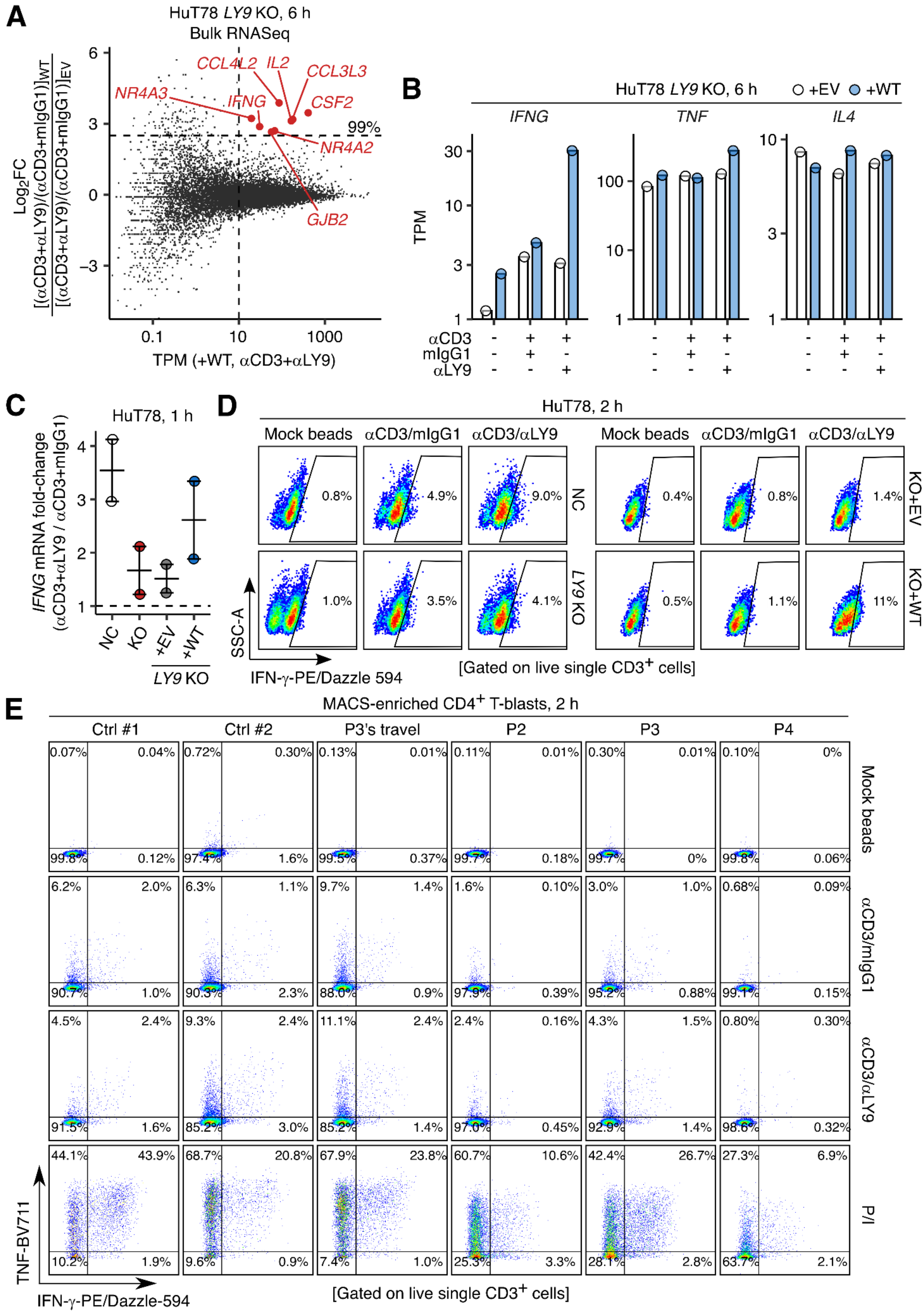
A
HuT78 LY9 KO, 6 h
Bulk RNASeq
Log2FC
[(αCD3+αLY9)/(αCD3+mIgG1)]WT / [(αCD3+αLY9)/(αCD3+mIgG1)]EV
TPM (+WT, αCD3+αLY9)
NR4A3
CCL4L2
IL2
CCL3L3
IFNG
CSF2
99%
NR4A2
GJB2
B
HuT78 LY9 KO, 6 h
+EV
+WT
IFNG
TNF
IL4
TPM
αCD3
mIgG1
αLY9
C
HuT78, 1 h
IFNG mRNA fold-change
(αCD3+αLY9 / αCD3+mIgG1)
NC
KO
+EV
+WT
LY9 KO
D
HuT78, 2 h
Mock beads
αCD3/mIgG1
αCD3/αLY9
NC
LY9 KO
KO+EV
KO+WT
SSC-A
IFN-γ-PE/Dazzle 594
[Gated on live single CD3+ cells]
E
MACS-enriched CD4+ T-blasts, 2 h
Ctrl #1
Ctrl #2
P3's travel
P2
P3
P4
Mock beads
αCD3/mIgG1
αCD3/αLY9
P/I
TNF-BV711
IFN-γ-PE/Dazzle-594
[Gated on live single CD3+ cells]

Figure S21. Enhanced IFN-γ production through LY9 costimulation in $CD4^+$ T lymphocytes. LY9 crosslinking was achieved by incubating $CD4^+$ T cells with mock magnetic beads or beads conjugated with anti-CD3 and anti-LY9 mAb or mouse IgG1 isotype control. (A) Bulk RNASeq. *LY9* KO HuT78 T-lymphoma cells lentivirally transduced with EV or WT LY9 were stimulated for 6 hours. The TPM in WT-transduced cells after stimulation with anti-CD3 plus anti-LY9 beads is plotted on the *x*-axis. The $\log_2$FC in the LY9-crosslinking-dependent induction in WT-transduced cells divided by that in EV-transduced cells is shown on the *y*-axis. Genes with TPMs greater than 10 and $\log_2$FC values greater than the 99th percentile are shown in color. (B) TPM of representative genes. (C) RT-qPCR analysis of HuT78 T-lymphoma cells with or without *LY9* KO and lentiviral transduction with EV or WT LY9. Results from two experiments are compiled. (D) Representative results of the LY9 crosslinking assay. IFN-γ production was determined by flow cytometry. (E) LY9 crosslinking assay on MACS-enriched $CD4^+$ T-blasts from three healthy controls, P2, P3, and P4.

Figure S22

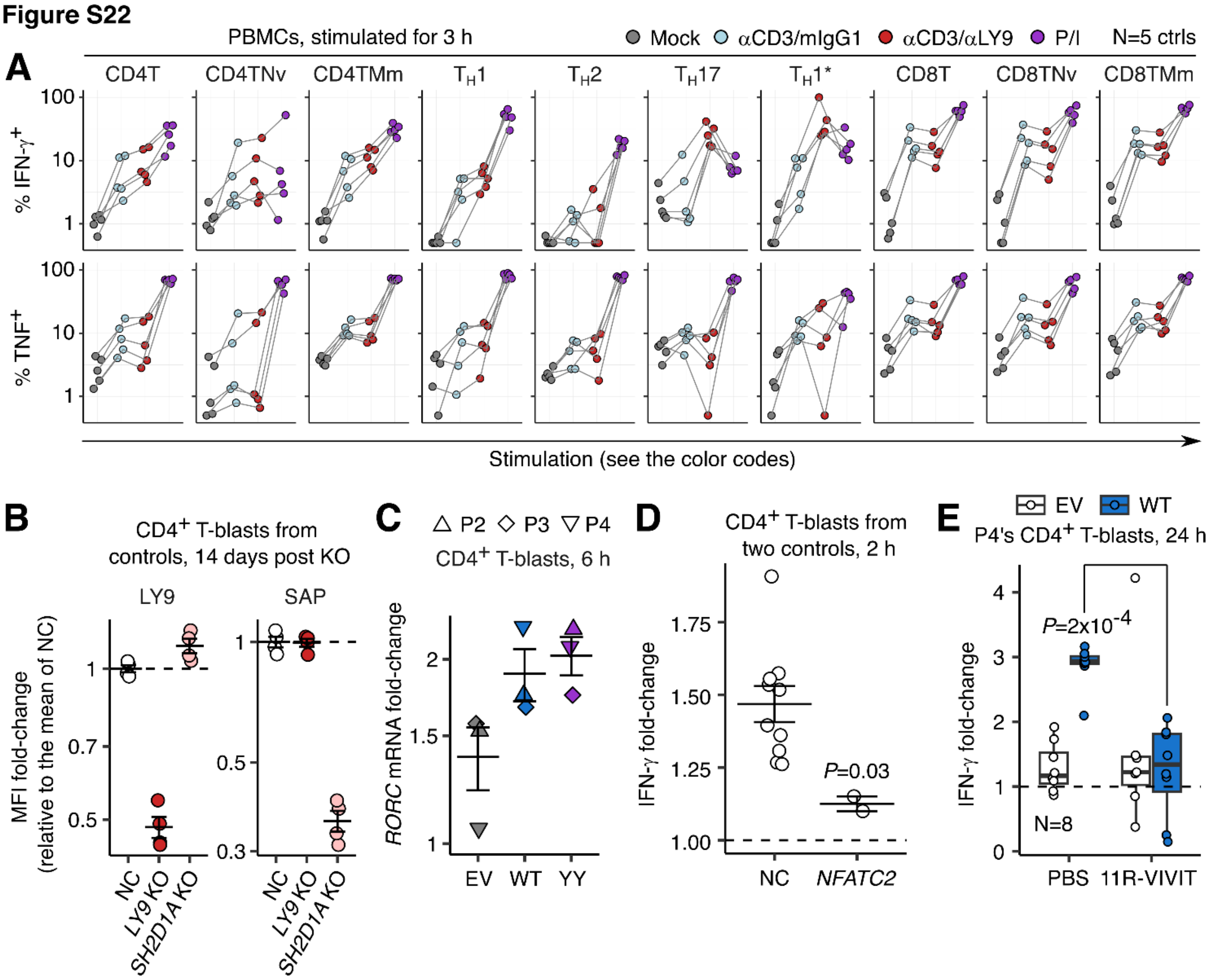

Figure S22. Enhanced IFN-γ production through LY9 costimulation in T_H1* cells. (A) Cytokine production by T-cell subsets of PBMCs from five healthy donors upon stimulation with mock magnetic beads or beads conjugated with anti-CD3 and anti-LY9 mAb or mouse IgG1 isotype control. P/I was used as a positive control. Cytokine production was quantified by intracellular flow cytometry. (B) *LY9* or *SH2D1A* knockout (KO) in $CD4^+$ T-blasts from two healthy donors. Cells were nucleofected with Cas9 and either scrambled sgRNA or sgRNA pools for *LY9* or *SH2D1A* and expanded for 14 days. Technical duplicates were prepared for each sgRNA nucleofection. Protein levels were determined by flow cytometry. The median fluorescence intensity (MFI) values were normalized against scrambled sgRNA controls. (C) *RORC* mRNA levels in $CD4^+$ T-blasts from P2, P3, and P4 lentivirally transduced with EV, WT LY9, or LY9 with Y603A/Y626A substitutions (abbreviated YY),

as determined by RT-qPCR. *GUSB* was used as an endogenous control. Fold-changes in mRNA levels (value obtained with anti-CD3 plus anti-LY9 antibodies divided by the value obtained with anti-CD3 antibody plus isotype control) are shown. (D) *NFATC2* knockdown. $CD4^+$ T-blasts from one healthy donor were lentivirally transduced with five different negative control shRNAs (combined as “NC”) or *NFATC2* shRNA and subjected to selected with puromycin. Fold-changes in the % IFN-γ^+ cells (value obtained with anti-CD3 plus anti-LY9 antibodies divided by the value obtained with anti-CD3 antibody plus isotype control), as determined by flow cytometry, are shown. Technical duplicates were prepared. (E) NFAT inhibition assay. $CD4^+$ T-blasts from P4 lentivirally transduced with EV or WT LY9 were incubated with or without a cell-permeant NFAT inhibitor (11R-VIVIT peptide; 10 μM in PBS). Fold-changes in the levels of IFN-γ secreted (value obtained with anti-CD3 plus anti-LY9 antibodies divided by the value obtained with anti-CD3 antibody plus isotype control), as determined in a LEGENDplex assay, are shown. Results from two experiments were compiled, with four technical replicates per condition in each batch. In B-D, bars represent the mean and SEM.

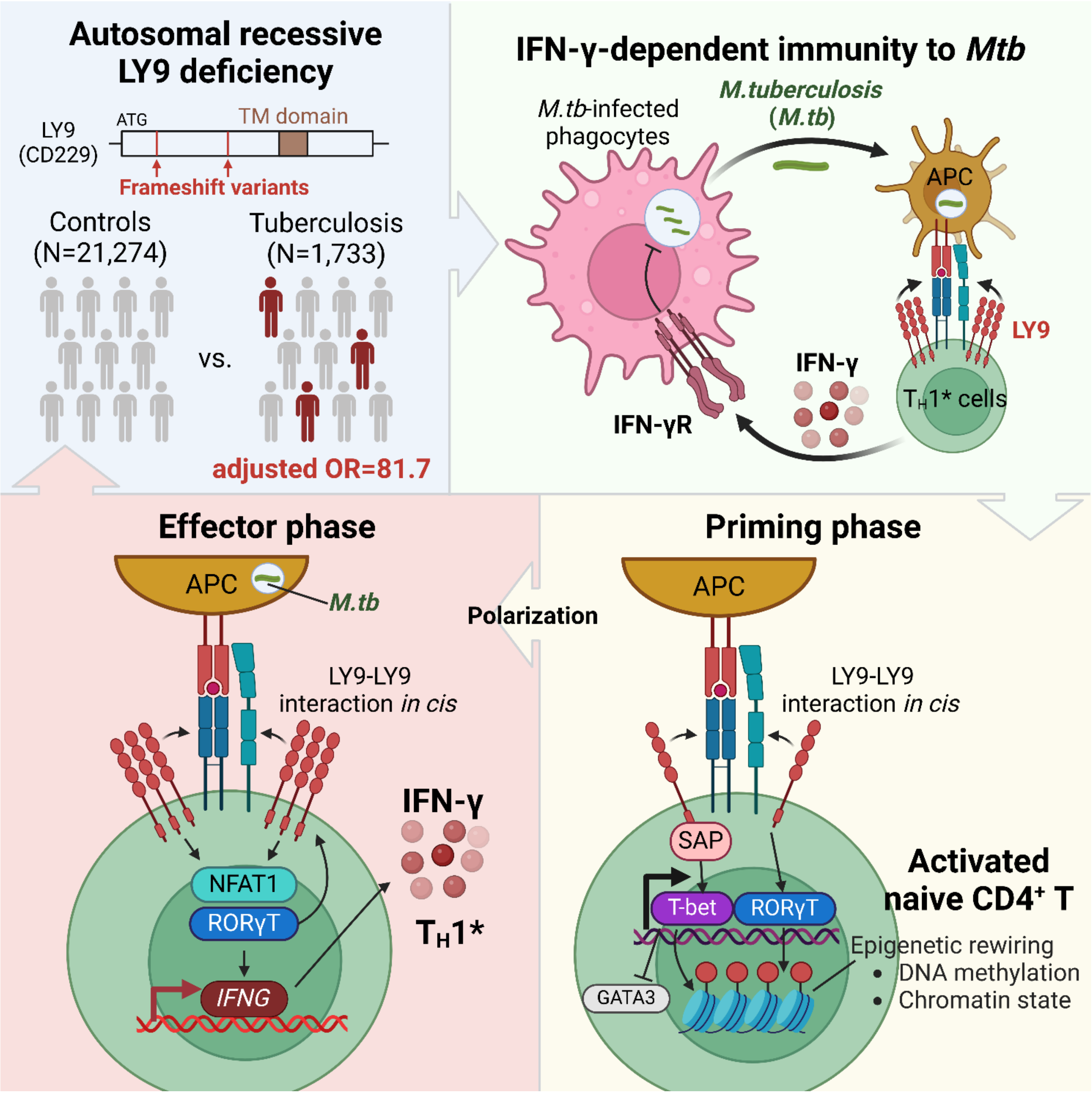

Figure S23. Graphical summary.

Supplementary Tables

Table S1. Enrichment of our in-house TB cohort in *LY9* variants relative to healthy controls or patients with non-mycobacterial infectious diseases under various models. OR, odds ratio; CI, confidence interval. The analysis was adjusted for the first five principal components (PCs, based on principal component analysis (PCA) on the whole-exome sequencing (WES) data) to account for the ethnic heterogeneity of the samples. Principal component analysis was performed with Plink v1.9 software on WES data, using 16,730 exonic variants with minor allele frequency > 1% and a call rate > 98%. OR was adjusted for sex and ethnicity by Firth's penalized logistic regression (*75*). Only the recessive model for pLOF variants was statistically significant, given that the Bonferroni-corrected statistical significance threshold for six independent tests was 0.05/6 = 0.0087.

LY9	Model	N carriers in TB cohort	N carriers among controls	Adjusted OR [95% CI]	*P* value
**pLOF**	**Recessive**	**3/1733**	**0/21274**	**81.7 [7.90 - 10986.5]**	**1.2×10^{-4}**
	Dominant	26/1733	173/21274	1.70 [1.09 – 2.55]	0.020
Inframe/missense GnomAD v2.1 freq < 5%	Recessive	6/1733	54/21274	0.82 [0.33 – 1.75]	0.63
	Dominant	213/1733	1862/21274	1.10 [0.94 – 1.29]	0.24
Synonymous GnomAD v2.1 freq < 5%	Recessive	0/1733	6/21274	0.49 [0.0037 – 4.23]	0.59
	Dominant	29/1733	411/21274	0.66 [0.44 – 0.96]	0.027

Table S2. Laboratory test results for P1 at the age of two months

**Parameter**	**Result**
Hemoglobin concentration (g/dL)	12.0
Mean corpuscular volume (fL)	94.6
Mean corpuscular hemoglobin (pg)	30.6
Platelets ($10^3/\mu L$)	484
WBCs ($10^3/\mu L$)	19.8
Neutrophils ($10^3/\mu L$)	5.03
Eosinophils ($10^3/\mu L$)	0.32
Basophils ($10^3/\mu L$)	0.08
Monocytes ($10^3/\mu L$)	1.87
Lymphocytes ($10^3/\mu L$)	12.5
Total protein (g/L)	48
Albumin (g/L)	25.7
AST (U/L)	62
ALT (U/L)	52
CRP (mg/L)	6.1
Sodium (mmol/L)	139
Potassium (mmol/L)	5.70

Table S3. Laboratory test results for P4 at the age 16 years

Parameter	Result	Reference
RBC (10^6/µL)	5.93	4.03-5.29
Hemoglobin concentration (g/dL)	17.5	11.0-14.5
Hematocrit (%)	50.6	33.9-43.5
Platelets (10^3/µL)	254	175-332
WBCs (10^3/µL)	6.52	3.84-9.84
Neutrophils (10^3/µL)	4.31	2.73-6.68
Eosinophils (10^3/µL)	0.09	0.04-0.20
Basophils (10^3/µL)	0.03	0-0.8
Monocytes (10^3/µL)	0.38	0.4-1.3
Lymphocytes (10^3/µL)	1.71	1.03-2.18
Anti-HIV Ab	0.220	< 0.9
Anti-HAV IgM	0.283	< 0.99
Anti-HAV IgG	1.13	> 1
HBsAg	0.32	< 0.89
HBsAb	2.0	< 10
Anti-HBe Ab	1.28	> 1
Anti-HBc IgM	0.0691	< 1
Anti-HBc IgG	2.03	> 1
Anti-HCV Ab	0.030	< 0.9
Anti-CMV IgM	0.137	< 0.7
Anti-CMV IgG	500	< 0.5
Anti-Rubella IgM	0.22	< 0.8
Anti-Rubella IgG	135	< 10
Anti-EBV VCA IgM	0.08	< 0.11
Anti-EBV VCA IgG	3.8	< 0.10
Anti-EBV EBNA IgG	6.11	< 0.10
Anti-Herpes simplex type 1 IgM	2.97	< 1
Anti-Herpes simplex type 1 IgG	0.51	< 1
Anti-Herpes simplex type 2 IgM	1.38	< 1
Anti-Herpes simplex type 2 IgG	0.17	< 1
